# Speaker Abstracts

**DOI:** 10.1002/jia2.25093

**Published:** 2018-04-19

## KL111

### What is needed to end HIV/AIDS?

**D Daskalakis**

New York City Department of Health and Mental Hygiene, New York, NY, USA

New York City, the historical epicenter of the HIV epidemic in the United States, has launched a programme called “Ending the Epidemic”, or “EtE” a jurisdictional strategy to drive new HIV infections to below epidemic levels. Statewide, ending the epidemic means reducing new infections to less than 750 by 2020. For New York City, which represents 80% of the epidemic in the State, our goal is 600 or fewer new infections by this date. Using the New York State “Blueprint” as its foundation, this strategy rapidly implements advances in HIV treatment, prevention and surveillance to address issues of health equity and access that hinder progress toward the end of AIDS. Scientific advances, community mobilization and political groups will have aligned to create programmes and interventions that supplement the already robust work being done in this large jurisdiction. This presentation will highlight the process of generating this strategy, present components of the New York City approach to ending the HIV epidemic, and will share preliminary data from these programmes where possible.

## KL112

### What we mean in our region by ending AIDS?

**JW Pape**

Les Centres, GHESKIO, Port‐au‐Prince, Haiti, and Center for Global Health, Division of Infectious Diseases, Weill Cornell Medicine, Cornell University, New York, NY, USA

“Ending AIDS” is the new slogan enshrined in the Sustainable Development Goals (SDGs) that stipulates: “…by 2030, end the epidemics of HIV and AIDS, tuberculosis, malaria and neglected tropical diseases and combat hepatitis, water‐borne diseases and other communicable diseases” (goal 3 on disease control). Is this policy rhetoric? Different experts and agencies seem to have different perspectives. All agree that an “AIDS‐free generation” in which antiretroviral (ART) drugs prevent transmission to babies and allow HIV‐infected persons to live near‐normal lifespans, is possible. The pessimists think that “An AIDS‐free world is a nonsense, irresponsible and dangerous and may lead to complacency. The best we can hope for is low endemic levels”. They believe that we need to finish a battle that is only half way won. To support their view, they mention three arguments: the limited AIDS budget, the neglect of key populations and logistical issues. Since 2008 the AIDS budget has been flat, whereas it should have increased. Stigma and discrimination prevent men who have sex with men, intravenous drug users, migrants, prisoners, sex workers and street children to have full access to ART care. The logistical challenges make it difficult to place the other 50% of HIV‐infected persons on ART as they are the most difficult to diagnose. Adherence issues are important as well as comorbidities related to long‐term treatment. The optimists feel that they have the stimulus to end the epidemic. Indeed, the AIDS epidemic has been halted. In 2000, Millennium Development Goal 6.1 called for a halt and reversal of the spread of HIV and AIDS. At the time it was felt to be audacious and completely out of reach! However, this seemingly insurmountable goal was reached. Indeed, new HIV infections in 2014 have been reduced by 32%. Furthermore, over 16 million are on ART in sub‐Saharan Africa in 2017 and 7.8 million deaths have been averted due to increased ART coverage. These achievements have led to a more ambitious goal of “ending HIV and AIDS as a public threat” by 2030. That is reducing HIV infections, stigma and discrimination experienced by people living with HIV and AIDS and key populations and AIDS‐related deaths by 90%, such that AIDS no longer represents a major threat to any population or country. Some feel that the end of AIDS should be unlike any other “end” of a health condition. Just as the HIV response has been radical and different. The presentation will review the tools available and what it will take to end AIDS as a public threat.

## KL121

### The microbiome in HIV infection

**S Pinto‐Cardoso**

Center for Research in Infectious Diseases (CIENI), National Institute of Respiratory Diseases (INER), Mexico City, Mexico

Alterations in the enteric microbiome have been reported in HIV infection and have been associated with both HIV acquisition and pathogenesis (disease progression and non‐communicable diseases). Our understanding of how microbial communities structure and function and confounding factors such as diet, antibiotic usage, sexual practices influencing HIV infection is steadily growing. I will discuss how integrating metagenomics with viromic analysis (virome), measures of host response (immune system, clinical outcomes, metadata‐rich cohorts) and bioinformatics strategies has helped us contributing towards generating knowledge on the microbiome in health and disease (HIV infection), to better interpret results and finally how these integrated approaches might help us identify biomarkers of microbial dysbiosis that might be targets for therapeutic interventions. Our unique setting; the research laboratory adjacent to the HIV clinic and the viral diagnostic laboratory, offers a unique perspective and opportunity to study different aspects of the microbiome in HIV infection; from HIV‐associated immunodeficiency, immune reconstitution to changes associated with initiation of antiretroviral therapy, and much more. So far, our results have shown that HIV infection is associated with reduced microbial diversity, the depletion of key commensal bacteria; in particular those capable of producing the short‐chain fatty acid butyrate, expansion of the enteric virome, differential effects of antiretroviral therapy on both the enteric microbiome and markers of microbial translocation and epithelial barrier damage. In addition, a brief overview of other ongoing projects looking at different anatomical sites and preliminary results on our pre‐prophylaxis exposure (PrEP) cohort study will be given.

## KL122

### Early treatment of HIV in infants

**KA Nielsen**

Ronald Reagan UCLA Medical Center, Los Angeles, CA, USA

Since the first paediatric AIDS case was reported in 1982, significant strides have been made in the prevention and treatment of perinatally acquired HIV infection. From transmission rates as high as 40% if cumulative in utero, intrapartum and postpartum mother to child HIV transmission (MTCT) are computed in the absence of any intervention, today, with adequate maternal diagnosis and prompt initiation of antiretroviral treatment, HIV MTCT rates are 0.5% or lower. Although the number of cases of paediatric HIV infection declined dramatically in the last 20 years, the decline has not been as steep in resource limited settings, where many infants still become infected. Remaining challenges for elimination of mother to child transmission include the risk of breastfeeding transmission and maternal HIV acquisition during pregnancy or while breastfeeding. Guidelines for prompt initiation of treatment in HIV infected infants have been in place for years and are based on data demonstrating high mortality if treatment is deferred. Early intense antiretroviral treatment relies on prompt identification of infection and requires close follow‐up in the first weeks of life. Very early treatment of infants is analogous to treatment of acute HIV infection in older populations and significantly reduces viral reservoir burden. This approach spares damage to the developing immune system and may pave way for functional cure interventions. Nevertheless, early treatment of infant HIV infection is challenging due to diagnostic difficulties and lack of adequate paediatric antiretroviral formulations. The evolution of treatment strategies in perinatal/paediatric HIV infection in the Americas will be reviewed.

## KL211

### Update on PEP and on its role in the era of PrEP

**DK Smith**

Centers for Disease Control and Prevention (CDC), Atlanta, GA, USA

In the United States, non‐occupational post‐exposure prophylaxis (nPEP) is supported to reduce the risk of HIV acquisition in persons with potential HIV exposure. nPEP has clear individual benefits, but is unlikely to significantly reduce new HIV diagnoses at the population level. Making urgent and affordable access to nPEP available remains an issue in the US. Lack of awareness of pre‐exposure prophylaxis (PrEP) both by clinicians and underserved populations where high rates of new diagnoses is common. However, as PrEP is being scaled‐up, awareness and use of nPEP is also increasing because of coverage of interventions in media campaigns and provider education efforts. HIV screening is required before providing either nPEP or PrEP and will identify some persons with unrecognized HIV infection who can be rapidly linked to HIV treatment. Persons who have requested multiple courses of nPEP or whose reported sexual behaviours when assessed for nPEP suggest likely frequent HIV exposures, are excellent candidates for PrEP use. These patients can be easily transitioned at the completion of a 28‐day nPEP course to ongoing PrEP use. While men who have sex with men (MSM) are the transmission risk group with highest rates of new HIV diagnoses and highest use of nPEP and PrEP use, African American MSM and women are the racial/ethnic group with the lowest use of nPEP and PrEP and the highest lifetime risk of acquiring HIV infection. Population impact of nPEP and PrEP use in reducing new HIV infections can only be achieved if we also reduce racial disparities in their use.

## Oral Abstracts

## O11 – Role of Integrase Inhibitors in LATAM: Are We Ready for First‐Line?

## O111

### Current role of integrase inhibitors in Latin American guidelines

**E Martínez‐Buitrago**

Infectious Diseases, Santiago de Cali, Colombia

In 2017 integrase inhibitors (INSTI) fulfilled 10 years of use in clinical practice in the management of HIV patients and their introduction has been followed by a rapid adoption in most international guidelines in developed countries. From 2010, guidelines such as Department of Health and Human Services, International AIDS Society, European AIDS Clinical Society, Spanish AIDS Study Group and the British HIV Association have already pointed to the INSTI as first‐line therapies, and more recently committed almost exclusively to this therapeutic class as the only preferred choice for the initial treatment. Despite these advances, World Health Organization (WHO) and Latin America have had a slow process of inclusion of the INSTI in their guidelines from 2013. WHO recommends the use of dolutegravir as an alternative, but only since the end of 2015. Countries with gradual adoption of INSTI include Chile in 2013 (only RAL), Mexico, Colombia and Venezuela in 2014 (all RAL), Argentina in 2016 (all 3 INSTI) and Brazil in 2017 incorporates them in their preferred options supported with a large‐scale purchase of dolutegravir. Overall, the use of INSTI in Latin America remains very limited and scarce in real practice, because of access and price barriers mainly. In 2017 the Pan American Association of Infectology issued standards for the diagnosis and treatment of HIV for Latin America in which INSTI are widely recommended in a variety of clinical scenarios including first‐line. It is striking how recently the governments of Argentina, Chile and Colombia, among others, have facilitated access to the INSTI through administrative and programmatic rules that will surely result in better and greater use in their patient populations.

## O112

### Observational studies with integrase inhibitors and experience with first‐line use in Brazil

**R Sobhie Diaz, AR Pascom, F Rick and A Schwartz Benzaken**

Federal University of São Paulo, São Paulo, Brazil

The majority of guidelines for first‐line antiretroviral treatment converge for universal treatment, use of schemes with reduced number of pills/doses, less toxic NRTIs, and use of integrase inhibitors. Integrase inhibitors are notorious for the low toxicity, and its high efficacy, since the effect on viral load reduction is extremely fast, which sometimes poses some concern in the emergence on immune reconstitution inflammatory syndrome. Second generation integrase inhibitors such as dolutegravir (DTG) and bictegravir are notorious by the unprecedented high potency and a high genetic barrier to resistance. Nonetheless, the experience with integrase inhibitors for first line treatment in resource‐limited settings is limited, the major constraint being cost. Botswana and Brazil included DTG as first‐line treatment in 2017 in substitution to Efavirenz (EFV) based regimens. As of July 2017, approximately 30,000 individuals started treatment with DTG in Botswana, and 90% of individuals achieved viral load below detection limits by three months of treatment, with less than 1% of toxicities/side effects [1]. During 2017, 52,763 adults started DTG as initial treatment in Brazil, compared to 57,535 that started EFV during 2016. After 10‐11 months of ART initiation, 88% of DTG treated individuals presented VL<50copies/mL compared to 83% of EFZ. 81% of DTG treated individuals viral load below 50 copies/mL after three months of treatment compared to 61% among those with EFZ (Figure 1). Side effects have been reported in 3% of individuals receiving DTG.

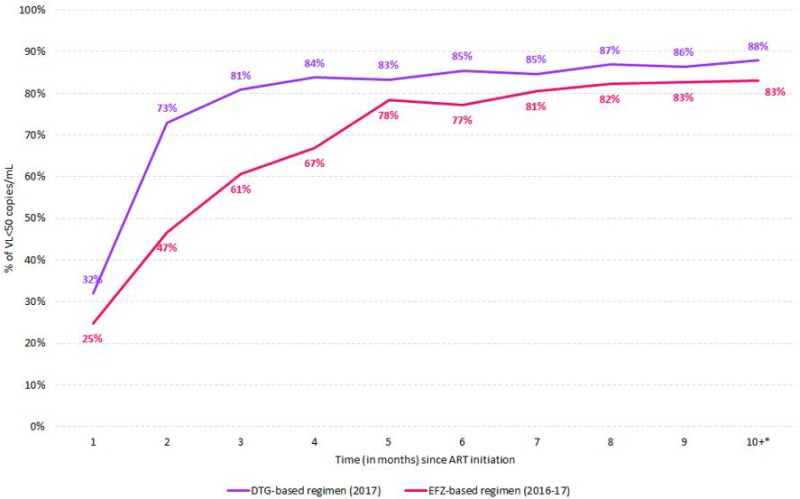

**Abstract O112‐Figure 1. Viral loads below detection limits after initial treatment with dolutegravir (DTG) versus efavirenz (EFV)**

**Reference**

1. Jibril H. Enhanced ARV monitoring in countries: Botswana. SUSA1308, IAS 2017, 23 July 2017

## O113

### The public health response to rising pre‐treatment HIV drug resistance levels in Latin America: the case of Mexico

**S Ávila‐Ríos^1^, C García‐Morales^1^, M Valenzuela‐Lara^2^, D Tapia‐Trejo^1^, M Pérez‐García^1^, DM López‐Sánchez^1^, L Maza‐Sánchez^1^, AC Girón‐Callejas^3^, EA León‐Juárez^2^, C Magis‐Rodríguez^2^, G Reyes‐Terán^1^ and on behalf of the Mexico HIVDR Surveillance Network**

^1^Center for Research in Infectious Diseases CIENI, National Institute of Respiratory Diseases INER, Mexico City, Mexico. ^2^National Center for AIDS Prevention and Control CENSIDA, Mexico City, Mexico. ^3^Universidad del Valle de Guatemala, Guatemala City, Guatemala

**Background**

HIV pre‐treatment drug resistance (PDR) to non‐nucleoside reverse transcriptase inhibitors (NNRTI) in persons initiating or re‐initiating antiretroviral therapy (ART) is increasing substantially in Latin America, and has already crossed the 10% prevalence threshold in several countries. The World Health Organization (WHO) Global Action Plan on HIV drug resistance (HIVDR) recommends implementing nationally representative HIVDR surveys in order to inform public health policy. We compare results on nationally representative published and unpublished PDR surveys carried out between 2015 and 2017 and discuss the option of baseline HIVDR screening generalization vs. avoidance of NNRTI in first‐line regimens in the region, focusing on the case of Mexico.

**Methods**

A large PDR survey was implemented in Mexico in 2017 with sub‐regional representativeness in eight regions of the country, including all clinics starting ART and allocating sample size per clinic using the probability‐proportional‐to‐size method. HIV PDR levels were estimated from pol sequences according to WHO criteria, considering viruses with a Stanford score >15 to efavirenz, nevirapine, any nucleoside reverse transcriptase inhibitor (NRTI), atazanavir, lopinavir or darunavir as resistant.

**Results**

From September to December 2017, 2182 participants were recruited from 71 clinics in Mexico. For all regions, PDR to NNRTI was higher than to other drug classes (*p* < 0.0001). We observed significantly higher levels of NNRTI PDR in the South‐West region, including the three poorest states in the country, compared to the reference Centre‐South region including Mexico City (*p* = 0.03). NNRTI PDR was >10% in the Centre‐North, North‐West, East, South‐East and South‐West, remaining under this threshold in the Centre‐South, North‐East and West regions. The proportion of persons re‐initiating ART varied significantly by region from 4% to 16%, with higher prevalence in the North‐East, West, East, and South‐West (compared to the Centre‐South, *p* < 0.01 in all cases). Nevertheless, the proportion of re‐initiators did not correlate with NNRTI PDR level by region (r = 0.3, *p* = 0.5). As expected, PDR levels were significantly higher in re‐initiators compared to ART‐naïve individuals for all drug classes (*p* < 0.01 in all cases).

**Conclusions**

High NNRTI PDR levels in several Latin American countries underscore the need for countries to consider using first‐line regimens that do not include NNRTIs. Costs and lack of infrastructure hinder consideration of baseline HIVDR screening as a feasible option in many countries. Price and licensing negotiations of drug regimens containing integrase inhibitors are warranted. In large and complex countries such as Mexico, diversification of the response based on regional HIVDR prevalence could be considered.

## O12 – TB Diagnosis and Treatment: An Update for the Region

## O121

### New methods for TB diagnosis

**R López**

Tuberculosis Prevention, Control and Elimination, Pan American Health Organization (PAHO), Washington DC, USA

Tuberculosis (TB) continues to be an important Public Health problem in the Americas with 224,000 notified cases out of 274,000 estimated cases for 2016, leaving a gap of 50,000 cases not detected. Given the infectious nature of the disease, prompt diagnosis and treatment is crucial for the prevention, control and eventual elimination of TB. Until 2007 TB diagnosis was mainly based on smear microscopy and culture. Since then, searching for faster and more sensitive diagnostic tests, multiple new methods based on molecular technologies have emerged and the World Health Organization (WHO) has increased the list of approved methods. Most of them also detect drug resistance to first and second line anti‐TB drugs. The main new methods for TB diagnosis will be presented, including the Genotype MTBDRplus, Genotype MTBDRsl and the Gene Xpert MTB/Rif. The latter is the first fully automated PCR system that detects the *Mycobacterium tuberculosis* and rifampicin‐resistance. It provides results in less than two hours. The advantages and disadvantages for the implementation of these methods in low and mid‐income countries will be addressed. Other new TB diagnostic methods in the pipeline will also be presented.

## O122

### Treatment for drug‐resistant TB

**A Pozniak**

Chelsea and Westminster Hospital, London, UK

Multidrug‐resistant (MDR) and extensively drug‐resistant tuberculosis (TB) are recent global health issues, which makes tuberculosis a major health challenge. Globalization, health inequalities, competing economic interests and political instability contribute substantially to the spread of drug‐resistant strains, which are associated with high rates of morbidity and mortality. Of the 27 countries classified as high burden for MDR‐TB, 17 are in low‐income and middle‐income countries (LMICs). Shorter, all oral and less toxic multidrug combinations are required to improve treatment outcomes in these settings. Suitability for safe co‐administration with HIV drugs is also desirable. A range of strategies and several new drugs are currently undergoing advanced clinical evaluations to define their roles in achieving these aims. However, several clinical questions and logistical challenges need to be overcome before these new MDR‐TB treatments fulfil their potential. New policy issued by the World Health Organization (WHO) in 2016 to 2017 promotes novel treatment regimens. Scale‐up of such treatment options is needed to impact global success rates for drug‐resistant TB patients, especially in countries with large burdens.

## O123

### First‐hand experience from Brazil: lessons learned

**E Távora Dos Santos‐Filho**

Grupo Pela Vida‐RJ, Rio de Janeiro, Brazil

This presentation will discuss the following: the Brazilian National Programme for TB Control, the Brazilian Unified Health System, the new treatment guidelines 2011, new diagnostics available, the BRICS TB Research network, community engagement and community advisory boards, and the role of metropolitan councils. According to the World Health Organization's new ranking for TB, Brazil is in the 20th position among TB priorities (high burden country) for sensitive TB, and in 19th position for TB‐HIV coinfection with an incidence rate of 32.4/100,000 inhabitants or 67,000 new cases in 2016 (BRAZIL, 2017). The country is considered of low burden for MDR‐TB with 1.5% of all TB cases. TB is still the number one killer for those with AIDS in the country, as worldwide. Trend of incidence and prevalence is in a steady decrease since the 1990's and TB policies show clear improvements since early last decade. Efforts to take Brazil out of the high burden countries list has been a governmental priority. Yet, little has been achieved in cooperation between TB and HIV/AIDS programs: TB prophylaxis for people with HIV and aids is much below 10%. The efforts to expand DOTS coverage have also shown poor results before and after the Global Fund TB grant to Brazil (32.9% in 2007 to 35% in 2015) (ibid.). If the governmental TB policies have been inefficient to impact consistently the TB epidemic, a significant raise in public awareness on the epidemic and a relevant social mobilization in tuberculosis was observed. Efforts since 2002 to engage civil society and communities affected in the fight against TB: Forums of TB NGOs, as Metropolitan TB Councils and a Stop TB Partnership. This boosted cooperation.

## O124

### Safety and efficacy of dolutegravir‐based ART in TB/HIV co‐infected adults at Week 24

**K Dooley^1^, R Kaplan^2^, TN Mwelase^3^, B Grinsztejn^4^, E Ticona^5^, M Lacerda^6^, P Cahn^7^, E Belonosova^8^, M Ait‐Khaled^9^, K Angelis^10^, D Brown^11^, R Singh^12^, C Talarico^13^ and M Aboud^14^**

^1^Infectious Disease, Johns Hopkins University School of Medicine, Baltimore, MD, USA. ^2^Infectious Disease, Desmond Tutu HIV Foundation, Cape Town, South Africa. ^3^Clinical HIV Research Unit, Johannesburg, South Africa. ^4^Infectious Disease, Instituto de Pesquisa Clínica Evandro Chagas FIOCRUZ, Rio de Janeiro, Brazil. ^5^Tropical and Infectious Diseases, Hospital Dos de Mayo, Lima, Peru. ^6^Fiocruz/Tropical Medicine Foundation Dr Heitor, Manaus, Brazil. ^7^Fundación Huesped, Buenos Aires, Argentina. ^8^Regional Center For Prevention and Treatment of AIDS and Infectious Diseases, Moscow, Russian Federation. ^9^Clinical Development, ViiV Healthcare, Brentford, UK. ^10^Statistics, GlaxoSmithKline, Stockley Park, UK. ^11^Clinical Pharmacology, ViiV Healthcare, Abbotsford, Australia. ^12^Global Medical Dolutegravir, GlaxoSmithKline, Upper Merion, PA, USA. ^13^Clinical Development, ViiV Healthcare, Research Triangle Park, NC, USA. ^14^Global Medical Dolutegravir, ViiV Healthcare, Research Triangle Park, NC, USA

**Background**

Concurrent treatment of tuberculosis (TB) and HIV is compounded by drug interactions, overlapping toxicities and immune reconstitution inflammatory syndrome (IRIS). The efficacy and safety of dolutegravir (DTG) in antiretroviral treatment (ART) naïve adults with HIV/TB co‐infection was assessed.

**Materials and methods**

INSPIRING (NCT02178592) is a Phase 3b, non‐comparative, active control, randomized, open‐label study in HIV‐1 infected ART‐naïve adults (CD4 +  ³50 cells/µl) with drug‐sensitive TB. Participants on rifampin‐based TB treatment for up to 8 weeks were randomized (3:2) to receive DTG (50 mg twice daily during and for 2 weeks post‐TB therapy, followed by 50 mg once daily [OD]) or EFV (600 mg OD), with 2 investigator‐selected NRTIs for 52 weeks. For this Week 24 interim analysis, the proportion of subjects with plasma HIV‐1‐RNA <50 c/ml was derived using the FDA Snapshot algorithm in the intent to treat exposed (ITT‐E) population. Safety was assessed in all subjects who received study drug. An independent committee adjudicated IRIS episodes. The study was not powered to show a difference between study arms; no formal statistical hypothesis was tested.

**Results**

Of 113 subjects enrolled, 69 were randomized to DTG and 44 to EFV. Median baseline HIV‐1 RNA and CD4 +  cell counts were 5.10 log10 c/ml and 208 cells/µl in the DTG arm and 5.24 log10 c/ml and 202 cells/µl in the EFV arm; 40% were women. The proportions of subjects with HIV‐1‐RNA <50 c/ml at Week 24 were 56/69 (81%) (95% CI: 72%, 90%) in the DTG arm and 39/44 (89%) (95% CI: 79%, 98%) in the EFV arm. The lower DTG response rate was driven by non‐treatment related snapshot failures: five participants (7%) in DTG arm and none in EFV arm discontinued due to non‐treatment‐related reasons (loss to follow‐up/protocol deviations). Median CD4 +  cell increases at Week 24 were 146 cells/µL (IQR: 71, 214) for DTG and 93 cells/µl (IQR: 47, 178) for EFV. Two subjects discontinued study treatment due to AEs (both on EFV). TB‐Associated IRIS rates (adjudicated and investigator reported) were low (DTG, n = 4 [6%]; EFV, n = 4 [9%]). No subjects discontinued due to IRIS or liver events.

**Conclusions**

Interim week 24 results from this ongoing study show that DTG 50 mg twice daily appears to be effective and well‐tolerated in HIV/TB co‐infected adults receiving RIF‐based TB therapy. Rates of IRIS were low. There were no new toxicity signals for DTG and no discontinuations due to liver events. These data support the use of DTG based regimen in HIV/TB co‐infection.

## O13 – Women with HIV in Latin America

## O131

### Diagnosis and treatment of HIV‐positive women with comorbidities

**M Mantilla Suárez**

CEPAIN IPS, and Virrey Solís IPS, Bogotá, Colombia

Women represent more than half the number of people living with HIV worldwide. Young women between 10 and 24 years of age have twice the risk of HIV infection than men of the same age, and some contraceptive methods, such as medroxyprogesterone injections, have been associated with an increase of more than 40% in the risk of acquiring the virus. However, in our region, probably the biggest concern of women in this age range is not that of acquiring HIV but that of an unwanted pregnancy. The health topics relevant to women living with HIV differ according to age. For young women with HIV infection, contraception, pregnancy and HPV infection are the topics most frequently addressed during consultation. In older women, the topics are menopause, bone mineral disease, cardiovascular risk, neurocognitive disorders and cancer (associated or not with HIV/AIDS). Regarding mental health issues, we found that up to 82% of women present with depressive symptoms, anxiety and/or sleep disorders at some point throughout life. For the treating clinician of a woman living with HIV, it becomes a challenge to control the infection with antiretroviral therapy at each stage of the patient's life. This is because there may be multiple interactions between medical therapies such as contraceptives and hormone therapy with antiretrovirals, and also because of the effects that some antiretrovirals may have in skeletal, nervous and cardiovascular systems.

## O132

### ARV safety in pregnancy

**V Rouzier**

Integrated Care Center and Research Institution, Les Centres, GHESKIO, Port‐au‐Prince, Haiti

With the scale‐up of antiretroviral therapy (ART) for all HIV‐infected individuals, including pregnant women, from the time of HIV diagnosis to lifelong treatment, more and more women worldwide are being exposed to ART during childbearing years, conception, pregnancy and breastfeeding. The use of ART to prevent mother‐to‐child transmission of HIV has been one of the most successful public health interventions and as we move towards the elimination of maternal‐child transmission in many countries, many more women will either conceive on or initiate ART during pregnancy and their infants will be exposed to ART in utero and via breast milk. However, there is a paucity of data from randomized clinical trials on the safety of ART in pregnancy and breastfeeding. This presentation will review the current evidence for the safety of ART in pregnancy and on exposed infants. Strategies for the continued monitoring of old and new ART regimens in pregnancy and breastfeeding which are critical to inform guidelines and clinical practice will also be discussed.

## O133

### HIV‐infected pregnant adolescents are an extremely vulnerable population: a cohort study of PMTCT outcomes in Haiti

**MM Deschamps^1^, V Rouzier^2^, D Jannat‐Khah^3^, J Bonhomme^4^, J Pierrot^5^, L Reif^6^, JW Pape^1^ and M McNairy^6^**

^1^GHESKIO, Port au Prince, Haiti. ^2^Paediatrics, GHESKIO, Port au Prince, Haiti. ^3^General Internal Medicine, Weill Cornell Medicine, New York, NY, USA. ^4^Obstetrics, GHESKIO, Port au Prince, Haiti. ^5^Data Management, GHESKIO, Port au Prince, Haiti. ^6^Center for Global Health, Weill Cornell Medicine, New York, NY, USA

**Background**

To evaluate HIV+ pregnant adolescent outcomes as compared to youth and adults in the largest prevention of mother‐to‐child‐transmission (PMTCT) programme in Haiti.

**Materials and methods**

Retrospective data from HIV+ pregnant women and their infants enrolled in PMTCT care at GHESKIO from 1999 to 2014 were included. Adolescents included women age 15 to 19, youth were ages 20 to 24, and adults >24 years. Maternal outcomes include enrolment in PMTCT, receipt of antiretrovirals prior to delivery, and maternal retention through delivery. Infant outcomes include infant enrolment in PMTCT, HIV testing and HIV infection. Kaplan Meier methods were used to assess retention in PMTCT care.

**Results**

Among 4665 pregnancies, 7.8% (364) were adolescents, 15.8% (739) youth and 76.4% (3562) adults. Adolescents were more likely to be single as compared to adults (62% vs. 25%) and poorer (85% vs. 50% no income) (*p* < 0.001). Median CD4+ count among adolescents was 582 cells/mm^3^ vs. 524 and 476 for youth and adults respectively (*p* = 0.005). Among all pregnancies, 65% (235/364) of adolescents received antiretroviral medications prior to delivery as compared to 74% (547/739) youth and 76% (2719/3562) adults. Adolescents also were less likely to be retained in PMTCT care through delivery as compared to other age groups, 64% vs. 74% and 76% respectively. A total of 3,218 infants were reported born alive among 3414 women retained through delivery (94%), which did not differ by age group (92 to 95%). Among infants enrolled in PMTCT (total of 3218), 84% (183/217) of infants born to adolescent mothers completed HIV testing as compared to 89% (438/494) and 93% (2334/2507) of infants born to youth and adults. The HIV transmission rate ranged from 7.7% in adolescents (14/183), as compared to 5.3% youth (23/438) and adults 5.3% (124/2334). Retention of HIV+ women at 12 months after PMTCT enrolment was significantly lower in adolescents as compared to youth and adults: 72.7% (95% CI 67.6 to 77.2%), 80.4% (95% CI 77.2 to 83.2%) and 83.8% (95% CI 82.5 to 85.0%) respectively (log rank *p* = 0.0003).

**Conclusions**

Adolescent HIV+ pregnant women have poorer outcomes across the PMTCT care continuum compared to youth and adults. This is an extremely vulnerable population that needs tailored interventions within PMTCT clinics to improve uptake of ART and retention in care.

## O21 – HIV and Vulnerable Populations

## O211

### Transgender Persons

**B Braga**

Cuiabá, Brazil

This presentation analyses several aspects of Brazilian transmen and transmasculine people. As a Brazilian transman myself and an activist of the Brazilian Institute of Transmasculine people (IBRAT), I intend to contribute to debate about the relationship among gender relations, masculinities [1], education and healthcare in my home country. Therefore, as an example, among other things, I analysed written messages in on‐line diaries produced by transmen, social networks in general, as well as conversations in meetings, such as the First Brazilian National Transmen Conference (1 ENAHT). I/We observed in these narratives a tension between the medical knowledge‐power dichotomy, reiterating its truths about what is legitimate for the other, and a movement beyond this subjectifying relationship, revealing the potential of vlogs and/or social networks to create care relations between transmen and transmasculine transgendered Brazilian people. From the analysis of these relations, this presentation aims to produce knowledge in the healthcare field, primarily focused on professionals working in this area who are specialized in care services aimed at transsexuals, as well as in other networks of healthcare.

**Reference**

1. Female Masculinity, Jack Halberstam, Duke University Press, 1998

## O212

### Adolescents

**D Machado**

Universidade Federal de São Paulo (UNIFESP), São Paulo, Brazil

Adolescents are a critical population that is disproportionately impacted by the HIV epidemic. More than 2 million young people adolescents (10 to 19 years) are living with HIV today. Despite efforts to date, they continue to be extremely vulnerable, both socially and economically, to HIV infection. Adolescents with HIV include both those infected perinatally and behaviourally. Perinatally HIV‐infected adolescents are healthier than they were a decade or more ago, but they are significantly experienced with antiretroviral therapy, with increased virological resistance and other consequences of extended antiretroviral use. The presentation will discuss some of these challenges. The longer‐term impact of exposure to HIV and antiretroviral therapy throughout childhood are becoming apparent, with growing concern over neurocognitive, cardiovascular, renal and bone health. Also, they are at risk for multiple behavioural health risks that require consideration in prevention and healthcare programmes. Among the behavioural health risks, mental health problems are the most prevalent. Mental health problems are likely to interfere with adolescent ability to make decisions about negotiating situations of sexual possibility, or experimentation with substances. Difficulties with adherence to antiretroviral therapy are common during adolescence. Poor treatment knowledge and understanding of the benefits of taking combined antiretroviral therapy may impact adherence. Achieving and maintaining high levels of medication adherence are required to obtain the full benefits of antiretroviral therapy. HIV serostatus disclosure is also an immense challenge for adolescents living with HIV. There is a substantial need for interventions that respond adequately to all these healthcare needs.

## O22 – Hepatitis C. New Drugs and What Can We Learn from HIV?

## O221

### New HCV drugs in the region

**M Nelson**

Chelsea and Westminster Hospital, London, UK

The treatment of hepatitis C has been revolutionized by the advent of direct acting antiviral (DAA) drugs which have permitted combination regimens of highly effective, non‐toxic, easy to take agents without the need for interferon. High rates of success have been reported in previously believed difficult to treat populations and many countries are now discussing eradication strategies. Due to the high rates of success observed the pipeline of new agents for the treatment of hepatitis has slowed. Newer regimens aim to be pangenotypic with the possibility of shorter and hence potentially cheaper courses. Examples include Epclusa (sofosbuvir and velpatasvir) and Maviret (glecaprevir and pibentrasvir) the latter combination permitting pangenotypic treatment with 8 weeks of therapy. A triple combination of sofosbuvir‐velpatasvir‐voxilaprevir has also recently become available. This combination is pangenotypic and available for the treatment of non‐cirrhotic patients for 8 weeks and importantly for the small number who fail treatment for 12 weeks in those who have previously failed therapy with a DAA‐containing regimen. Recent data has also suggested encouraging results with DAAs for the treatment of acute hepatitis C which remains prevalent in men who have sex with men populations and studies are underway with these newer agents to potentially improve outcomes with shorter courses of therapy. DAAs where available are associated with high rates of response in all groups of individuals infected with hepatitis C and newer agents may permit with their very high rates of success and limited drawbacks newer strategies for the treatment of hepatitis C and hopefully eradication of the virus in the relatively near future

## O222

### Home/self‐testing: when, to whom? Experience from HIV

**O Sued**

Fundación Huésped, Buenos Aires, Argentina

In 2016 the World Health Organization (WHO) recommended that HIV self‐testing (HIV/ST) should be offered as an additional approach to HIV testing services. Currently, 43 countries have legal norms that allow the use of HIV/ST, while a further 46 countries reported they are working on implementing it. Rational to support HIV/ST is their potential for increased uptake and frequency of testing, in particular among key populations who may not otherwise be tested, thus contributing to the reduction on the gap for achieving the 90/90/90 targets. HIV/ST has the potential to be used with different approaches to maximize results. Evidence suggest that acceptability to HIV/ST is high, that the procedure identifies more HIV‐positive individuals as compared to standard testing services and that there is little if any evidence of psychological, medical or social harm. When implementing HIV/ST several themes need to be considered such as behavioural risk compensation, counselling, ability to perform, sensitivity and specificity, perceptions, instruction and supervision, and cost. Furthermore research on how to support linkage to confirmatory testing, prevention, treatment and care services is needed.

## O223

### Translating lessons from HIV to hepatitis C

**JL Santana**

University of Puerto Rico School of Medicine, San Juan, Puerto Rico

HIV management and therapy has been one of the major medical advances which has saved lives in the last 35 years. Hepatitis C (HCV) has recently become a worldwide public health concern due to its capability to increase all‐cause liver‐related morbidity and mortality as well as increasing health cost budgetary expenditures. There are many similarities between both viruses including the fact that anywhere from 20 to 60% of patients do not know their diagnosis. Both are life‐threatening blood‐borne viruses that can remain asymptomatic for many years thwarting awareness strategies and increasing the burden of community transmission. At present HCV being a curable disease, the global response is at a juncture where lessons learned from the HIV epidemic should form the template for a fast scaleable and sustainable response in order to curtail the epidemic and possibly eradicate or at least turn the disease into a rare incident manifesting illness. The applications of the lessons learned in strategies like seek, test, treat, retain and even treatment as prevention for selective populations are more than ever necessary as a comprehensive approach to create appropriate response from key policy stakeholders, pharmaceuticals, clinicians, patients and the community. All this implementation needs to be ascertained within a framework of respect, leadership, access to treatment, medication assisted programmes and social behaviour inclusion and expertise to seek and assist vulnerable and high risk groups like men who have sex with men, incarcerated individuals, homeless and people who inject drugs and substance use disorder.

## O224

### Risk factors for transmission of hepatitis C virus among the HIV population in Central Mexico: a case‐control study

**LE Ramírez‐Gonzalez^1^, A Piñeirua‐Menendez^2^, JF Sanchez‐Avila^3^, AH Hirata‐Hernandez^4^, A Camiro‐Zuñiga^1^, E Simental‐Aldaba^1^, I Zamora‐Tapia^1^ and J Sierra‐Madero^1^**

^1^Department of Infectious Diseases, Instituto Nacional de Ciencias Médicas y Nutrición, Salvador Zubirán, Mexico City, Mexico. ^2^HIV, Clinica Codnesa Iztapalapa, Mexico City, Mexico. ^3^Gastroenterology, Instituto Nacional de Ciencias Médicas y Nutrición, Salvador Zubirán, Mexico City, Mexico. ^4^Mental Health, Clinica Condesa Iztapalapa, Mexico City, Mexico

**Background**

Incidence of hepatitis C virus (HCV) infections among HIV‐infected patients has increased worldwide. Information about risk factors for HCV acquisition in HIV co‐infected patients is lacking in Latin America.

**Materials and methods**

Between 2016 and 2018 a case‐control study was prospectively conducted with patients attending three large HIV outpatient clinics in Mexico City. Cases were HIV‐positive male and female with HCV confirmed infection. HIV‐positive patients without HCV infection, matched for age and gender, served as controls. Written questionnaires were applied by direct interview, covering socio‐demographics, blood‐borne exposures, sexual behaviour and drug use. Univariable and multivariable regression analyses were used to identify factors independently associated with HCV co‐infection.

**Results**

From a population of 12,000 HIV infected patients, 326 participants were included (195 cases and 131 controls). Participants were mostly male (307 [94.1%]) with a median age of 37.3 years (interquartile range [IQR], 23 to 50.9). Only 6 participants (1.8%) had history of intravenous (IV) drug use. HCV infection was associated with body piercing (adjusted odds ratio [aOR], 2.98; 95% confidence interval [CI], (1.54 to 5.7), history of syphilis (aOR, 2.2; 95% CI, 1.1 to 4.2), mouth contact with seminal fluid (aOR, 2.06; 95% CI, 1.07 to 3.9), history of fisting (aOR, 4.6; 95% CI 1.6 to 13.3), and use of poppers during/before sex (aOR, 2.78; 95% CI 1.2 to 4.59). Self‐reported history of herpes genitalis was marginally related to HCV co‐infection (aOR, 4.7; 95% CI .99 to 22.3). Among exposures that were significant only at the univariable analysis in our study were, history of tattoo, unprotected receptive anal intercourse, group sex participation, using and sharing sex toys, rectal bleeding related to sexual intercourse. Other nosocomial exposures, acupuncture and major dental treatment were not related to HCV co‐infection.

**Conclusions**

HCV infection in HIV patients in Mexico is associated mostly with risky sexual behaviour among men who have sex with men. History of body‐piercing may be a route of transmission in our population. IV drug use is rarely a factor associated with HCV acquisition among HIV patients in central Mexico.

**References**

1. Van de Laar TJ, van der Bij AK, Prins M, Bruisten S. et al. Increase in HCV incidence among men who have sex with men in Amsterdam most likely caused by sexual transmission. J Infect Dis 2007;196:230‐8

2. Hernandez, M., Rivera Dommarco, J., Pablo Gutierrez, J.et. al. (2013). ENSANUT 2012. Analysis of its main results

3. Jin F, Prestage GP, Kippax SC et al. Prevalence and risk factors of hepatitis C in HIV‐negative homosexual men in Sydney, Australia. Aust N Z J Public Health 2005, 29: 536–539

4. Danta M, Brown D, Bhagani, et al. Recent epidemic of acute hepatitis C virus in HIV‐positive men who have sex with men inked to high‐risk sexual behaviours. AIDS 21: 983–991

## O23 – Submitted Abstract

## O231

### Switch to bictegravir/F/TA from DTG and ABC/3TC

**J‐M Molina^1^, D Ward^2^, HJ Stellbrink^3^, D Podzamczer^4^, C Brinson^5^, K Andreatta^6^, H Martin^6^, A Cheng^6^, M Mora^6^ and E Quirk^6^**

^1^Hopital Saint Louis, Paris, France. ^2^Dupont Circle Physicians, Washington, DC, USA. ^3^ICH Study Center, Hamburg, Germany. ^4^Hospital Universitari de Bellvitge, Barcelona, Spain. ^5^Central Texas Clinical Research, Austin, TX, USA. ^6^Gilead Sciences Inc, Foster City, CA, USA.

**Background**

Bictegravir, a novel, unboosted INSTI with a high barrier to resistance and low potential for drug interactions, has been co‐formulated with the recommended NRTI backbone of emtricitabine and tenofovir alafenamide (B/F/TAF) as a fixed‐dose combination (FDC). We report the primary Week (W) 48 efficacy and safety Phase 3 results of switching to B/F/TAF from dolutegravir plus abacavir/lamivudine (DTG+ABC/3TC) or FDC of DTG/ABC/3TC.

**Materials and methods**

HIV‐infected adults virologically suppressed on DTG/ABC/3TC or DTG plus ABC/3TC (DTG/ABC/3TC group), with estimated glomerular filtration rate (eGFR) ≥50 ml/min were randomized 1:1 to switch to B/F/TAF (50/200/25 mg) once daily or continue current regimen as DTG/ABC/3TC through week 48 in a double‐blinded fashion. Primary endpoint was proportion with HIV‐1 RNA ≥50 copies/ml (c/ml) at W48 (FDA snapshot). Non‐inferiority was assessed through 95.002% confidence intervals (CI) using a margin of 4%. Secondary endpoints were proportion with HIV‐1 RNA <50 copies/ml and safety (adverse events (AEs), laboratory results, bone mineral density (BMD) and renal biomarkers).

**Results**

Five hundred sixty‐three participants were randomized and treated (B/F/TAF n = 282, DTG/ABC/3TC n = 281): 11% women, 22% Black, median age 46 yrs (range 20 to 71). At W48, 1.1% switching to B/F/TAF and 0.4% continuing DTG/ABC/3TC had HIV‐1 RNA ≥50 c/ml (difference 0.7%; 95% CI ‐1.0% to 2.8%, *p* = 0.62), demonstrating noninferiority. At W48, proportion with HIV‐1 RNA <50 c/ml was 93.6% on B/F/TAF and 95.0% on DTG/ABC/3TC. No participant developed resistance to any study drug. The most common AEs were upper respiratory tract infection (10% B/F/TAF, 10% DTG/ABC/3TC), diarrhoea (9%, 5%), nasopharyngitis (7%, 8%) and headache (7%, 7%). Few participants (6 [2%], 2 [1%]) had AEs leading to premature study drug discontinuation. Mean BMD increased similarly in both groups. Percentage changes from baseline in renal biomarkers were similar between treatment groups (Table 1). Lipid parameters were similar between groups with the exception of a small decrease in triglycerides seen in the B/F/TAF group.

**Table:** Abstract O231–Table 1. Change from baseline at week 48

Change from baseline at Week 48	B/F/TAF (n = 284)	DTG/ABC/3TC (n = 283)	*p* value
Median % changes in Renal biomarkers, median			
Urine albumin: Creatinine ratio	+14%	+9%	0.74
Urine retinol binding protein: Creatinine ratio	+20%	+29%	0.31
Urine Beta‐2‐Microglobulin: Creatinine ratio	+21%	+17%	0.53
Median change in eGFR (ml/min)	+1.0	−1.8	<0.001
Mean % changes in BMD, mean			
Spine	+0.69	+0.42	0.33
Hip	+0.16	+0..30	0.47
Median change in Lipid parameters
Total cholesterol (mg/dL)	0	+2	0.77
LDL cholesterol (mg/dL)	+1	+2	0.42
HDL cholesterol (mg/dL)	−1	0	0.13
Total Cholesterol:HDL ratio	0.0	0.0	0.56
Triglycerides (mg/dL)	−5	+3	0.028

^ *p*‐values were from the two‐sided Wilcoxon rank sum test to compare the 2 treatment groups. * *p*‐values were from the ANOVA model including treatment as a fixed effect.

**Conclusions**

Switching to B/F/TAF was non‐inferior to continuing DTG/ABC/3TC with low rates of W48 virologic failure, high rates of maintained virologic suppression and no resistance. B/F/TAF was well tolerated, with a similar bone and urine protein safety profile to DTG/ABC/3TC.

## O24 – State‐of‐the‐ART

## O241

### Dual therapy

**P Cahn**

Fundación Huésped, Buenos Aires, Argentina

The rationale for dual therapy includes reducing antiviral exposure to make treatment safer without sacrificing virologic control and reducing cost. There have been several successful studies of a boosted PI plus lamivudine (3TC) or FTC that support the viability of a boosted PI, dual‐therapy strategy. In virologically suppressed patients, NEAT001/ANRS143 recruited treatment‐naïve patients, randomized to DRV/RTV plus either raltegravir (RAL) or FTC/TDF (N = 805). Although similar rates of virologic suppression were observed in the overall population, in patients with HIV‐1 RNA >100,000 copies/ml and CD4+ cell counts <200 cells/mm^3^, there was an excess risk of virologic failure for dual vs. triple therapy, and it was associated with resistance. The GARDEL study showed at 48 and 96 weeks non‐inferiority (in treatment‐naïve adults) of a dual regimen of lopinavir 400 mg/ ritonavir 100 mg plus lamivudine 150 mg, to that of a standard triple therapy of the same boosted PI and lamivudine or emtricitabine plus another NRTI. Non‐inferiority was maintained irrespective of baseline viral load and sensitivity analysis used to assess results. Similar results were reported in the ANDES study with Darunavir 800 mg/ritonavir 100 mg in fixed‐dose combination plus 3TC, compared to the same PI plus 2 NRTIS. Dual therapies based on lamivudine‐dolutegravir are being explored for initial treatment or as simplification of suppressed individuals: Recently the PADDLE study and ACTG5252 demonstrated that dolutegravir‐lamivudine resulted in potent antiviral activity. GEMINI trials are evaluating the non‐inferiority compared to a three drug regimen. The SWORD‐studies showed non‐inferiority in virologically suppressed patients receiving stable ART with no previous virologic failure when treated with dolutegravir plus rilpivirine dual therapy, compared to ongoing standard ongoing triple‐drug. So, dual therapy is emerging as an interesting option for treatment‐experienced, virologically suppressed patients and a potential alternative for treatment‐naïve patients.

## O242

### HIV and new drugs

**DR Kuritzkes**

Division of Infectious Diseases, Brigham and Women's Hospital, Harvard Medical School, Boston, MA, USA

The advent and global rollout of triple‐drug combination antiretroviral therapy (ART) for the treatment of HIV infection has resulted in a dramatic reduction in morbidity and mortality from this disease worldwide. To date, regimens that include a non‐nucleoside reverse transcriptase inhibitor (NNRTI), typically efavirenz (EFV) in combination with tenofovir disoproxil fumarate (TDF) and lamivudine (3TC) or emtricitabine (FTC) have been the preferred first‐line regimen. However, World Health Organization guidelines are shifting and the US President's Emergency Plan for AIDS Relief is pushing to replace EFV with the integrase strand‐transfer inhibitors (INSTIs) dolutegravir over the next 1 to 2 years. Newer drugs that have completed phase 3 trials and are awaiting regulatory approval include the NNRTI doravirine (DOR), and the INSTI bictegravir (BIC). Both drugs will be available single‐tablet formulations (DOR/3TC/TDF and BIC/FTC/TAF respectively) that do not require a boosting agent. A challenge to both of these newer regimens is the adverse drug‐drug interaction with rifampicin. Several injectable long‐acting formulations, including the combination of cabotegravir plus rilpivirine; the novel nucleoside reverse transcriptase translocation inhibitor MK8591, and broadly neutralizing antibodies (bNAbs) may permit administration of ART monthly, quarterly or even less frequently. Such advances may greatly simplify drug administration and increase adherence to ART for many patients.

## O25 – Submitted Abstract and CROI Update

## O251

### Transmitted drug resistance trends in individuals with recent infection in the Mesoamerican Region

**D Tapia‐Trejo^1^, S Ávila Ríos^1^, C García Morales^1^, R Pinzón Meza^2^, J Miguel Pascale^3^, G Porras Cortes^4^, C Quant Durán^5^, I Lorenzana^6^, RI Meza Martínez^7^, E Yolanda Palou^8^ and G Reyes Terán^1^**

^1^Center for Research in Infectious Diseases (CIENI), National Institute of Respiratory Diseases (INER), Mexico City, Mexico. ^2^Infectious Disease Clinic, Hospital Roosevelt, Guatemala City, Guatemala. ^3^Instituto Conmemorativo Gorgas de Estudios de la Salud, Panamá, Panama. ^4^Hospital Vivian Pellas, Managua, Nicaragua. ^5^Hospital Roberto Calderón, Managua, Nicaragua. ^6^Universidad Nacional Autónoma de Honduras, Tegucigalpa, Honduras. ^7^Laboratorio Nacional de VIH, Tegucigalpa, Honduras. ^8^Instituto Nacional Cardio‐Pulmonar, Tegucigalpa, Honduras

**Background**

Surveillance of transmitted drug resistance (TDR) in recently‐infected (RI) individuals is informative for HIV drug resistance (HIVDR) control policies. We longitudinally assessed TDR levels in RI individuals in five countries of the Mesoamerican region.

**Materials and methods**

Plasma samples collected by convenience sampling between July 2010 and December 2016 from recently diagnosed, antiretroviral treatment‐naïve HIV‐infected Guatemalan, Panamanian, Honduran, Nicaraguan, and Mexican individuals at reference HIV clinics, were screened to detect recent infection. RI individuals (seroconversion period window of 130 days, 95% CI 118 to 142) were identified as those with CD4+ T cell counts ≥200 cells/ml, plasma viral load >1000 copies/ml, less than one year of HIV diagnosis, and LAg‐Avidity test (Sedia) OD score <1.5 (confirmed by triplicate testing). TDR in RI individuals was assessed from available pol sequences using the Stanford HIVdb tool, considering viruses with a score >15 to efavirenz, nevirapine, any nucleoside reverse transcriptase inhibitor (NRTI), lopinavir, atazanavir, or darunavir as resistant.

**Results**

24.1% (418/1728) of persons in Guatemala, 28.8% (97/420) in Honduras, 29.2% (827/2829) in Mexico, 30.9% (100/323) in Nicaragua and 49.3% (182/369) in Panama showed evidence of RI. We did not observe significant differences in overall or drug class‐specific HIVDR levels between individuals with recent and long‐standing infection (LSI) in any country for the complete study period. No significant trends in overall or drug class‐specific TDR were observed along the study period in RI individuals, in any country. However, non‐nucleoside RT inhibitor (NNRTI) TDR in LSI individuals showed increasing trends in Mexico and Nicaragua (both *p* = 0.03). NNRTI resistance crossed the 10% threshold in RI individuals in Mexico, Honduras and Nicaragua after 2015. K103N was the most frequent surveillance DR mutation both in RI and LSI individuals in all countries. Mexico showed an increase in K103N frequency in RI in 2015 to 2016 (7.58%) vs. 2011 to 2012 (1.93%, *p* = 0.002).

**Conclusions**

Our results suggest increasing NNRTI HIVDR trends in some Mesoamerican countries. However, this increase was not specific to RI individuals and was also observed in individuals with LSI. More statistical power may be needed to find possible trends in TDR in RI individuals given the low proportion of the recently diagnosed population they represent in many Mesoamerican countries.

## O31 – Perspectives in Public Health: Old STIs – New Challenges

## O311

### Historic perspectives and public health significance of syphilis and other sexually transmitted infections

**MN Ghidinelli and MB Mello**

Pan American Health Organization (PAHO), Washington, DC, USA

The origins of syphilis are contended between two main hypotheses, the Columbian by which it was carried to Europe from the Americas by Columbus’ crewmen coinciding with the first reported outbreak; and the pre‐Columbian, indicating that venereal syphilis existed in Europe but was unrecognized. Four centuries later in 1905, Schaudinn and Hoffmann identified *Treponema pallidum* in chancres. Syphilis was, until the late 19th century, often confused with gonorrhoea, whose agent was isolated by Neisser in 1879. Gonorrhoea has been associated to biblical words and to descriptions dating back to 500 BC; it was first addressed as a public health problem in 1256 by Luis IX through his rulings on prostitution in Paris, and in 1611 by a decree of the British parliament. Spirochetes and gonococci, together with *Chlamydia trachomatis*, discovered by Prowazek in 1907, and the protozoan *Trichomonas vaginalis*, identified by Donné in 1836, are the causative agents of sexually transmitted infections for which a cure has been available since almost a century. However, according to the Latin American AMR Surveillance Network (ReLAVRA) high levels of gonococcal resistance to tetracycline, penicillin and ciprofloxacin has been emerging since 2005; and, more recently, extended‐spectrum cephalosporin‐resistant strains of Neisseria gonorrhoea were isolated. The World Health Organization (WHO) estimated in 2012 a global annual burden of 357 million incident cases of these four curable STIs: 143 due to *T. vaginalis*, 131 to *C. trachomatis*, 78 to *N. gonorrhoea* and 6 to *T. pallidum*. Of those, an estimated 64 million cases occurred in the Americas. The WHO Global Health Sector Strategy on STI (2016 to 2021) recommends prioritizing the implementation of demonstrated cost‐effective interventions, with the ultimate goal of eliminating selected STIs as a public health problem by 2030.

## O312

### Syphilis amongst key populations

**M Kamb**

Centers for Disease Control and Prevention (CDC), Atlanta, GA, USA

Syphilis, caused by the spirochete *Treponema pallidum,* results in the greatest STI‐associated morbidity and mortality outside HIV. Although mainly transmitted sexually, it also can be transmitted vertically during pregnancy. In the Americas, syphilis is endemic in most countries (typically <1% general population prevalence) but has high prevalence in certain key populations, such as sex workers (SW), men who have sex with men (MSM), transgender persons (TG) or migrant workers. Surveillance data are limited in Latin America & the Caribbean (LAC); however, available studies suggest rising syphilis case rates in the Americas. In Brazil, where MSM account for the majority of primary and secondary (P&S) (i.e. new) syphilis cases, case rates in pregnant women have increased each year since 2009. Similarly, in the United States most P&S syphilis occurs in MSM, but case rates in women and heterosexual men have increased each year since 2012. In Barbados, where syphilis was once well‐controlled, a continuing outbreak starting in 2011 has predominantly affected men (>70%), with almost half of all cases in HIV‐infected persons. A 2015 systematic review of syphilis seropositivity among key populations in LAC found prevalence in MSM was >7.5% in more than half of 35 studies, with highest prevalence in Andean and Southern Cone countries. In five studies in transgender populations, prevalence ranged from 6.5 to 43.3%. In 49 studies among female SWs, half found active syphilis in >5% of women. Syphilis continues to cause significant morbidity in LAC. Better surveillance could improve our understanding of syphilis trends and help support prevention activities.

## O313

### Innovation: community‐based STI clinic and interventions in San Francisco

**J Klausner**

Ronald Reagan UCLA Medical Center and Zuckerberg San Francisco General Hospital and Trauma Center, Los Angeles, CA, USA

In the 1990s and 2000s San Francisco had very high rates of new HIV infections and STDs in key populations such as men who have sex with men (MSM), sex workers and transgender persons. While there were primary care services available to all San Francisco residents, there were limited specialty services and no specific culturally‐competent services for MSM, sex workers and transgender persons. This presentation will review the steps taken to create sustainable services that are now nearly 20 years old for those at risk groups, as well as outcomes and various measures of impact. Attendees may model the San Francisco experience in increasing culturally‐competent and highly effective prevention and clinical sexual health services in their own settings.

## O314

### Outcomes of HIV and syphilis infection among pregnant women in Haiti

**MM Deschamps**

GHESKIO, Port au Prince, Haiti

**Objective**

To evaluate HIV+ pregnant women and infant outcomes, including syphilis testing, in the largest prevention of mother‐to‐child transmission (PMTCT) programme in Haiti.

**Methods**

Retrospective data from HIV+ pregnant women and their infants enrolled in PMTCT care at GHESKIO from 1999 to 2014 were included. Maternal outcomes include enrolment in PMTCT, receipt of antiretrovirals (ARV) prior to delivery, syphilis testing and treatment, maternal retention through delivery. Infant outcomes include infant enrolment in PMTCT, HIV testing and infection, Syphilis testing and infection. Four PMTCT programme periods were compared: period 1: mono ARV, period 2, dual ARV, period 3 Option B and period 4, Option B+. Kaplan Meier methods were used to assess retention in PMTCT care.

**Results**

Among 4665 pregnancies, median CD4+ was 494 cells/μl (IQR 328 to 691). At time of enrolment in PMTCT care, 3829 (82%) of pregnant women were tested for syphilis, with 8% of all women positive for syphilis (N = 395). A total of 3500 (75%) women received ARV prior to delivery and 73% (3414/4665) were retained in care through delivery with 22% lost prior to delivery, <1%) died, and (6%) stillbirths/abortions. 94% (3218) of infants born alive enrolled in PMTCT, of whom 2955 (92%) had complete HIV testing with 161 HIV+ infants for a 5.4% HIV transmission rate (9.8%, 4.6%, 5.8% and 3.6% in periods 1 to 4). Retention at 12 months was lower in period option B+ compare to other period. The proportion of women who tested positive for syphilis decreased from 16% (95/601) in period 1 to 8% (68/851) in period 4. Syphilis testing among infants increased from 17% to 91% across period 1 to 4 with 2 of 1682 infants being positive.

**Conclusion**

Despite dramatic reductions in MTCT in Haiti, interventions are needed to improve retention to achieve MTCT elimination of HIV and syphilis.

## O32 Update on PrEP

## O321

### Pre‐Exposure Prophylaxis: from clinical trials to implementation

**K Mayer**

Fenway Community Health Center, Boston, MA, USA

The efficacy of antiretrovirals for HIV prevention has been demonstrated in animal models and clinical trials of at risk heterosexuals, men who have sex with men, transgender women and people who inject drugs. Tenofovir disiproxil fumarate (TDF) co‐formulated with emtricitabine (FTC) taken daily has been approved by the World Health Organization and multiple national normative bodies for anti‐HIV pre‐exposure prophylaxis (PrEP). TDF/FTC PrEP has been found to be safe and well tolerated, though renal function needs to be monitored regularly, since reversible creatinine elevations can be seen in a small percentage of adherent PrEP users. TDF/FTC can also cause a clinically insignificant reversible decrease in bone mineral density, which may suggest additional monitoring is warranted for patients with pre‐existing osteoporosis or osteopenia. PrEP efficacy is directly related to medication adherence, with protective levels approaching 100% among those consistently using daily medication. However, several case reports of PrEP failures have been reported, most often in the setting of initiating PrEP when patients were acutely infected with HIV (i.e. pre‐antibody seroconversion). Transmission of multi‐drug resistant HIV has also been reported, and one PrEP failure in the setting of adequate protective drug levels has been reported in an individual with multiple daily intimate partners and recurrent rectal sexually transmitted infections (STI). Since PrEP does not protect against bacterial STI, users should be counselled to undergo routine screening and to consider using condoms if STI protection is desired. PrEP demonstration projects are underway in multiple countries in Latin America, but access remains limited for many who could benefit.

## O322

### HIV prevention: PrEP situation in Latin American countries

**G Ravasi**

Pan American Health Organization (PAHO), Washington, DC, USA

Current HIV prevention strategies have not been able to curb HIV incidence in Latin America and the Caribbean (LAC); the estimated number of new HIV cases remained stabilized since 2010 (120,000/year). New infections in adult males increased between 2010 and 2016, and young men (15 to 24) account for one‐third of new infections. Except for the Bahamas and Brazil, no country currently offers the complete range of HIV prevention interventions recommended by the World Health Organization (WHO). Nearly all countries provide free condoms to young people, men who have sex with men (MSM), female sex workers and transgender women, in most cases including lubricants. Nevertheless, condom use among MSM in their most recent sexual contact is 63%, highlighting the need for additional prevention tools such as pre‐exposure prophylaxis (PrEP). The regional PrEP target for LAC, established in 2015, is 10 pilot projects by 2020. By the end of 2017 the provision of PrEP to key populations in the public sector was still limited to the Bahamas and shortly in Brazil and Barbados. In addition, PrEP was available locally at very small scale through civil society organizations (e.g. Guatemala, Paraguay and the Dominican Republic) and private sector (e.g. Peru, Argentina and Chile). Limited knowledge and awareness in national programmes and civil society, resistance with respect to risk compensation and incidence of sexually transmitted infections, and budget gaps are common barriers. On the other hand, at least nine countries (Chile, the Dominican Republic, Guatemala, Haiti, Jamaica, Mexico, Paraguay, Peru and Colombia) are planning the implementation of demonstration projects in 2018 with various modalities and sources of financing. In addition, most countries have access to WHO prequalified low cost generic, including the PAHO Strategic Fund. The regional target of 10 projects will therefore soon be exceeded.

## O323

### Pre‐Exposure Prophylaxis of HIV in Mexico: adding colour to the prevention palette

**EH Vega‐Ramírez^1^, H López‐Gatell^2^, R Leyva‐Flores^2^, MA Cortés‐Ortiz^2^, S Diego Díaz^1^, P Espinosa‐Tamez^2^, MA Palet‐Sánchez^3^, G Vargas‐Guadarrama^1^, A Campos‐Loza^4^, F Badial‐Hernández^1^, C Magis‐Rodríguez^5^, J Sierra‐Madero^6^ and A González‐Rodríguez^1^**

^1^Center for the Prevention and Integral Care of HIV/AIDS, Mexico City, Mexico. ^2^National Institute of Public Health, Mexico City, Mexico. ^3^Ceda el Paso Comunicación Estratégica S.C., Mexico City, Mexico. ^4^HIV/AIDS Prevention Programme, Jalisco, Mexico. ^5^National Center for AIDS Prevention and Control (CENSIDA), Mexico City, Mexico. ^6^Instituto Nacional de Ciencias Médicas y Nutrición, Salvador Zubirán, Mexico City, Mexico.

Mexico has a concentrated HIV/AIDS epidemic. The estimated seroprevalence of HIV in men who have sex with men (MSM) is 17% (95% confidence interval: 16, 18%) [1] and 20% (15, 25%) in transgender women (TGW) [2]; 32% of HIV‐seropositive persons are unaware of their serostatus. Estimated HIV related mortality is 4.6 per 100,000 persons [3]. Free antiretroviral treatment is available at 78 public HIV/STD clinics since 2004, but late entry into care remains an important barrier to treatment success; 50% of HIV‐seropositive persons enter treatment with ≤200 CD4+ lymphocytes /µl [4], and 59% of them die within 6 months of treatment initiation [5]. Cost‐effective combination prevention programmes have increased awareness of HIV serostatus, HIV testing and linkage to care in MSM, but many opportunities still exist to improve the prevention of HIV in Mexico [6]. Pre‐exposure prophylaxis (PrEP), a relatively new component of combination prevention, has exhibit high efficacy to prevent HIV infection in diverse high risk populations and it has been proposed as a mechanism to stimulate HIV testing and counselling and to anticipate detection and linkage to care [7]. We propose a demonstration study to assess the feasibility and acceptability of including PrEP in HIV/STD combination prevention programmes in Mexico. This study is part of a tri‐national initiative led by the Evandro Chagas Institute for Infectious Diseases in Brazil. In Mexico, a multi‐site, prospective, cohort of 3000 HIV‐seronegative persons at high risk of HIV infection will be enrolled and followed between April 2018 to July 2019 at HIV/AIDS – STD clinics and through civil organizations in Mexico City and Jalisco. Participants will be ≥ 16 years of age and include MSM, TGW and heterosexual women whose partners are HIV‐seropositive. Eligible individuals will receive a daily oral dose of tenofovir disoproxil fumarate, 300 mg/emtricitabine, 200 mg. A concurrent communication intervention will aim to induce demand for PrEP in the target populations and clinicians. Formative research will assess barriers for health service utilization and opportunities for policy changes favourable to scaling‐up PrEP in Mexico.

**References:**

1. Is the HIV Epidemic Stable among MSM in Mexico? HIV Prevalence and Risk Behavior Results from a Nationally Representative Survey among Men Who Have Sex with Men. Bautista‐Arredondo, S, Colchero, MA, Romero, M, Conde‐Gonzalez, CJ & Sosa‐Rubi, SG. PLoS One 8, 6–13 (2013).

2. HIV prevalence, sociodemographic characteristics and sexual behaviors among transwomen in Mexico City. Colchero, MA et al. Salud Publica Mex. 57, 99–106 (2015).

3. Effect of Universal Access to Antiretroviral Therapy on HIV/AIDS Mortality in Mexico 1990‐2011. Hernández‐Ávila, JE et al. J. Acquir. Immune Defic. Syndr. 69, e100–e108 (2015).

4. CD4 counts at entry to HIV care in Mexico for patients under the ‘universal antiretroviral treatment program for the uninsured population,’ 2007‐2014. Hernández‐Romieu, AC et al. PLoS One 11, (2016).

5. AIDS‐related early mortality in Mexico between 2008 and 2012. Silverman‐Retana, O. et al. Salud Publica Mex. 57, s119–s126 (2015).

6. Impact and economic evaluations of a combination prevention programme for men who have sex with men in Mexico. Arantxa Colchero, M. et al. Aids 30, 293–300 (2016).

7. The current status of the use of oral medication to prevent HIV transmission. Mayer, KH & Ramjee, G. Curr. Opin. HIV AIDS 10, 226–232 (2015).

## Poster Abstracts

## Comorbidities and Complications of Disease and/or Treatment

## P001

### Dynamics of CD4 lymphocytes in patients infected by HIV with non‐Hodgkin lymphoma

**P Alatorre Fernández^1^ and P Volkow^2^**

^1^National Institute of Cancer, Mexico City, Mexico

**Background**

Kaposi's sarcoma (KS) is the most frequent AIDS‐defining malignancy. In Mexico, non‐Hodgkin lymphoma (NHL) and KS cause 25 and 14% of hospital deaths from AIDS. There is little information about the association of antiretroviral therapy (ART) with chemotherapy (CT). The cytotoxic effect of CT on immune recovery may be temporary or lasting. The aim of the study is to determine the dynamics of CD4 lymphocytes in patients with HIV and NHL KS or receiving CT.

**Materials and methods**

Cohort of HIV patients with KS or NHL treated at the Instituto Nacional de Cancerologia (INCan) from January 2008 to December 2012. Three groups: KS without CT, KS with CT and NHL with CT. We analysed demographic data, co‐infections, clinical stage, CT and number of cycles, viral load, CD4 and CD8 lymphocytes (before, during and after CT), immune recovery at follow‐up and mortality (Figures 1, 2).

**Results**

Seventy‐one patients: 40 with KS without CT, 13 KS with CT and 18 NHL with CT. All men. 45 (63%) had less than 200 CD4 at diagnosis. Patients received 2 to 12 cycles of CT. NHL patients had lower CD4 count previous to CT (*p* = 0.2021) during CT (*p* = 0.0007) and after CT (0.0033). Immune recovery at follow‐up did not differ between groups.

**Conclusions**

CT has a transient effect on immune recovery especially in patients with lymphoma so these patients should be closely monitored.

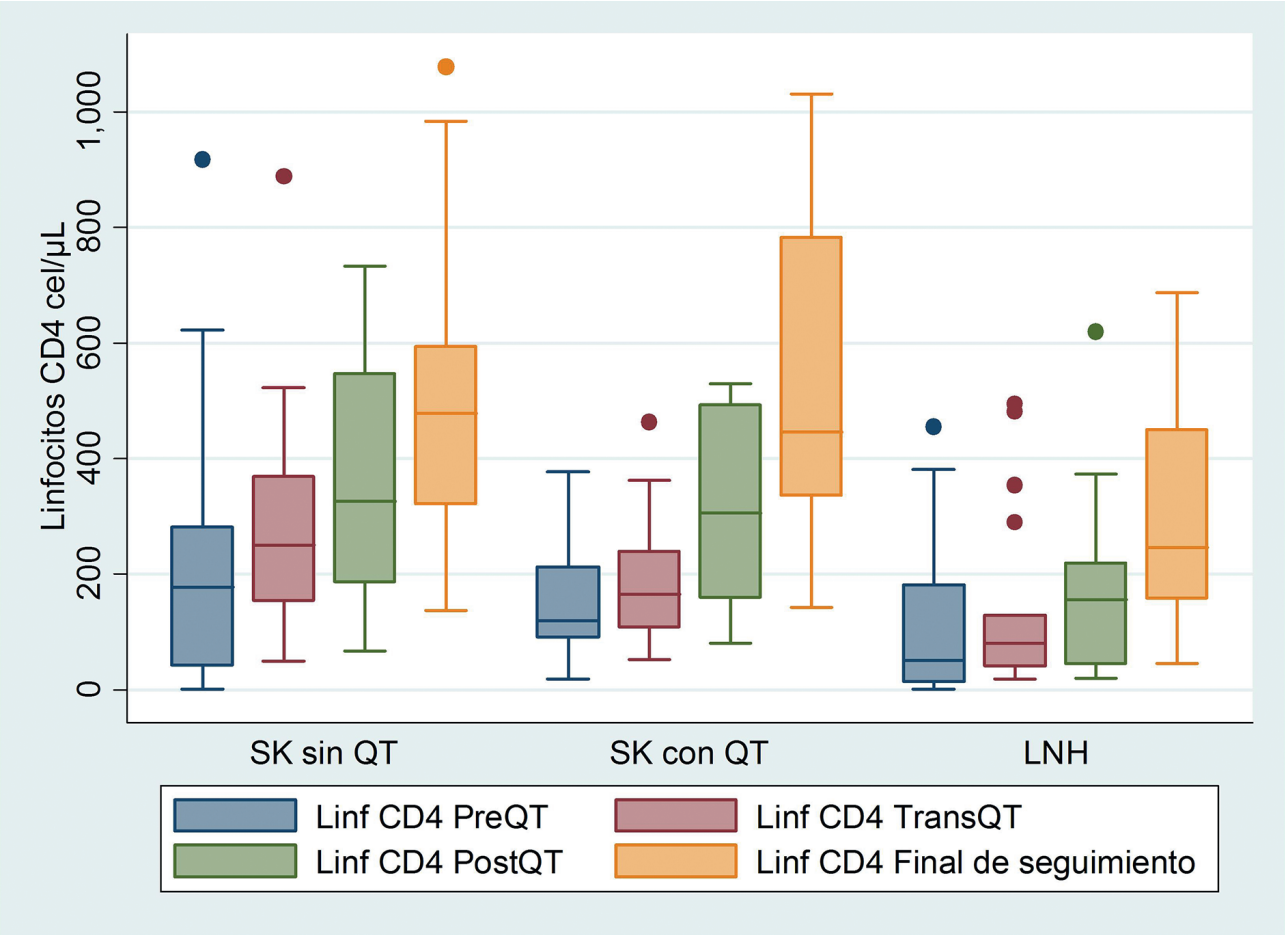

**Abstract P001‐Figure 1. Dynamics of CD4 Lymphocytes before (PreQT), during (TransQT) and after (PostQT) Chemotherapy and at the end of follow‐up (Final).**

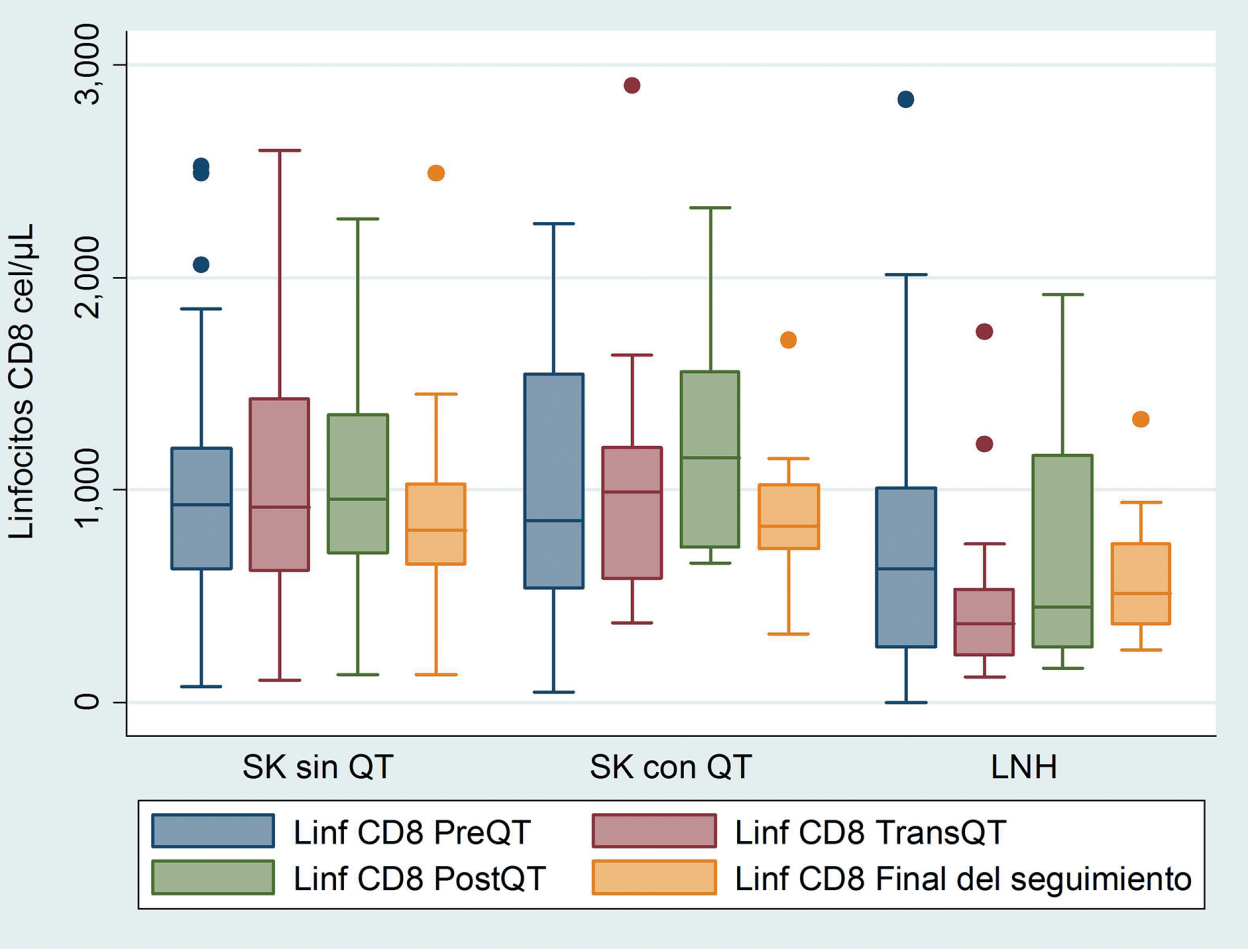

**Abstract P001‐Figure 2. Dynamics of CD8 Lymphocytes before (PreQT), during (TransQT) and after (PostQT) Chemotheraphy and at the end of follow‐up (Final).**

**References**

1. Antiretroviral Therapy Cohort Collaboration. Causes of death in HIV‐1‐infected patients treated with antiretroviral therapy, 1996–2006: collaborative analysis of 13 HIV cohort studies. Clin Infect Dis 2010; 50: 1387–96.

2. Mounier N, Spina M, Gabarre J, Raphael M, Rizzardini G,. AIDS related non‐Hodgkin lymphoma: final analysis of 485 patients treated with risk‐adapted intensive chemotherapy. Blood. 2006;107(10):3832‐3840.

3. Suneja G, Lin CC, Simard EP, Han X, Engels EA, Jemal A. Disparities in cancer treatment among patients infected with the human immunodeficiency virus. Cancer. 2016. Early view online. May 17.

4. Biggar RJ, Chaturvedi AK, Goedert JJ, Engels EA; HIV/AIDS Cancer Match Study. AIDS‐related cancer and severity of immunosuppression in persons with AIDS. J Natl Cancer Inst. 2007 Jun 20;99(12):962‐72.

5. Martín‐Carbonero L1, Palacios R, Valencia E, et al, Long‐term prognosis of HIV‐infected patients with Kaposi sarcoma treated with pegylated liposomal doxorubicin. Clin Infect Dis. 2008 Aug 1;47(3):410‐7.

## P002

### Renal function in perinatally HIV‐1‐infected patients

**R Al Hammoud, N Perez, G Rodriguez, L Benjamins, G Del Bianco, J Murphy and G Heresi**

Paediatrics, University of Texas, McGovern Medical School, Houston, TX, USA

**Background**

Renal dysfunction is a common morbidity in HIV‐infected patients. Mircroalbuminuria, a known early marker of HIV‐associated nephropathy, is associated with endothelial dysfunction in the adult HIV population. The objective of the current study is to determine for the interval 2007 to 2015 the frequency of microalbuminuria in a cohort of perinatally HIV‐1 infected individuals and test for relationships of renal affection with laboratory and clinical outcomes.

**Materials and methods**

Record review of perinatally‐infected HIV in an urban paediatric HIV clinic was performed. Data were obtained from patient records. A retrospective analysis was performed on a cohort of 69 perinatally HIV‐1 infected patients from October 2007 to June 2015.

**Results**

Sixty‐nine patients who had at least 2 microalbumin/creatinine ratio (MC) measurements separated by one month determined between 2007 and 2015 and for whom viral load (VL), CD4%, CD8%, CD8+CD38+%, CD8+HLA‐DR+ and complete demographic data were available were included. Thirteen patients (19%) met pre‐set definitions for microalbuminuria (>2 findings of MC >30 mg/g, separated by at least 1 month). Outcomes for the microalbuminuria group (13 patients) were compared to those of the 56 patients who did not meet the definition of microalbuminuria (Table 1).

**Table:** Abstract P002–Table 1. Demographics and Selected Laboratory Outcomes

Demographics and Selected Laboratory Findings	Non‐microalbuminuria	Microalbuminuria	*p* value
Number of patients, n (%)	56 (81)	13 (19)
Age, median (IQR) years	11.7 (6.8)	12.3 (9.62)	NS*
Gender(% female)	50	84	<0.01
Ethnicity (%) Black	71	69	NS*
Hispanic	19	30	NS*
MC, mg/g	5	51	<0.01
VL copies per ml, median	180	110	NS*
CD4%, median	32	32	NS*
CD8%, median	41	43	NS*
CD8CD38%, median	12	22	<0.01
CD8DR%, median	7	15	<0.01

*NS= Not significant

**Conclusions**

Approximately 19% of a small cohort of perinatally HIV‐1 infected children was found to have microalbuminuria. The microalbuminuria group exhibits marked activation of CD8+ but is not associated with the most common measures of HIV clinical status, CD4% and VL. This significant prevalence of renal affection reinforces the importance of screening for microalbuminuria in perinatally infected HIV children even with well controlled disease to allow timely intervention when needed in an attempt to delay/prevent cardiovascular disease in this patient population.

## P003

### Frequency and distribution of cardiometabolic comorbidities in clinically stable HIV patients on long‐term ARV therapy in Lima‐Callao, Peru

**J Hidalgo^1^, A Florez^2^, C Agurto^3^, Y Pinedo^4^, R Ayarza^5^, L Rodriguez^6^, A La Rosa^6^ and R Gutierrez^6^**

^1^Infectious Diseases, Via Libre ‐ Almenara Hospital, Lima, Peru. ^2^Infectious Diseases, Grau Hospital, Lima, Peru. ^3^Infectious Diseases, Sabogal Hospital, Infectious Diseases, Callao, Peru. ^4^ Infectious Diseases, Loayza Hospital, Lima, Peru. ^5^HIV Clinic, Via Libre, Lima, Peru. ^6^Medical Affairs, MSD Peru, Lima, Peru

**Background**

As access to HAART increases globally, the proportion of chronically treated, clinically stable HIV patients also grows. The aim of this study was to describe the differences in the presentation of the most common comorbidities observed in a population of clinically stable, successfully treated HIV infected adults in a country of limited resources.

**Materials and methods**

Review of medical records at 5 HIV clinics in Lima‐Callao, Peru, for HIV‐infected adults attending regular follow‐up visits in January or February 2016. Patients were adults (>21 years), ambulatory, on HIV therapy for >6 months and with no current or recent AIDS‐defining condition (within last 6 months). Records were reviewed to collect information regarding epidemiological, clinical and laboratory characteristics.

**Results**

Three hundred and five cases were identified. Patients were mostly male (73.1%), with a median age of 46.0 years, an average time from diagnosis of 9.41 years, and an average time on HAART of 7.78 years. Most patients were on an NNRTI‐based, first line regimen (76.4%). INSTIs were used in only 2.2%. Median CD4 count was 614.2 cells/µl and 90.8% (n = 277) had undetectable viral load. According to our observation, cardiometabolic comorbidities presented 3 patterns in our series: 1. Excess weight and obesity were highly frequent at 41.1, and 11.1% respectively. They appeared to be related to clinical stability and lifestyle of patients, rather than to age, gender, duration or type of ART; 2. Dyslipidaemia, hypertension and diabetes mellitus showed closer association to older age (Table 1) *indicates *p* ≤ 0.05 and longer duration of ART (with *p* value of 0.06 to 0.07, °); 3. Cardiovascular disease was observed in a low number of individuals (n = 10, 3.3%). Gender of patients and type of ART (NNRTI‐ vs. PI‐based) did not present differences for distribution of evaluated comorbidities (Table 1).

**Conclusions**

A population of stable, ambulatory HIV infected adults on long‐term HAART showed differences in the distribution of metabolic and cardiovascular comorbidities. Dyslipidaemia, diabetes mellitus and hypertension and were more frequent in older age and with longer duration of ART. Cardiovascular disease presented in low frequency in our population.

**Table:** Abstract P003–Table 1. Frequency and distribution of cardiometabolic comorbidities according to gender, age group, type and duration of ART

	Total	Men (n = 223)	Women (n = 82)	<50 years (n = 194)	>50 years (n = 111)	NNRTI‐based (n = 233)	PI‐based (n = 28)	<5 years (n = 89)	5 years (n = 216) ≥
Dyslipidaemia (n,%)	157, 51.5	111, 49.8	46, 56.1	89, 45.9*	68, 61.2*	122, 52.4	15, 55.6	39, 43.8°	111, 54.6°
Obesity (n, %)	34, 11.1	25, 11.2	9, 11.0	22, 11.3	12, 10.8	24, 10.3	3, 10.7	12, 13.5	22, 10.2
Excess weight (n, %)	127, 41.6	99, 44.4	28, 34.1	76, 39.2	51, 45.9	102, 43.8	12, 42.9	36, 40.5	91, 42.1
Diabetes mellitus (n, %)	22, 7.2	15, 6.7	7, 8.5	4, 2.0*	18, 16.2*	19, 8.2	1, 3.6	3, 3.4°	19, 8.8°
Hypertension (n, %)	27, 8.9	21, 9.4	6, 7.3	6, 3.1*	21, 19.9*	23, 9.9	0	4, 4.5°	23, 10.6°
Cardiovascular disease (n, %)	10, 3.3	6, 2.7	4, 4.9	4, 2.1	6, 5.4	6, 2.6	2, 7.1	1, 11	9, 4.2

## P004

### Renal insufficiency in HIV/AIDS patients: 5‐year experience in an intensive care unit

**V Chediack^1^, J Ricart^2^, M Sanchez Cunto^1^, C Dominguez^1^, G Mammoliti^1^, L Yesica^1^, M Nano^1^, L Gonzalez^1^, J Fernandez^1^, E Cunto^1^ and C Nogueras^3^**

^1^Intensive Care Unit, Infectious Hospital FJ Muñiz, Buenos Aires, Argentina. ^2^Infection, Infectious Hospital FJ Muñiz, Buenos Aires, Argentina. ^3^Directorate, Infectious Hospital FJ Muñiz, Buenos Aires, Argentina

**Background**

Renal involvement in HIV/AIDS patients, such as acute renal failure (ARF) and chronic kidney failure, is one of the most important age‐related non‐communicable diseases, even in the era of Highly Active Antiretroviral Therapy (HAART), and is independently associated with morbidity and mortality [1,2]. The aim of this is to describe clinical features, mortality and risk factors for Renal Disease (RD) of HIV/AIDS patients in an Intensive Care Unit (ICU), in a period of 5 years.

**Materials and methods**

This is a descriptive and observational study. Among 658 HIV/AIDS patients admitted in ICU from 2012 to 2017, 204 presented ARF on admission or during hospitalization. Their medical records were reviewed. Univariate analysis was performed to identify factors related to death. We performed descriptive statistics on percentage (%), median (Me), mean (M) and range. A *p* value of <0.05 was considered significant.

**Results**

The incidence was 31%, 72% were male, Me CD4 was 46 (0 to 1000) cells/ml and 82% had CD4≤200. 32% received HAART. Comorbidities identified were: enolism in 45%, smoking in 40%, previous kidney disease in 20%, high blood pressure in 10.2% and diabetes mellitus in 5.3%. Between risk factors we found: volume depletion in 88%, shock in 68%, sepsis in 61%, hypoalbuminemia in 67%, use of nephrotoxic drugs in 58% and liver failure in 17%. 85% presented ARF on admission. Acute kidney injury (AKI) was classified as stage I in 16%, as AKI stage II 25% and as AKI stage III 59%. M and Me proteinuria were 0.71 g/24 hours (0.5 to 4.39). Renal ultrasound was performed in 56% with hyper echogenic kidneys. 10% was treated with haemodialysis. Overall mortality was 54%, was higher in those who need haemodialysis (81%) than in those who do not require it (46%) with *p* = 0.004.

**Conclusions**

In our experience the incidence of renal failure is higher than in the literature, with male predominance [2]. The most important risk factor was volume depletion which is preventable and has accessible treatment [2]. In our cohort there was significant difference in mortality between those who need haemodialysis and those who do not. We encourage the early detection of comorbidities and risk factors for early treatment to reduce kidney impact.

**References**

1. Amanda Mocroft, Jens D. Lundgren , Michael Ross, et al. Development and Validation of a Risk Score for Chronic Kidney Disease in HIV Infection Using Prospective Cohort Data from the D:A:D Study.PLOS Medicine .https://doi.org/10.1371/journal.pmed.1001809 March 31, 2015 

2. Fakhrul M Islam, Jianyun Wu, James Jansson and David P Wilson. Relative risk of renal disease among people living with HIV: a systematic review and meta‐analysis.BMC Public Health 2012, 12:234.

## P005

### AIDS‐related *pneumocystis jiroveci* pneumonia in an intensive care unit: a descriptive study

**J Ricart^1^, M Sanchez Cunto^1^, V Chediack^2^, C Dominguez^2^, R Gregori Sabelli^2^, E Cortez^2^, J Fernandez^2^, P Velasquez Lopez^2^, S Caceres^2^ and E Cunto^2^**

^1^Infection, Infectious Hospital FJ Muñiz, Buenos Aires, Argentina. ^2^Intensive Care Unit, Infectious Hospital FJ Muñiz, Buenos Aires, Argentina

**Background**

HIV‐infected patients (HIP) who ignore their HIV status or those without viral suppression, may develop *Pneumocystis jiroveci* pneumonia (PJP) and require admission to an intensive care unit (ICU) [1], with a mortality rate of ICU HIV patients with PJP can still be up to 60%. Our aims are to describe clinical characteristics of patients with proven AIDS‐related PJP and determine factors related with mortality.

**Materials and methods**

Among 1122 HIP admitted to ICU of an Infectious Diseases Hospital between 2006 and 2016, 63 had PJP. Clinical features, radiological and laboratory investigations and outcomes were reviewed. Univariate analysis was performed to identify factors related to death. We performed descriptive statistics on percentage (%), median (Me), mean (M) and range. A *p* value of <0.05 was considered significant.

**Results**

Incidence was 0.056. The M age was 37 (14 to 76) years. 65% were male. 92% cases occurred with cell count CD4 ⁺ less than 100 cells/µl with a Me of 52. 90% did not receive highly active antiretroviral therapy. 75% presented weight loss >10% during the last 6 months, Karnofsky scale > 50 or hypoalbuminemia < 2.6 g/L. 70% presented diffuse bilateral interstitial infiltrates on chest X ray and 10% pneumothorax. 87% presented severe acute hypoxemic respiratory failure on admission, 40% required mechanical ventilation (MV). APACHE II (Acute Physiology and Chronic Health Evaluation II) score ≥13 points in 76% of the patients. Initial treatment was trimethoprim‐sulfamethoxazole (and corticosteroids) which should be suspended due to adverse events in 25% (15% granulocytopenia, 5% exanthema and 5% hyperkalaemia), it was replaced by intravenous pentamidine. Overall mortality was 43%. CD4+ <100 cells/µl, respiratory failure, MV and APACHE II ≥13 points was related to mortality (*p* ˂ 0.05)

**Conclusion**

In our serie MV, severity scores and severe immunosuppression were associated with mortality. Patients with CD4>100 cells survived. Malnutrition was related with mortality but p was not statistically significant. Consider initiate empirical PJP treatment in severe pneumonia in HIP.

**References**

1. Sadatomo Tasaka.Pneumocystis Pneumonia in Human Immunodeficiency Virus–infected Adults and Adolescents: Current Concepts and Future Directions. CliniCal MediCine insights: CirCulatory, respiratory and pulMonary MediCin e 2015:9(s1)

## P006

### Latin American prospective cohort of adult HIV‐positive patients (LATINA): factors associated with presenting symptoms at diagnosis

**G Rodriguez Loria^1^, L Mosqueda^2^, J Andrade Villanueva^3^, M Losso^4^, G Viloria^4^, L Hercilla^5^, M Mantilla Suárez^6^, G Lopardo^7^, M Michaan^8^, M Sanchez^9^ and W Belloso^9^**

^1^Research, CICAL, Buenos Aires, Argentina. ^2^Infectious Diseases, CAPASITS, Leon, Mexico. ^3^Infectious Diseases, Hospital Civil de Guadalajara, Guadalajara, Mexico. ^4^Immunocompromised Patients, Hospital General de Agudos Dr JM Ramos Mejía, Buenos Aires, Argentina. ^5^Infectious Diseases, Hospital Sabogal, Lima, Peru. ^6^Infectious Diseases, CEPAIN, Bogotá, Colombia. ^7^Infectious Diseases, FUNCEI, Buenos Aires, Argentina. ^8^Infectious Diseases, Hospital San Juan de Dios, La Plata, Argentina. ^9^Infectious Diseases, Hospital Italiano de Buenos Aires, Buenos Aires, Argentina

**Background**

Though HIV epidemics are not decreasing in most Latin American countries, there is little information about the baseline demographics. Clinical status at diagnosis and therapeutic course of patients initiating follow up. There is limited ongoing prospective data collection concerning status at diagnosis and clinical course of infection. Latina is an inception prospective cohort which started as of 1 January 2013.

**Materials and methods**

Latina Cohort is sponsored by CICAL (Coordinación para la Investigación Clínica en América Latina). Thirteen centres distributed in three countries are currently involved in the project: Argentina (9), Mexico (2), Peru (1) and Colombia (1). Inclusion criteria are: being at least 18 years old and having confirmed VIH diagnosis during year before inclusion. Data are recorded in a web platform. Recruitment started in Jan 1st 2013. The objective of the present analysis is to detect demographic and socio‐economic variables associated to patients presenting with symptoms at diagnosis. Logistic regression analysis was performed using SPSS v16Variables are presented with the pertaining summary measure and 95% confidence interval

**Results**

As of October 2017, 1866 patients were included in the cohort. Information on presence of symptoms at diagnosis was available in 1631 subjects (87.16%). Forty‐two percent (40 to 45) of these presented with symptoms at diagnosis. Sex, age, race and health‐care coverage system were complete in 100% of cases; level of education in 98.07% cases. Transmission mode in 89% of cases and CD4 in 90% of cases. The characteristics of the patient were: Male 82% (84 to 86), Latino race 57% (54 to 59), public healthcare coverage 79% (77 to 81); 23% (21.1 to 24.9) had complete primary education or less. Mean age was 32 years (31.16 to 32.74), median CD4 count at entry was 398 (203 to 574). Results found are presented (Table 1).

**Table:** Abstract P006–Table 1. Crude and Adjusted RR for demographic and socio‐economic variables. Multivariable analysis goodness of fit: ‐2 likelihood ratio: 1679.487

Variables Analysed	Univariate			Multivariate		
	Crude RR	95% CI	Significance	Adjusted RR	95% CI	Significance
Sex: Male	2.05	1.5 to 2.79	.000	2.13	1.51 to 3.00	.000
Public Health Care system	1.98	1.55 to 2.55	.000	1.94	1.48 to 2.55	.000
Primary education or less	1.27	1.03 to 1.56	.02	1.05	0.78 to 1.43	0.76
Age at diagnosis (effect per year)	1.02	1.01 to 1.03	.000	1.02	1.01 to 1.03	.000
CD4+ Count (Quartiles)						
‐ <204 (baseline)	1	NA	NA	NA	NA	
‐ 205‐398	0.17	0.13 to 0.24	.000	1.19	0.14 to 0.27	.000
‐ 399‐574	0.26	0.19 to 0.35	.000	0.28	0.21 to 0.39	.000
‐ Over 399	0.33	0.25 to 0.45	.000	0.36	0.26 to 0.49	.000
MSM	1.01	0.82 to 1.25	.92	NI	NI	NI
Latino	0.89	0.72 to 1.09	.24	NI	NI	NI

**Conclusions**

Results displayed show that almost half of the patients included are detected as HIV positive because of symptoms. This leads to worse prognosis and further spread of the epidemics. Early diagnosis is crucial to stop spread of the epidemics. This is a priority in the current context, where vaccines or cure are not to be expected soon. Strategies should include not only the routine HIV testing but also the active search in the community given the association with lower level of education and public healthcare coverage.

## P007

### Latin American prospective cohort of adult HIV‐positive patients (LATINA): 2017 baseline characteristics update

**G Rodriguez Loria^1^, L Mosqueda^2^, J Andrade Villanueva^3^, M Losso^4^, G Viloria^4^, L Hercilla^5^, M Mantilla Suárez^6^, G Lopardo^7^, M Michaan^8^, M Sanchez^9^ and W Belloso^9^**

^1^Research, CICAL, Buenos Aires, Argentina. ^2^Infectious Diseases, CAPASITS Leon, Leon, Mexico. ^3^Infectious Diseases, Hospital Civil de Guadalajara, Guadalajara, Mexico. ^4^Immunocompromised Patients, Hospital General de Agudos Dr JM Ramos Mejía, Buenos Aires, Argentina. ^5^Infectious Diseases, Hospital Sabogal, Lima, Peru. ^6^Infectious Diseases, CEPAIN, Bogotá, Colombia. ^7^Infectious Diseases, FUNCEI, Buenos Aires, Argentina. ^8^Infectious Diseases, Hospital San Juan de Dios, La Plata, Argentina. ^9^Infectious Diseases, Hospital Italiano de Buenos Aires, Infectious Diseases, Buenos Aires, Argentina

**Background**

Though HIV epidemics is sustained in most Latin American countries, there is little information about the baseline demographics, clinical status at diagnosis and outcomes in these patients. We present here the description of baseline characteristics of patients recruited in Latina inception cohort.

**Materials and methods**

This Cohort started collecting data in September 2013 Currently there are 13 sites distributed in: Argentina (9), Mexico (2) and Perú (1) and Colombia (1). We present basal information as of October 2017. Inclusion criteria are: HIV diagnosis within previous year and at least 2 prior visits to the site. Data are collected in an online “ad hoc” designed platform and was analysed including until October 2017. Patients are requested to have at least 2 visits per year. Thirteen sites in Argentina (9), Mexico (2), Perú (1) and Colombia (1) are participating. Numeric variables are summarized by means (95% CI) or medians (IQR) according to their statistical distribution. Categorical variables that allow inferential estimates are summarized by percentages and 95% CI (Table).

**Results**

Variables analysed had between 5 and 25% missing data. Overall 1866 patients were included in the analysis. Patient distribution per country is: México N = 1013, 54% patients, Argentina N = 570, 31%; Colombia N = 136, 7% and Peru N = 147 8%. Variables analysed had between 5 and 25% completion rate.

**Table:** Abstract P007–Table 1. Baseline characteristics of patients at cohort admission (N=1866)

Variables	Mean	IQR
Age	32.2	26.5 to 41.6
Time of formal education (years)	11	9.1 to 12.3
CD4+ Count	399	205 to 578
VL	78782	11,200 to 788,759
	**Percentage**	**95% CI**
Sex (male)	86.2	59.6 to 100
Public health care coverage	78	77.68 to 78.41
Sexual Transmission	88	86 to 90
Symptoms at the time of diagnosis	36	33.8 to 39.2
POD	20	18.1 to 21.1
Serious Non AIDS Conditions	2	2 to 3.8
Initiation of ARV	6	4.1 to 8.2

Centres from two new countries started participating in the cohort (Colombia and Peru) In comparison to data presented in 2016 there has been a 10‐point increase in public health coverage. The remainder variables have remained similar. The statistical difference between TAS and TAD between Mexican (111.07 to 67.22) patients and the rest of the cohort (113.26 to 71.67) remained significant, though in absolute terms it was reduced. Mean TAS/TAD difference were 2.19 and 4.5 respectively.

**Conclusions**

Demographic and transmission characteristics are similar to those reported in surveillance systems: patients are young, infected by sexual intercourse (mostly MSM), and male. The low rate of patients under ARV may be due to the fact that about one half of the patients (906) had been recruited previous to 2015 when WHO guidelines changed to recommend ARV treatment as early as possible. This deserves further exploration in order to assess acceptance of the recommendation amongst our population.

## P008

### Comorbidities in a sample of HIV‐positive adults in Colombia. Sub‐analysis of patients younger than 50 years

**P Martínez^1^, J Ruiz^2^, C Beltran^2^, M Rojas^2^, S Leon^2^ and W Lenis^3^**

^1^SIES Salud, Bogota, Colombia. ^2^Merck Sharp & Dohme, Bogota, Colombia. ^3^RECUPERAR SA, Bogota, Colombia

**Background**

According to UNAIDS  1], by 2015 in Colombia, 150.000 people lived with HIV, and the estimated prevalence in adults from 15 to 49 years old was 0.5%. The significant improvement in the survival of these patients with Antiretroviral Therapy (ART) has meant that currently they have an increase in the life expectancy that approaches the HIV‐negative population [2]. Furthermore, the HIV population younger than 50 years has shown an increase in comorbidities which is characteristic of older groups. As the presence of chronic comorbidities should be assessed in this population, we developed a study that was aimed at characterizing a group of adult patients with HIV in Colombia to determine the prevalence of comorbidities and risk factors. The following is a sub‐analysis of this study for the population younger than 50 years, where we aimed to characterize HIV diagnosed patients <50 years, attending two HIV care programmes and to describe the most frequent comorbidities among people <50 years with HIV diagnosis that attend two HIV healthcare programmes.

**Materials and methods**

This is a sub‐analysis of an observational, retrospective study. Medical records that met the inclusion criteria in two HIV care programmes were selected. Descriptive statistics were performed to summarize the clinical and demographic patient characteristics.

**Results**

From 669 medical records that were reviewed, 83,8%(561) were from patients <50 years (median age:30.7 years). Of these patients, 28.88% (162) were female, 20.86% (117) were overweight and 15.33% (86) were obese. Almost 70% had at least one comorbidity and 25.50% (140) received non‐HIV treatment medications. The most frequent comorbidities were syphilis 14% (81), dyslipidaemia 13.11%(73), lipodystrophy 5.21%(29), tuberculosis 4.13%(23) and chronic hepatitis B 3.77% (21). Regarding the risk factors for any comorbidity in this population, 27.83% (115) reported alcohol intake, 18.31% (102) tobacco history and 16.73% (93) drug abuse history.

**Conclusions**

This is one of the first studies to assess comorbidities of HIV patients in Colombia in the population <50 years. It is relevant that in this group of age, more than a half of the population have comorbidities that imply a potential reduction in their quality of life, despite improvements in survival resulting from the proper use of ART. HIV programs in Colombia should consider an integral management of patients <50 years to identify comorbidities and establish the adequate treatment.

**References**

1. ONUSIDA. http://www.unaids.org/es/regionscountries/countries/colombia

2. Marcus JL, Chao C, Leyden W, et al. Narrowing the gap in life expectancy for HIV+ compared with HIV‐ individuals. Conference on Retroviruses and Opportunistic Infections (CROI), February 22‐25, 2016, Boston. Abstract 54.

## P009

### Problems related to medication and clinical characteristics in patients older than 50 years with newly diagnosed HIV/AIDS.

**J Hoyos Pulgarin^1^, F Ramirez Briñez^2^, C Restrepo^1^, A Quiceno^3^ and J Alzate^4^**

^1^Infectious Diseases, Universidad Pontificia Bolivariana, Medellin, Colombia. ^2^Infectious Diseases, IPS Universitaria León XIII, Medellin, Colombia. ^3^General Medicine, IPS Universitaria León XIII, Medellin, Colombia. ^4^Epidemiology, Corporación para Investigaciones Biológicas, Medellin, Colombia

**Background**

Elderly patients with HIV/AIDS in Colombia is in upward trend, this being a population that has greater comorbidities and formulated drugs leading to an increased risk of medication‐related problems when combined with antiretroviral therapy [1,2]. The aim of this study is to describe the clinical characteristics and prevalence of medication related problems in elderly HIV/AIDS patients in Medellin, Colombia.

**Materials and methods**

We conducted a multicentre retrospective study including all elderly patients newly diagnosed with HIV/AIDS in three centres in Medellin, Colombia between January 2013 and December 2016, evaluating clinical and laboratory characteristics, Charlson comorbidity index and medication‐related problems (drug‐drug interactions, polypharmacy, potentially inappropriate medication and anticholinergic load).

**Results**

We included 240 patients of which 196 (81.67%) were male, median of age 54 years (IQR 52 to 59 years), most were in stage three (130, 54.17%) and 97 patients (40.42%) had some defining AIDS condition, the most frequent being wasting syndrome (51, 21.25%) and pulmonary and extra pulmonary tuberculosis (33, 13.75%). The median CD4 cell count was 226 (IQR 84 to 418) and viral load 77,888 copies/ml (IQR 20608 to 242000) at the time of diagnosis. Non‐infectious comorbidities occurred in 144 patients (60%), the most common being arterial hypertension (61, 25.42%) followed by dyslipidaemia (44, 18.33%). The median of the Charlson comorbidity index was 7 (IQR 1 to 7). The 79.47% receive antiretroviral treatment based on NNRTI (EFV) being the most frequently formulated treatment TDF/FTC/EFV (41.48%). The problems related to medication were found in 63.75% of the population, drug interactions 60.42%, polypharmacy with ARV 52.08% and without ARV 20.83%, potentially inappropriate medication 9.58% and anticholinergic risk score ≥3 2.92%. Patients ≥60 years had a higher frequency of problems related to medication compared to those under 60, but this difference was not statistically significant (73.54% vs. 60.96%, *p* = 0.092). When comparing the characteristics evaluated between patients under and over 60 years, statistically significant differences were found in the percentage of AIDS defining disease (*p* = 0.007) and the presence of non‐infectious comorbidities (*p* = 0.01), with a higher frequency in the group of patients with an age ≥60 years.

**Conclusions**

The elderly population with HIV/AIDS in Medellin, Colombia is diagnosed in advanced stages of their disease and with a high burden of non‐infectious comorbidities that may be involved in the high percentage of problems related to the medication found in this study. The impact that this can have on important outcomes must be sought.

**References**

1. Guaraldi G, Falutz J, Mussi C, Silva AR, editors. Managing the older adult patient with HIV [Internet]. Cham: Springer International Publishing; 2016 [cited 2016 Oct 29]. Available from: http://link.springer.com/10.1007/978‐3‐319‐20131‐3,

2. Schouten J, Wit FW, Stolte IG, Kootstra NA, van der Valk M, Geerlings SE, et al. Cross‐sectional comparison of the prevalence of age‐associated comorbidities and their risk factors between HIV‐Infected and uninfected individuals: The AGEhIV Cohort Study. Clin Infect Dis. 2014 Dec 15;59(12):1787–97.

## P010

### Cardiovascular risk in elderly patients with HIV. Comparison of Framingham versus D:A:D cardiovascular risk calculators

**M López Hernandez**

Hospital General Regional N°1 IMSS, CLISIDA, Mexico City, Mexico

**Background**

The estimation of cardiovascular risk is the most reasonable way and determine priorities for cardiovascular prevention in asymptomatic persons and allows cost‐effective allocation of resources depending on the needs, defined as the risk of cardiovascular disease. Patients with HIV infection have an increased risk of developing cardiovascular disease due to complex interactions between traditional risk factors, antiretroviral treatment and HIV infection

**Materials and methods**

From all the patients with chronic HIV infection on antiretroviral treatment of HIV clinic of the Hospital General ISSSTE Tacuba, we included those older than 30 years. Age, gender and proportion were determined of patients with viral load below 40 copies / mL, count CD4 + T lymphocytes, cholesterol, LDL fraction, HDL, presence diabetes, hypertension, smoking, treatment naïve, cardiovascular risk scales Framingham and D:A:D, and compared between two groups of patients

**Results**

One hundred and eight patients were included, 50 younger than 50 years and 58 over 50 years old, with a ratio of male patients 96 vs. 82.75% (*p* = 0.69) respectively, no significant difference was found between both groups of patients. The proportion of patients with viral load under the detection limit (<40 copies/ml) was 88% vs. 93.1% (*p* = 0.27), CD4 + lymphocyte 602.8 vs. 498 cells / mm^3^ (*p* = 0.14), total cholesterol levels vs. 188.1 198.9 (*p* = 0.43), fractions LDL vs. 106.1 108.4 mg/dl (*p* = 0.58) and HDL 43 vs. 45 mg/dl (*p* = 0.78). As cardiovascular risk scores were observed a higher score in the case of the Framingham in patients over 50 years with an average of 16.87% of risk ten years against 5.32% in less than 50 years (*p* < 0.001) in the score the D:A:D for cardiovascular risk five years in the group of more than 50 years was 5.42% vs. 2.76% (*p* < 0.001)

**Conclusions**

In patients older than 50 years old, the cardiovascular risk estimated by both the Framingham and D:A:D calculators is increased significantly with respect to patients under this age. No significant differences were observed in lipid profiles in both groups, but a higher incidence of diabetes and hypertension was observed in the group of patients over 50 years old. Large cohort studies are required to follow long term to estimate which of these two models fits better for Mexican patients living with HIV infection.

**References**

1. Triant V. HIV infection and coronary heart disease: an intersection of epidemics. J Infect Dis. 2012; 205(Suppl 3):S355‐61.

2. Anderson KM, Odell PM, Wilson PW, Kannel WB. Cardiovascular disease risk profiles. Am Heart J. 1991;121:293‐8

3. Kaul S, Fishbein MC, Siegel FJ. Cardiac manifestations of acquired immune deficiency syndrome. Am Heart J 1991; 122: 535‐44.

4. Triant VA, Lee H, Hadigan C, Grinspoon SK. Increased acute myocardial infarction rates and cardiovascular risk factors among patients with human immunodeficiency virus disease. J Clin Endocrinol Metab 2007; 92: 2506‐12.

## HIV/Hepatitis Co‐infection

## P011

### Real‐world effectiveness of ledipasvir/sofosbuvir (LDV/SOF) for 8 weeks in patients co‐infected with HCV and HIV‐1

**P Buggisch^1^, A Moreno^2^, V Isakov^3^, L Backus^4^, D Ain^5^, J Gonzalez‐Garcia^6^, S Naik^7^, S Mehta^7^, J Lee^7^, M Mertens^7^, J Llewellyn^7^, M Natha^7^, K Kersey^7^, A Osinusi^7^, J Slim^8^, K Zhdanov^9^, J Berenguer^10^, S Zeuzem^11^ and J Mendez‐Navarro^12^**

^1^Ifi‐Institute for Interdisciplinary Medicine, Hamburg, Germany. ^2^Infectious Diseases, Hospital Ramon y Cajal, Madrid, Spain. ^3^Institute of Nutrition, Russian Academy of Medical Sciences, Moscow, Russian Federation. ^4^Health Care System, Veterans Affairs Palo Alto, Palo Alto, USA. ^5^Ruane Medical and Liver Health Institute, Los Angeles, CA USA. ^6^IdiPaz Internal Medicine, Hospital Universitario La Paz, Madrid, Spain. ^7^Gilead Sciences Inc, Foster City, CA USA. ^8^Saint Michaels Medical Center, Newark, NJ, USA. ^9^Military Medical Academy, St Petersburg, Russian Federation. ^10^IiSGM Infectious Diseases, Hospital General Universitario Gregorio Maranon, Madrid, Spain. ^1^ Medical Center, Johann Wolfgang Goethe University, Frankfurt, Germany. ^12^Public Health and Medical Affairs (PHMA), Gilead Sciences, Mexico City, Mexico

**Background**

Real world cohorts (RWC) have demonstrated excellent efficacy of LDV/SOF for 8 weeks in HCV monoinfected patients. Real world effectiveness data of LDV/SOF for 8 weeks in HIV/HCV co‐infection is emerging from several multiple single‐centre and multicentre prospective and retrospective cohorts. The aim of this study was to describe the effectiveness of the single tablet regimen of LDV/SOF for 8 weeks in HCV genotype (GT) 1 patients with HIV/HCV co‐infection in RWC.

**Materials and methods**

In this descriptive analysis, data from two prospective studies, one investigator sponsored and one registrational trial, one prospective RWC, three retrospective RWC of LDV/SOF for 8 weeks in HIV/HCV co‐infected patients were compared. RWC were selected based on willingness to participate and had at least 15 HIV/HCV co‐infected patients. The prospective trials include data from Ain et al (investigator sponsored) and Isakov et al (registrational trial). The RWC include the Deutsches Hepatitis C‐Register, Madrid Coinfection Registry (Madrid‐CoRe),Veterans Affairs HCV Registry, and Slim et al, representing diverse patient populations from Europe and US. Baseline characteristics and efficacy were analysed.

**Results**

The majority of the 294 patients included in this descriptive analysis were GT 1, treatment naïve (TN), non‐cirrhotic (NC) and had a HCV viral load < 6 million. The prospective cohorts enrolled 79 patients with the following baseline characteristics: mean age (43 years), male (66%), white (89%) and GT 1a (41%). The RWC studies assessed enrolled 215 patients with the following overall baseline characteristics: mean age (54 years) male (84%), white (82%) and GT 1a (75%) in those that reported demographics. The overall SVR12 from six diverse real world and post‐marketing cohorts was 94% (277/294). The individual study results are presented in Table 1.

**Conclusion**

This analysis of diverse cohorts from the EU and US yielded high SVR rates similar to SVR rates seen in multiple RW monoinfected cohorts and supports the use of 8 weeks of LDV/SOF in TN, NC GT 1 HIV/HCV co‐infected patients with a baseline HCV viral load < 6 million.

**Table:** Abstract P011–Table 1. SVR12 results

Study	Study Design	SVR12, % (n/N)
Ain et al.	Prospective LDV/SOF x 8 weeks	90 (18/20)
Russian cohort (Isakov et al.)	Prospective LDV/SOF x 8 weeks	97 (57/59)
VA	Retrospective LDV/SOF x 8 weeks	97 (30/31)
DHC‐R	Retrospective LDV/SOF x 8 weeks	96 (73/76)
Madrid‐CoRe	Retrospective LDV/SOF x 8 weeks	91 (85/93)

## P012

### HCV treatment in HIV/HCV co‐infection in Brazil, 2016–2017

**S Monzani Vivaldini^1^, M Santos^1^, E Carlos Almeida^1^, F Alvarenga Pinto^1^, J Neri Gomes^1^, N Fernandes^1^, E Argia Basile Cattapan^1^, M Jacintho Mendes Correa^2^, F Moreira Rick^1^, G Mendes Pereira^1^, K Tonini^3^, A Oliveira Bernard^4^ and A Schwartz Benzaken^1^**

^1^Ministry of Health, Departament IST/AIDS and Viral Hepatitis, Brasília, Brazil. ^2^Clinical Hospital of São Paulo, University of São Paulo, São Paulo, Brazil. ^3^Department of Pharmaceutical, Ministry of Health, Brasília, Brazil. ^4^Department of Pharmaceutical Assistance, Ministry of Health, Brasília, Brazil

**Background**

From 2007 to 2016, there were about 15,000 people identified with HIV and hepatitis C virus (HCV) co‐infection in Brazil. Treatments for HIV and HCV are universal and provided free of charge by the Ministry of Health of Brazil (MoH). Since 2015, MoH has been offering direct‐acting antivirals (DAAs) for HCV treatment – which means a better option, especially for HIV/HCV co‐infected people, once therapy presents less drug interactions with antiretrovirals and in Brazil HIV/HCV co‐infection became priority for HCV treatment regardless liver fibrosis. The aim of this study is to describe the characteristics of HIV/HCV co‐infected people treated for HCV in Brazil.

**Material and methods**

We analysed data collected from Drug Dispensation Information System regarding HCV treatment of all HIV/HCV co‐infected people in public healthcare in Brazil from 2016 to 2017.

**Results**

A total of 2,538 HIV/HCV co‐infected people were identified. Among them 82% (2071) were genotype 1; 1% (35) genotype 2; 15% (370) genotype 3; 2% (62) genotype 4. Concerning therapeutic scheme, 88% (2242) were 12 weeks of sofosbuvir and daclatasvir; 9% (234) 24 weeks of sofosbuvir and daclatasvir; 3% (62) other treatments (sofosbuvir and simeprevir; sofosbuvir and ribavirin; daclatasvir, peginterferon and ribavirin. The south and southeast regions distributed the majority of treatments, around 86% (2201). From them, the state of São Paulo (southeast) was responsible for the highest number of treatments of HIV/HCV co‐infected patients, followed by Rio Grande do Sul (south), Santa Catarina (south) and Minas Gerais (southeast).

**Conclusions**

The strategy of prioritizing HCV treatment for HIV/HCV co‐infected people, considering morbidity and mortality of co‐infection, plays an important role on HIV/HCV control – being crucial for public health response. As Brazil increases access to treatment in this population, this strategy could be implemented by other low‐middle income countries. Knowing that new interferon‐free therapy has more than 90% of efficacy, the next step is to evaluate the sustained virologic response in HIV/HCV co‐infected people in order to offer the most suitable therapy for HIV/HCV co‐infection in the country.

## P013

### Investigation of the increasing number of hepatitis A cases among men in the biggest city in Latin America in 2017

**M Jorge de Queiroz, F Alvarenga Pinto, R Abrahão Ribeiro, S Monzani Vivaldini, R de Almeida Coelho and G Mendes Pereira**

Ministry of Health of Brazil, Department of STI, HIV/AIDS and Viral Hepatitis, Brasília, Brazil

Several outbreaks of hepatitis A were reported since June of 2016 in 20 European Union countries (Spain, United Kingdom, Italy, Germany, Portugal and others), with over 2800 cases among men who have sex with men (MSM) [1]. In 2017, 970 cases of hepatitis A were reported in the state of São Paulo/Brazil (534% increase comparing to the previous year). In immunocompetent individuals, hepatitis A infection has self‐limited viremia and duration. In immunocompromised individuals, the viremia tends to be longer, the viral load tends to be higher, and they may present a greater potential risk of transmission, even with the cessation of clinical presentation [2]. In Brazil, the hepatitis A vaccine is available for children under the age of 5 years and for specific populations, including people living with HIV(PLHIV). Our study aimed to assess the profile of the reported cases and the prevalence of HIV among them. A retrospective observational study was carried out by analysing information from the database of individual notification of hepatitis A cases registered in the National Disease Notification System (SINAN) in the Brazilian State of São Paulo in 2017. We analysed information variables related to gender, age, race, educational level, municipality of residence, suspected source of infection and co‐infection with HIV. From the 970 cases reported, 81.5% were men (n = 791). Among them, 74.9% were residents of the capital, 72.3% were between 20 to 39 years old (n = 572), 50.3% (n = 400) white, 50% concluded high school or higher degrees and 16.1% (n = 128) were infected with HIV. Among HIV+ men, 62.5% were white, 51% had concluded high school or higher degrees. 66.1% of the reports did not provide information on the possible source of infection (n = 523); among the notifications that correctly registered this information field, 42% corresponded to the sexual route. The municipalities of São Paulo are among the best‐evaluated cities in the national ranking of sanitation, which makes unlikely the association of the sudden increase of cases to water or food contamination. The occurrence of the events coincided with the internationally reported outbreaks, and the individual characteristics of the reported cases in São Paulo strongly suggests the event refers to an outbreak related to sexual transmission between gays and other MSM. The perceived high HIV prevalence reinforces the need for warranting immunization for hepatitis A among PLHIV, and provides strong points of argument to the idea of its expansion to MSM and other vulnerable groups.

**References**

1. Werber D, Michaelis K, Hausner M, Sissolak D, Wenzel J, Bitzegeio J, et al. Ongoing outbreaks of hepatitis A among men who have sex with men (MSM), Berlin, November 2016 to January 2017 – linked to other German cities and European countries. Euro Surveill [Internet] 22(5).

2. Regan DG, Wood JG, Benevent C, Ali H, Smith LW, Robertson PW, et al. Estimating the critical immunity threshold for preventing hepatitis A outbreaks in men who have sex with men. Epidemiology & Infection. 2016 May;144(7):1528–37.

## P014

### Change of evaluation and treatment patterns over a 4‐year period of direct‐acting antiviral use in HIV/HCV co‐infected patients

**J Gonzales Zamora and Z Henry**

University of Miami. Miller School of Medicine, Medicine, Miami, FL, USA

**Background**

Since the advent of new Direct Acting Antivirals (DAA)**,** substantial changes in hepatitis C (HCV) treatment guidelines have occurred. However, little is known about how these recommendations have been adopted into clinical practice. Our aim was to determine the changes in HCV evaluation and treatment patterns in our practice over a 4‐year period of DAA use in HIV/HCV co‐infected patients.

**Materials and methods**

We conducted a retrospective review of the HIV/HCV co‐infected patients treated with DAA in the outpatient clinic of Jackson Memorial Hospital in Miami, USA from January 2014 to December 2017. Data were divided into two periods: period 1 (2014 to 2015) and period 2 (2016 to 2017). We compared demographic and clinical variables by treatment period. Data was analysed in SPSS 22, New York, USA.

**Results**

There were 78 co‐infected patients treated with DAA. The male to female ratio was 2:1 with a mean age of 55.6 (SD ±7.88) years. Most patients were African American (57.7%). Antiretroviral therapy (ART) was received by 96.2% of patients. The mean CD4 count was 637 cells/uL (SD ±334.35). Genotype 1a was the most frequent HCV genotype (61%). The mean baseline HCV log10 IU/ml was 6.18 (SD ±0.76). There were 37 (47.4%) patients in period 1 (2014 to 2015) and 41 (52.6%) patients in period 2 (2016 to 2017). In comparison with the rest of the study cohort, patients in period 2 had a lower proportion of advanced liver disease (24.4% vs 48.6%, *p *= 0.026). They underwent more elastography (34.1% vs. 2.7%, *p* < 0.001) and less ultrasound (78.0% vs. 97.3%, *p* = 0.011). They were more often treated with ledipasvir/sofosbuvir (85.4% vs. 56.8%, *p* = 0.005) and less frequently treated with simeprevir/sofosbuvir (0% vs. 32.4%, *p* < 0.001). Gastrointestinal side effects from DAA were reported less frequently in period 2 (2.4% vs. 18.9%, *p* = 0.017). In terms of ART, there was a trend towards a more frequent use of TAF plus integrase inhibitors in period 2 (9.8% vs. 0%, *p* = 0.051). No statistical difference was detected in cure rates, treatment duration, lost to follow‐up and treatment failures. (Table 1)

**Conclusions**

From 2016 to 2017, HIV/HCV co‐infected patients have been more often treated with Ledipasvir/Sofosbuvir and underwent more elastography for pre‐treatment evaluation. Advanced liver disease was found less frequently in this period; which suggests that DAAs have been started earlier in the course of HCV infection in recent years. Despite these changes, there has been no difference in cure rates in our practice over the last 4 years.

**Table:** Abstract P014–Table 1. Clinical and treatment characteristics of HIV/HCV co‐infected patients and comparison by treatment period (period 1 and 2)

Variable	2014‐2017 (n=78)	Period 1: 2014‐2015 (n=37)	Period 2: 2016‐2017 (n=41)	*p*	OR (95% CI)
Age	55.64 ± 7.88	56.59 ± 6.46	54.78 ± 8.97	0.31	‐
Sex (male)	53 (67.9%)	25 (67.6%)	28 (68.3%)	0.95	0.97 (0.37 to 2.51)
Race					
a) Black	45 (57.7%)	19 (51.4%)	26 (63.4%)	0.28	0.61 (0.25 to 1.51)
b) White	13 (16.7%)	9 (24.3%)	4 (9.8%)	0.09	2.97 (0.83 to 10.65)
c) Hispanic	20 (25.6%)	9 (24.3%)	11(26.8%)	0.8	0.88 (0.32 to 2.43)
CD4 count	637.68 ± 334.35	672.08 ± 309.82	606.63 ± 355.97	0.39	‐
Received ART	75 (96.2%)	35 (94.6%)	40 (97.6%)	0.5	0.44 (0.04 to 5.03)
ART regimen					
a) TDF/FTC + NNRTI	15 (19.2%)	8 (21.6%)	7 (17.1%)	0.61	1.34 (0.43‐4.14)
b) TDF/FTC + PI	21 (26.92%)	9 (24.3%)	12 (29.3%)	0.62	0.78 (0.28 to 2.13)
c) TDF/FTC + InSTI	15 (19.23%)	9 (24.3%)	6 (14.6%)	0.28	1.88 (0.60 to 5.90)
d) TAF + InSTI	4 (5.13%)	0 (0%)	4 (9.8%)	0.05	1.97 (1.58‐2.47)
e) ABC/3TC + InSTI	7 (8.97%)	1 (2.7%)	6 (14.6%)	0.07	0.16 (0.02 to 1.41)
f) ABC/3TC + PI	4 (5.13%)	3 (8.1%)	1 (2.4%)	0.26	3.53 (0.35 to 35.52)
Prior Tx with IFN	22 (28.21%)	12 (32.4%)	10 (24.4%)	0.43	1.49 (0.55‐4.01)
Liver biopsy	33 (42.31%)	18 (48.6%)	15 (36.6%)	0.28	1.64 (0.66 to 4.06)
Elastography	15 (19.23%)	1 (2.7%)	14 (34.1%)	<0.001	0.054 (0.01 to 0.43)
Liver US	68 (87.18%)	36 (97.3%)	32 (78.0%)	0.011	10.13 (1.22 to 84.37)
Genotype					
a) 1a	47 (61.0%)	24 (64.9%)	23 (56.1%)	0.43	1.45 (0.58 to 3.61)
b) 1b	25 (32.5%)	10 (27.0%)	15 (36.6%)	0.37	0.64 (0.25 to 1.68)
HCV10log	6.18 ± 0.76	6.11 ± 0.71	6.25 ± 0.80	0.42	‐
Creatinine	1.05 ± 0.38	1.02 ± 0.34	1.08 ± 0.41	0.51	‐
Advanced liver disease (F3, F4)	28 (35.89%)	18 (48.6%)	10 (24.4%)	0.026	2.94 (1.12 to 7.68)
Cirrhosis	12 (15.38%)	7 (18.9%)	5 (12.2%)	0.41	1.68 (0.48 to 5.84)
HCV treatment					
a) Ledipasvir/Sofosbuvir	56 (71.79%)	21 (56.8%)	35 (85.4%)	0.005	0.23 (0.08 to 0.67)
b) Simeprevir/Sofosbuvir	12 (15.38%)	12 (32.4%)	0 (0%)	<0.001	2.64 (1.94 to 3.60)
c) PROD/RBV	2 (2.6%)	2 (5.4%)	0 (0%)	0.13	2.17 (1.702 to 2.77)
d) Elbasvir/Grazoprevir	2 (2.6%)	0 (0%)	2 (4.9%)	0.17	1.95 (1.57 to 2.43)
Treatment duration (12 weeks)	71 (91.03%)	33/36 (91.7%)	38/38 (100%)	0.07	0.47 (0.36 to 0.60)
Undetectable HCV at 4 weeks	57 (73.07%)	31/35 (88.6%)	26/32 (81.3%)	0.4	1.79 (0.46 to 7.03)
SVR12 (Intention to treat)	64 (82.05%)	31 (83.8%)	33 (80.5%)	0.71	1.25 (0.39 to 4.02)
SVR12 (treated analysis)	64/68 (94.12%)	31/34 (83.8%)	33/34 (97.1%)	0.3	0.31 (0.03 to 3.17)
Completed HCV Treatment	73 (93.59%)	35 (94.6%)	38 (92.7%)	0.73	1.38 (0.22 to 8.76)
Lost to follow‐up	4 (5.13%)	1 (2.7%)	3 (7.3%)	0.36	0.35 (0.04 to 3.54)
Failed HCV treatment	3 (3.85%)	3 (8.1%)	0 (0%)	0.06	2.21 (1.72 to 2.83)
No labs to assess SVR12	6 (7.69%)	2 (5.4%)	4 (9.8%)	0.47	0.53 (0.09 to 3.07)
Side Effects					
a) Headache	3 (3.84%)	2 (5.4%)	1 (2.4%)	0.5	2.29 (0.20 to 26.30)
b) Gastrointestinal	8 (10.26%)	7 (18.9%)	1 (2.4%)	0.017	9.33 (1.09 to 79.97)
c) Fatigue	10 (12.82%)	6 (16.2%)	4 (9.8%)	0.39	1.79 (0.46 to 6.92)

**References:**

1. Gonzales Zamora JA. Successful treatment of HIV‐/HCV‐coinfected patients in the era of direct acting antivirals: Is advanced liver disease still a limiting factor? Infect Dis (Lond). 2017 Nov; 12:1‐4.

2. Dae Won Jun, Won Young Tak, Si Hyun Bae, Youn Jae Lee. Recent trends in the treatment of chronic hepatitis C. Korean J Hepatol. 2012 Mar; 18(1): 22–28.

3. Yao X, Sangaralingham LR, Ross JS, Shah ND, Talwalkar JA. Adoption of new agents and changes in treatment patterns for hepatitis C: 2010‐2014. Am J Manag Care. 2016 Jun; 22(6):e224‐32.

## P015

### HBV monoinfection and HIV/HBV co‐infection in Brazil: which at are the differences?

**F Alvarenga Pinto, F Alves de Souza, M Santos, S Monzani Vivaldini, M Jorge de Queiroz, E Almeida and G Mendes Pereira**

Department of Surveillance, Prevention and Control, Ministry of Health of Brazil, Brasília, Brazil

**Background**

Hepatitis B virus (HBV) infection is one of the main causes of liver‐related mortality, resulting in almost 780,000 deaths annually worldwide. In 2015, there were 260 billion people identified with chronic HBV infection. The co‐infection with HIV in HBV chronic infected patients increases up to 5‐6 times the risk of progression to decompensated cirrhosis and liver cancer. We aim to describe the clinical and epidemiological characteristics of HBV‐monoinfected patients and HIV/HBV‐co‐infected patients in Brazil to identify their differences.

**Materials and methods**

Cross‐sectional study of HBV cases registered in the Notifiable Diseases Information System (SINAN) from 2007 to 2016 in Brazil.

**Results**

There were 8104 HIV/HBV‐co‐infected and 146,791 HBV‐monoinfected cases registered. Among HIV/HBV‐co‐infected cases; 81.6% were male; 54.2% white; 64.7% 30 to 39 years old; 31.4% 9 to 12 years’ education; 87.4% chronic infection and asymptomatic clinical form; 76.0% sexual transmission; and 15.8% transmission related to drug use. Among HBV‐monoinfected cases, 52.5% were male; 46.9% were young adults (including 20 to 39 years old); 55.8% sexual transmission; and only 3.6% related to drug use. The other characteristics were similar to HIV/HBV‐co‐infection: 53.8% white; 33.1% 9 to 12 years’ education; 84.6% chronic infection and asymptomatic clinical form.

**Conclusions**

The results show that HIV/HBV‐co‐infection is more prevalent among men, related to sexual transmission and use of drugs. The use of drugs is a continuous public health problem, and it is estimated that 12 million people use injecting drugs and 14% of them are living with HIV. In Brazil, harm reduction policies (implemented in the 90's) were relevant to efficient control of HIV, but now face new challenges such as crack use – which enlarges the risk of new infection and results in disease complications due to immune alterations. Although HBV vaccine is universal, Brazil still needs to implement prevention strategies targeting specifically the population observed in this study in order to offer the better care, reduce the risk of transmission and improve the access to diagnosis and treatment in the country.

## P016

### Monitoring hepatitis B and C infections among HIV patients: a proposed model for a cascade of care in viral hepatitis in the Dominican Republic

**L Tapia^1^, P Peña^1^, Y Matos^2^, K Valerio^3^, M Muñoz^4^, J Ledesma^5^, M Jimenez^5^ and R Paulino‐Ramirez^1^**

^1^Instituto de Medicina Tropical & Salud Global, Universidad Iberoamericana, Santo Domingo, Dominican Republic. ^2^Monitoring & Evaluation, Centro de Orientacion e Investigación Integral, Santo Domingo, Dominican Republic. ^3^Clinical Unit, Centro de Orientación e Investigación Integral, Santo Domingo, Dominican Republic. ^4^Research Department, Centro de Orientacion e Investigacion Integral, Santo Domingo, Dominican Republic. ^5^Dirección General de Control de ITS/SIDA, Ministry of Health, Santo Domingo, Dominican Republic

**Background**

Although the hepatitis B vaccine began widely used in high‐income countries in the 1980s, almost 20 years later it was still rarely used in low‐ and middle‐income countries. The main reasons behind these gaps included limited money for immunization, a lack of the infrastructure needed to carry out effective immunization programs, and a lack of political interest in immunization [1]. It is estimated a HBV prevalence of 2 to 4% in the Caribbean, and for HCV it is around 1.2% in the Latin‐American region [2]. In the DR prevalence of both HIV and/or HBV/HCV are higher among GMT persons [3].

**Materials and methods**

Patients enrolled in care in a community‐based clinic in Santo Domingo were evaluated for clinical indicators of HBV/HCV/Syphilis and HIV infections. A proposed model of cascade of care was proposed with the indicators found (Figures 1, 2)

**Results**

A total of 2435 cases of HIV (+) patients were evaluated. Serological tests demonstrating HBsAg detection was 91% (n = 2238), and co‐infection HIV/HBV was 63% (n = 29), and Tenofovir‐based ARV regimens among HBsAg (+) was 49% (n = 14) The mean age in the HIV/HBV/HCV cohort was 44 years old, 71.8% were male, and of those 12% was MSM. HCV/HIV co‐infection was 41%(n = 19), and the frequency of HIV/HBV/HCV was only 4.3%. In all cases there were no HBV or HCV viral loads available, and none of them with treatment for HCV infection. Among those with HBV infection and HIV 48% was on ARV Tenofovir‐based treatment.

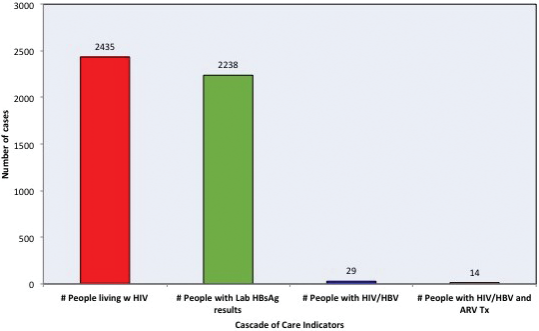

**Abstract P016–Figure 1. Cascade of Care for HIV/Hepatitis B Infection**

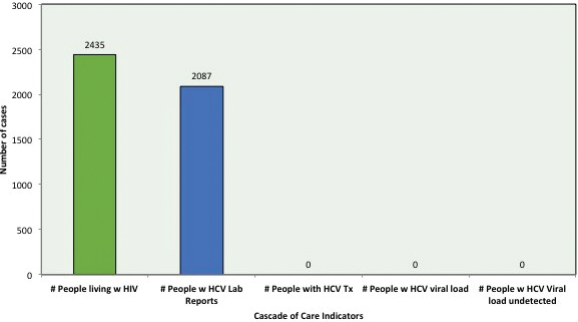

**Abstract P016–Figure 2. Proposed Cascade of Care for HIV/Hepatitis B Infection.**

**Conclusions**

This study reveals that it is needed more investment on early detection and intensive screening for both HBV and HCV in the context of non‐HIV positive patients. The proposed cascade of care to monitor interventions requires a scale‐up in case reporting and early treatment in each scenario.

**References**

1. Miller W, Buckingham L, Sánchez‐Domínguez M et al. Systematic review of HIV prevalence studies among key populations in Latin America and the Caribbean. Salud Publica Mex2013;55(Suppl 1):S65–78.

2. Zoni AC, Gonzalez MA, Sjogren HW. Syphilis in the most at‐risk populations in Latin America and the Caribbean: a systematic review. Int J Infect Dis2013;17:e84–92

3. Mendez‐Sanchez N, Gutierrez‐Grobe Y, Kobashi‐Margain RA.Epidemiology of HCV infection in Latin America. Ann Hepatol2010;9(Suppl):27–9.

## P017

### Epidemiological characteristics of HIV‐positive/hepatitis C virus (HCV) co‐infection in Hispanic women

**N Centeno‐Alvarado^1^, V Wojna^2^, E Ramos‐Perez^1^, J Barreto^1^ and R Rodriguez‐Benitez^2^**

^1^University of Puerto Rico, San Juan, Puerto Rico. ^2^NeuroAIDS Program ‐ Medical Science Campus, University of Puerto Rico, San Juan, Puerto Rico

**Background**

In the era of combined antiretroviral treatment (cART), survival rate has increased [1,2]. One quarter of the population with HIV+ in the United States are also co‐infected with HCV [3]. When comparing HIV+/HCV co‐infected women with HCV‐uninfected women, studies have shown that co‐infected women are more susceptible to health complications [4,5,6]. In addition, studies have shown a significant difference in mortality in women with HIV+/HCV versus HIV+ alone [7].

**Materials and methods**

A secondary data analysis was performed using information collected from the Puerto Rican HIV+ women cohort from the University of Puerto Rico Medical Science Campus. SPSS© version 24 and Stata© software's were used. The group of seropositive (HIV+) women (n = 43) was stratified in HIV+/HCV co‐infected women (n = 15) and HIV+ mono‐infected women (n = 28). Evaluation methods included medical history, neurological exam, viral‐immune profiles, neuropsychological tests and others. Descriptive statistics were performed.

**Results**

A proportion of 35% (95% CI, 0.21 to 0.51) of our sample of HIV+ women had HIV+/HCV co‐infection, whereas 65% of women had HIV+ mono‐infection (95% CI, 0.49, 0.78). Patients with HIV+/HCV co‐infection characteristics were: age (Mean 46.93, SD = 7.91 years), Depression Scale [BDI‐II] scores (Mean 12.47, SD = 9.25), CSF Viral (Mean 1.51, SD = 1.45), CD4 Nadir (Mean 363.36, SD = 252.02), current CD4 (Mean 679.83, SD = 435.79), and CNS Penetration‐Effectiveness (CPE) of Antiretroviral Therapy (ART) (Mean 3.5, SD = 2.76). In addition, 66% (n = 10) of our sample with HIV+/HCV co‐infection use ARTs, 80% (n = 12) have impaired cognition, and 73% (n = 11) do not use drugs. HIV+ mono‐infected women characteristics were: age (Mean 41.29, SD = 9.03 years), Depression Scale [BDI‐II] scores (Mean 12.86, SD = 11.68), CSF Viral (Mean 0.94, SD = 0.92), CD4 Nadir (Mean 389.83, SD = 240.35), current CD4 (Mean 651.48, SD = 299.15), and CPE of ART (Mean 3.40, SD = 2.75). In addition, 54% (n = 15) of our sample of women with HIV+ mono‐infection use ARTs, 64% (n=18) have cognitive impairment, and 64% (n = 18) do not use drugs.

**Conclusions**

Our findings suggest that larger studies are required to ensure a better assessment of the HIV+/HCV co‐infected population characteristics to ensure prevention measures. Especially, in Hispanic women due to the prevalence of HIV+/HCV co‐infection. 

**References**

1. Underwood J, Robertson K, Winston A. Could antiretroviral neurotoxicity play a role in the pathogenesis of cognitive impairment in treated HIV disease? AIDS [Internet]. 2015 [cited 3 January 2018];29(3):253‐61. Available from: https://www.ncbi.nlm.nih.gov/pubmed/25426811

2. Woods S, Moore D, Weber E, Grant I. Cognitive Neuropsychology of HIV‐Associated Neurocognitive Disorders. Neuropsychol Rev [Internet]. 2009 [cited 3 January 2018];19:152‐168. Available from: https://www.ncbi.nlm.nih.gov/pubmed/19462243

3. HIV and Viral Hepatitis [Internet]. cdc.gov. 2016 [cited 2 January 2018]. Available from: https://www.cdc.gov/hiv/pdf/library/factsheets/hiv-viral-hepatitis.pdf

4. Graham CS, Baden LR, Yu E, et al. Influence of human immunodeficiency virus infection on the course of hepatitis C virus infection: a meta‐analysis. Clin Infect Dis. 2001;33:562–569.

5. Deng LP, Gui XE, Zhang YX, Gao SC, Yang RR. Impact of human immunodeficiency virus infection on the course of hepatitis virus infection: a meta‐analysis. World J Gastroenterol. 2009;15:996‐1003.

6. Sulkowski, MS, DL Thomas, RE Chaisson, and RD Moore. Hepatotoxicity associated with antiretroviral therapy in adults infected with human immunodeficiency virus and the role of hepatitis C or B virus infection. JAMA 283, no. 1 (January 5, 2000): 74‐80.

7. Taylor, Lynn E., Tracy Swan, and Kenneth H. Mayer. HIV Coinfection With Hepatitis C Virus: Evolving Epidemiology and Treatment Paradigms. Clinical Infectious Diseases 55 (July 2012): S33‐42.

## HIV and Tuberculosis

## P019

### Severe HIV‐associated pulmonary tuberculosis, 2006–2016

**J Ricart^1^, M Sanchez Cunto^1^, R Gregori Sabelli^2^, C Dominguez^2^, P Saul^2^, J Chomyn^2^, M Nano^2^, N Chacon^2^, V Chediack^2^ and E Cunto^2^**

^1^Infectious Diseases, Infectious Hospital FJ Muñiz, Buenos Aires, Argentina. ^2^Intensive Care Unit, Infectious Hospital FJ Muñiz, Buenos Aires, Argentina

**Background**

Globally people living with HIV (PLHIV) are 19 times more likely to fall ill with Tuberculosis (TB) than those without HIV. In 2015 0.4 million PLHIV estimated to have died from TB [1]. The aim is to evaluate the clinical features of severe HIV‐associated pulmonary TB and determine factors related with mortality.

**Materials and methods**

Among 1122 PLHIV admitted to the intensive care unit (ICU) of an Infectious Diseases Hospital between 2006 and 2016, 135 had pulmonary TB (PTB). Comorbid diagnoses, clinical features, radiological and laboratory investigations, and outcomes were reviewed. Univariate analysis was performed to identify factors related to death. We performed descriptive statistics on percentage (%), median (Me), mean (M) and range. A *p* value of <0.05 was considered significant.

**Results**

Incidence of 0,1203, increasing 85% from 2006 to 2016. 62% were male. Me/M age were 40/38 years (14 to 74). The median duration of ICU care: 7 (1 to 35) days. 76% had evolved to AIDS for more than a year. 95% presented hypoalbuminemia (< a 3.5 g/l), weight loss >10% in last 6 months and/or Karnofsky score ≤50. Only 10% were receiving highly active antiretroviral therapy (HAART). 7% had CD4 ≥200 cells/mm^3^, none of them died, 93% had CD4 <200 cells and 50% died. 8% were co‐infected with hepatitis C, 19% had liver failure (LF) and 88% of them died. Respiratory insufficiency (RI) was observed in 59% and 66% died. 42% required mechanical ventilation (MV). 84% had at admission an APACHE II score (Acute Physiology and Chronic Health Evaluation II) ≥13 points. Overall mortality was 51%. Evolution to AIDS greater than a year, not receiving HAART, weight loss, hypoalbuminemia, Karnofsky score, CD4 count, MV, RI, LF and APACHE II score ≥13 were significantly associated with mortality.

**Conclusions**

Higher incidence of PTB in PLHIV/AIDS in 2015 to 2016 was observed. Poor adherence to HAART, deficient immunological and nutritional status and severe PTB were associated with mortality. We encouraged to achieve a proper and early diagnosis and treatment of both pathologies, to improve prognosis of these patients.

**Reference**

1. Bhurayanontachai and Maneenil. Factors influencing mortality of active pulmonary tuberculosis with acute respiratory failure. J Thorac Dis 2016;8(7):1721‐1730

## P020

Abstract Withdrawn

## P021

### HIV/TB co‐infection

**A Ciappina, A Bobatto, F Bechini, B Boggia, M Echaide, J Iriart, A Lux, L Urbina, G Corral, M Hualde, C Miglioranza and S Aquilia**

HIGA Dr Alende, ID, Mar del Plata, Argentina

**Background**

One third of the world population is infected with *Mycobacterium tuberculosis,* 95% in developing countries. Several factors worsen the scenario: the HIV pandemic, the poverty and poor access to the health system. In 2016, 6.3 million new cases of tuberculosis were reported worldwide, 10% of them HIV co‐infected; 1.3 million deaths occurred and 374,000 happened in this group. In addition, the incidence of TBMR cases is increasing steadily. Annually 11,000 new TB cases are reported in Argentina; 30% of the newly diagnosed cases at our institution occur in HIV+ patients.

**Materials and methods**

The objective was to describe the social‐demographic variables, clinical presentation and outcomes of HIV/TB co‐infected patients cared for at a General Hospital. Observational, descriptive, cross sectional study of HIV/TB co‐infected patients in the January 2009 ‐ January 2018 period. World Health Organization (WHO) 2017 criteria were used to define the variables.

The continuous and categorical variables were analysed using EPI‐info.

**Results**

A total of 163 patients were included, inpatients:145 (89%). Male: 107, female: 45, Transgender: 19. Mean age: 38.3 years (SD:10.3). Peruvian nationality: 15 (all of them transgender‐sexual workers). HIV diagnosis prior to TB:111, C3 category: 83%, 74% had CD4<100 cel/mm^3^. The 81% of patients had HAART prescription >6 months. VL <50:10%. PI‐based regimen: 40%. Confirmed TB diagnosis: 137; positive smear: 66%; Negative smear/positive culture: 20%; biopsy: 14%. Clinical presentation: respiratory symptoms: 48%, fever: 37%, wasting syndrome: 31%. Localization: pulmonary: 41%; extra‐pulmonary: 26%. Pulmonary and extra‐pulmonary concomitant: 28%. Positive culture and sensitivity: 46 (28%); overall resistant 30%: Mono‐resistant 4, poly‐resistant 2, MDRTB 5, XDRTB 3. Primary resistance: 22%. SAEs occurred in 21 patients and they had to discontinue their treatment. Outcomes: Favourable: 87 (53%); unfavourable: 76 (47%): lost in follow‐up 29, deaths 47.

**Conclusions**

Patients were hospitalized at advanced HIV and TB stages, including cases of MDRTB and XDRTB. We describe a high number of resistant TB cases in this serie, this could be explained due to the increasing migratory flow from the region with high incidence of MDRTB. We must encourage the active search of cases for early diagnosis and compliance with DOT to guarantee the continuity of treatment, improve the cure rate and prevent transmission, by articulating all levels of care. The increase in resistance, drug interactions and SAEs are a challenge for the treatment of these two associated pathologies.

## HIV and Women, Including MTCT

## P022

### Prevalence of comorbid conditions in a cohort of women living with HIV in Argentina

**R Mauas, M Laurido, L Cabral, V Cacciari and I Cassetti**

Helios Salud, Infectious Diseases, Buenos Aires, Argentina

**Background**

A rising number of women living with HIV (WLHIV) are now reaching menopausal age. Comorbid conditions are of increasing importance in clinical HIV practice. However, data regarding their impact in this population are limited. The aim of this study was to describe the epidemiological and clinical characteristics of a cohort of adult WLHIV receiving care at our institution.

**Materials and methods**

Retrospective, observational study including WLHIV over 50 years old assisted at an HIV reference centre in Buenos Aires, Argentina from 1997 to 2017. Clinical, laboratory and demographic data from electronic medical charts were reviewed. We evaluate the prevalence of common comorbidities. Cardiovascular disease and fracture risk‐assessment were performed for each subject using the ACC/AHA ASCVD Plus Risk Estimator and FRAX® respectively. The information was collected in an Ad Hoc database and T‐test, ANOVA, Chi^2^ , Fisher exact or Mid P tests and maximum likelihood odds ratio were used as appropriate.

**Results**

Two hundred and fifty patients were included: mean age 58.1 years; AIDS at diagnosis, 116 (46.4%); on cART, 246 (98.4%); HIV‐1 RNA <20 c/ml, 217 (88.2%); mean current CD4 count, 673 cells/μl; mean age at menopause, 49 years. Furthermore demographic and clinical characteristics are shown (Table 1). We observed a mean of 2,7 comorbidities per subject. Their prevalence are summarized (Figure 1). Patients with dyslipidaemia were more likely to have been exposed to thymidine analogs/1st. generation PI (OR 2.34; 95% CI: 1.15 to 4.82; *p *= 0.019) or abacavir (OR 2.29; 95% CI:1.28 to 4.12; *p*=0.005), but less likely to have been exposed to tenofovir (OR 0.44; 95% CI: 0.26 to 0.75; *p *= 0.002). Patients that experienced psychiatric disorders were more likely to be on PI‐based regimens (OR 2.35; 95% CI: 1.35 to 4.15; *p *= 0.002) rather than on efavirenz‐based regimen (OR 0.52; 95% CI: 0.30 to 0.89; *p *= 0.017), as prescription habits. Mean risk scores values: ASCVD plus, 4.5%; FRAX (Hip), 1.1%; FRAX (Major osteoporotic), 4.1%. As expected, osteoporosis was associated with age and vitamin D insufficiency (*p *< 0.05).

**Conclusions**

Although half of the adult WLHIV assisted at our institution had advanced disease at presentation, most of them achieved viral suppression and immune recovery. Multi‐morbidity was observed with dyslipidaemia, hypertension, bone and psyquiatric disorders as the most prevalent chronic conditions. Healthcare providers should be aware of a comprehensive approach need for adult HIV‐infected women.

**Table:** Abstract P022–Table 1. Demographic and clinical characteristics

Variable	Result
Mean age, y (SD)	58.1 (6.1)
Mean time since HIV diagnosis, y (SD)	13.8 (6.9)
Mode of HIV transmission, n (%)	
Heterosexual	238 (95.2)
IDUs	4 (1.6)
Transfusional	3 (1.2)
Unknown	5 (2)
Mean BMI, kg/m^2^ (SD)	26.9 (5.1)
Habits, n (%)	
Current or former smoker	116 (46.4)
History of alcohol/substance abuse	22 (8.8)
AIDS at diagnosis, n (%)	116 (46.4)
On cART, n (%)	246 (98.4)
Mean total cART duration, y (SD)	11.7 (5.9)
Mean current cART duration, y (SD)	4.8 (3.7)
Current ART regimens, n (%)	
2 or 3 NRTIs + 1 NNRTI	134 (54.5)
2 or 3 NRTIs + 1 PI	73 (29.7)
2 NRTIs + 1 INI	19 (7.7)
2 NRTIs + MVC	5 (2)
1 PI + 1 INI	6 (2.4)
1 PI + MVC	1 (0.4)
1 PI + 3TC	2 (0.8)
Others	6 (2.4)
First line cART, n (%)	46 (18.7)
Thymidine analogs or 1st. generation PI past exposure, n (%)	213 (86.6)
Plasma HIV‐1 RNA < 20 copies/ml, n (%)	217 (88.2)
Mean current CD4 T‐cell count, cells/μl (SD)	673 (312)
Women who experienced an ARV related adverse event, n (%)	179 (72.8)

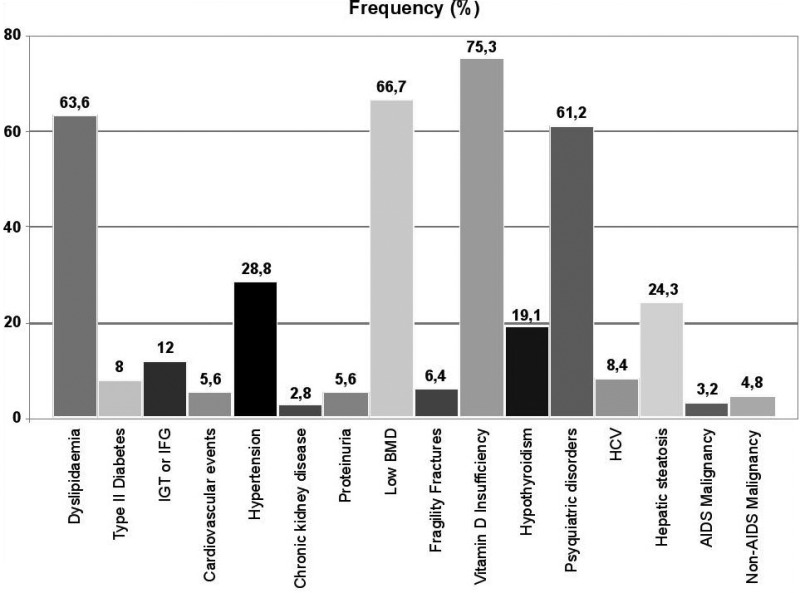

**Abstract P022–Figure 1. Prevalence of comorbid conditions.**

## P023

### Factors associated with virological failure in women with HIV

**T Cabrera^1^, U Ramos^2^, C Nava^3^, A Almaraz^1^, F Badial‐Hernández^5^, E Langarica^6^ and A Gonzalez^7^**

^1^Obstetrics and Gynaecology, Clínica Especializada Condesa, Mexico City, Mexico. ^2^Directorate, Clínica Especializada Condesa, Mexico City, Mexico. ^3^ Obstetrics and Gynaecology, Clínica Especializada Condesa Iztapalapa, Mexico City, Mexico. ^5^Medical Directorate, Clínica Especializada Condesa Iztapalapa, Mexico City, Mexico. ^6^Trabajo Social, Clínica Especializada Condesa, Mexico City, Mexico. ^7^Executive Directorate, Center for the Prevention and Integral Attention of HIV/AIDS of the Federal District, Mexico City, Mexico

**Background**

UNAIDS indicated that in 2016 there were 36.7 million people living with a diagnosis of HIV. Women represent 48.5% of this group. In our country, 19.6% are women. It has been shown that women present a greater risk of abandoning antiretroviral treatment [1].

**Materials and methods**

An observational cross‐sectional study, carried out among women patients at the Condesa Clinics between March 2014 and November 2017. All patients had previously been pregnant and had started HAART (high active antiretroviral treatment) at least six months before and possessed complete medical records. Convenience sampling; prevalence, odds ratio, chi^2^ and attributable risk were analysed using SPSS21.

**Results**

One hundred and three women HIV patients from the Condesa Specialized Clinic (CSC) and CSC–Iztapalapa. Median age, 24.5 years (SD 5.8). Education: 40% completed middle school. Unpaid employment: 61.2%. 73% have a stable partner. 69% with virologic suppression and 31% with virologic failure. At the moment of failure, the average viral load was 5,169c/ml (range: 316 to 188,910); average CD4 was 381c/µl (range: 70 to 591). On average, 11 months (range: 8 to 60) passed between the start of antiretroviral treatment and failure (Table 1).

**Table:** Abstract P023–Table 1

Factors Associated with Virologic Failure	Prevalence Among Patients Exposed to Factor	Prevalence Among Non‐Exposed	Odds Ratio (OR)	X2	*p*	Attributable risk among exposed
Domestic violence	48%	25%	1.9 CI 95% (1.1 to 3.3)	2.2	0.02	47%
Illegal drug use	60%	27%	2.2 CI 95% (1.1 to 3.9)	6.7	0.04	54%
Legal drug use	61%	27%	2.2 CI 95% (1.2 to 3.8)	2.4	0.01	55%
HAART with protease inhibitor	40%	18%	2.2 CI 95% (1.1 to 4.4)	2.4	0.01	54%

The probability of virologic failure in women who experienced domestic violence was 1.9 OR, while among illegal and legal drug users, as well as those receiving antiretroviral treatment with protease inhibitor (IP), the probability of virologic failure was 2.2 OR. 31% of patients participated in a women's support group, and 22% of those who attended had virologic failure compared with 35% among the women who did not participate in the group. 

**Conclusions**

In this study, we observed a high prevalence of virologic failure, while other studies have shown greater vulnerability among women with HIV with less education and higher rates of domestic violence [2]. One study reports a 29% virologic failure rate in women who continue treatment for 12 months postpartum. Identifying the associated factors is useful in developing intervention strategies related to the factors identified in reducing virologic failure.

**References**

1. Valenzuela‐Lara, M. Determinación de factores asociados al abandono del tratamiento antirretroviral en pacientes con VIH/sida registrados en la base de datos salvar. Tesis de maestría. México: Instituto Nacional de Salud Pública (INSP); 2014.

2. Bautista‐Arredondo S, Servan Mori E, Beynon F, González Rodríguez A, Volkow Fernández P. A tale of two epidemics: gender differences in socio‐demographic characteristics and sexual behaviors among HIV positive individuals in Mexico City. Int J Equity Health. 2015; 14:147.

## HIV and Vulnerable Populations

## P024

### Overview of healthcare linkage, retention and adherence in young people living with HIV in Brazil

**A Kolling, M Camelo Madeira de Moura, J da Silva Netto, R Vianna Brizolara, A Pati Pascom, F de Barros Perini and A Schwartz Benzaken**

Department of STI, AIDS and Viral Hepatitis, Ministry of Health, Brasília, Brazil

**Background**

After recommending treatment for all PLHIV in 2013, Brazil has progressively expanded ART's coverage. HIV care cascade, tool for decision and health policy making, represents results of interventions and helps to identify crucial points to qualify healthcare for PLHIV. In 2016, 830,000 PLHIV was estimated in Brazil, of these 694,000 (84%) was diagnosed; 655,000 (79%) was linked to health services; and 563,000 (68%) was retained. Since then, there is a decrease of untreated PLHIV in all ages, however the proportion of PLHIV between 18 and 24 yo without ART is 2.5 times higher compared to PLHIV over 60 years old. One of the challenges of the Brazilian public system is to increase the number of young people with TARV adherence, linked and retained in health services.

**Materials and methods**

A cross‐sectional study was carried out to estimate retention and adherence in PLHIV older than 18 years. The results were obtained from data crossing of the Siscel (a national laboratory system that shows individual CD4 count and HIV viral load results) and the Siclom (a national antiretroviral delivery control system), between 2009 and 2017 (first semester).

**Results**

There is a progressive improvement in healthcare linking, retention and adherence of PLHIV aged 18 to 24 years between 2009 and 2017. Despite the poorest adherence proportions, young people demonstrated a considerable improvement in sufficient adherence, rising from 47% in 2009 to 64% in 2016 and lost to follow up from 26% to 15% (Figure 1.)

In 2016, approximately 43,000 young people were linked, 78% were retained in health service, 66% were in ART and 57% were suppressed. The proportion of PLHIV (18 to 24 yo) in ART with HIV viral load < 200 copies/ml is lower when compared to the other ages (Figure 2). PLHIV aged 18 and 24 who started ART in 2009, 52% were retained after 12 months, rising to 81% in ART started in 2015. Reduction in retention was observed after 24 months and 5 years after the initiation of ART. After two years, 74% of PLHIV aged 18 to 24 who started ART in 2014 remained retained, and 61% who started ART in 2011 remained retained after five years.

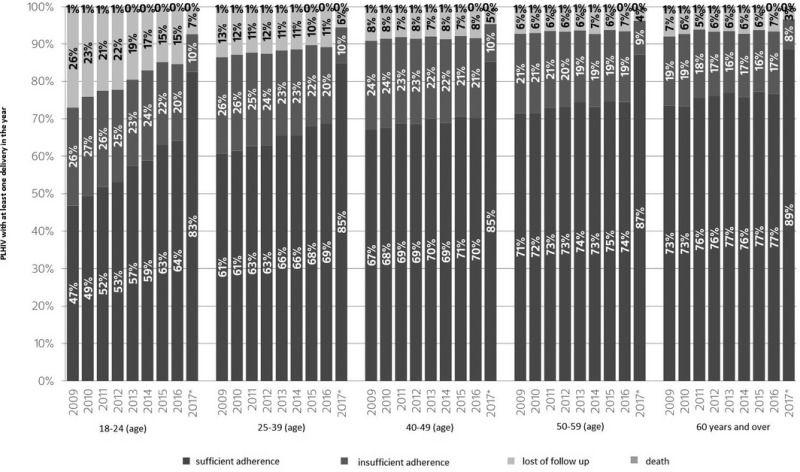

**Abstract P024–Figure 1. Status of PLHIV aged 18 years and over with at least one delivery in the year, at the end of each year, related to ART and death, by age group. Brazil, 2009 to 2017.**

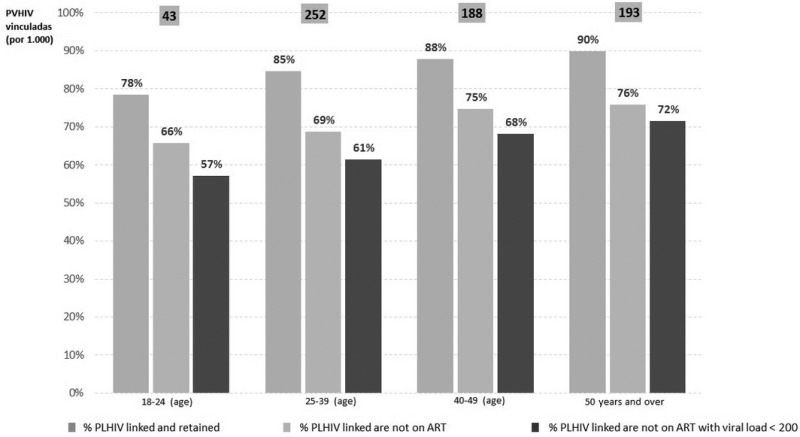

**Abstract P024–Figure 2. HIV care cascade, calculated in relation to PLHIV linked to health services, by age group. Brazil, 2016.**

**Conclusions**

Despite the gradual improvement of the indicators analysed in PLHIV aged 18 to 24 years, it is essential to strengthen actions directed to young people, increasing access to health services, to ensure linkage and retention of these young people, directly impacting on quality of life.

## P025

### Retention in care trends and viral suppression in newly admitted HIV patients in a tertiary centre in Mexico City

**E Barlandas^1^, Y Caro‐Vega^1^, P Belaunzarán‐Zamudio^1^, L Guerrero‐Torres^2^, J Sierra‐Madero^1^ and B Crabtree‐Ramírez^1^**

^1^Infectious Diseases, Instituto Nacional de Ciencias Médicas y Nutrición “Salvador Zubirán”, Mexico City, Mexico. ^2^Internal Medicine, Instituto Nacional de Ciencias Médicas y Nutrición “Salvador Zubirán”, Mexico City, Mexico

**Background**

The continuum of care has become an important tool for evaluating HIV care locally and regionally. We aimed to evaluate retention and viral suppression one year after enrolment in a tertiary centre in Mexico City, and assess factors associated with increased retention.

**Materials and methods**

This was a retrospective, cross‐sectional analysis of a cohort of adults receiving care for HIV, enrolled in a Mexican HIV Clinic between 2002 and 2015. Main outcomes were retention in care (RiC) and Viral suppression (VS) during the first year of care. RiC was defined as at least two HIV care visits annually, at least 90 days apart. VS was defined as HIV‐1RNA <50 copies/mL, measured between 6 and 15 months after enrolment; if more than 1 determination per year, the last HIV‐1RNA was considered. We used multivariable logistic regression models, stratified by gender, to study associations of outcomes and age, previous ART, route of HIV transmission, AIDS at enrolment, year of enrolment, socio‐economic status and education.

**Results**

A total of 2,095 patients were enrolled between Jan. 2002 and Dec. 2015. Most were male (88%) and were infected through sex with other men (71%); 69% were ART naïve. We analysed 1913 patients with complete data. No previous ART exposure was independently associated with RiC (*OR:* 2.58*, p = *0.02 among women and 1.99*, p < *0.001 among men) and VS (*OR:* 1.99*, p = *0.44 for women and 1.57*, p < *0.001 for men). In men, but not in women, RiC improved significantly along time [*OR:*1.62*, p = *0.011 for 2005 to 2008 period, *OR:*1.83*, p = *0.006 for 2009 to 2011 and *OR:*2.1*, p < *0.001 for 2012 to 2015 (Figure1)]. Among women, higher socio‐economic status (middle vs. low, *OR* 3.7*, p = 0.04*) and higher education (>12 years, *OR* 3.7*, p = *0.04) were associated with RiC. More recent enrolment in care was independently associated to VS among men (*OR:*1.5*, p = *0.008 for 2005 to 2008 period, *OR:*1.82*, p < *0.001 for 2009 to 2011, *OR:*2.96*, p < *0.001 for 2012 to 2015) but not for women, except for the last period (*OR* 3.08*, p = 0.01*). AIDS at enrolment was associated with VS for both genders *(p < *0.001).

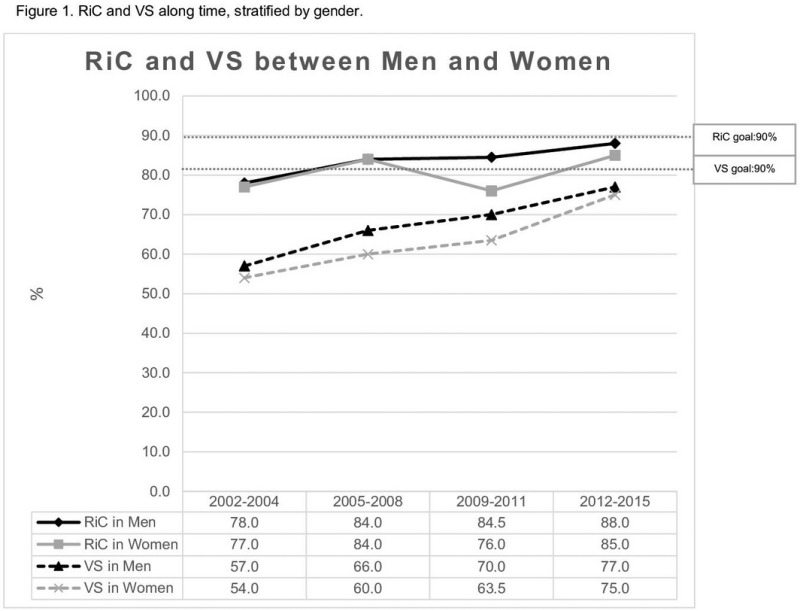

**Abstract P025–Figure 1. RiC and VS along time, stratified by gender.**

**Conclusions**

Retention in care and virological suppression has improved over time for men but not for women. Our study highlights that the previously documented vulnerability in women living with HIV in Mexico may have a negative impact in the continuum of care.

## P026

Abstract Withdrawn

## P027

### Advanced presentation among HIV/AIDS patients despite universal access to antiretroviral therapy in northern Mexico

**E Gonzalez‐Fernandez, I Medina‐Piñon and J Ramos‐Jimenez**

Infectious Diseases, Jose E Gonzalez University Hospital, Monterrey, Mexico

**Background**

Hospitalizations are an important indicator of healthcare quality and access for HIV/AIDS patients [1]. We sought to analyse epidemiological data and to determine the causes of hospitalization and advanced presentations of HIV/AIDS patients based on CD4+ T‐cell counts from 2013 to 2017.

**Materials and methods**

We conducted a retrospective study of HIV patients admitted in a third‐level University Hospital in north‐eastern Mexico. Hospitalization records were collected for all 385 inpatient admissions from 2013 to 2017 that were diagnosed with HIV upon admission, or prior to admission, by matching the hospitalization records using the CID ‐10 codes for HIV infection. Demographic and clinical characteristics were analysed for first‐time hospitalizations. Patients’ most recent CD4+ T‐cell count (within 6 months), if available, was documented. All hospitalizations were classified according to age group, gender, lengths of stay and primary diagnosis (AIDS‐defining illness and Non‐AIDS defining infection).

**Results**

Among 385 patients evaluated, 189 had CD4+ T‐cell lymphocyte counts available (49.1%), and the average CD4+ count was 198.8 cells/mm^3^. Patients with CD4+ counts below 200 cells/mm^3^ constituted 65.1% (n = 123). Those with CD4+ counts of 200 to 499 cells/mm^3^ represented 24.3% (n = 46), and only 10.6% (n = 20) had counts above 500 cells/mm^3^. AIDS‐defining illnesses were the most common cause of hospitalization (56.6%, n = 218), followed by Non‐AIDS defining infections (20.3%, n = 78). Of those patients, males represented 79.3% (n = 174) and 83.3% (n = 65) respectively. A total of 86 fatalities were reported during the period analysed, amounting to a mortality of 22.3%. The age group with the highest mortality rate was that of 41 to 50 year olds (30.2%, n = 26). Most hospitalizations had a length of stay of 11 days or more (46.0%, n = 177), and a mean length of stay of 13 days. A total of 197 (51.2%) patients admitted were unaware of their HIV diagnosis. The majority of patients were local state residents (95.6%, n = 368), and a small number of patients were from a different region (4.4%, n = 17).

**Conclusions**

Despite the availability of federally funded antiretroviral treatment for HIV in Mexico for all medically insured and non‐insured patients since the year 2000 [2], the incidence of hospitalization in advanced stages of disease and mortality for HIV/AIDS patients remains significantly high. This represents an urgent need for more efficient preventive and early detection strategies that may lead to reduced advanced presentations of the disease and improved outcomes.

**References**

1. Lazar R, Kersanske L, Xia Q, Daskalakis D, Braunstein S. Hospitalization Rates Among People With HIV/AIDS in New York City, 2013. *Clinical Infectious Diseases,* 65(3), 469‐476. Available from: https://doi.org/10.1093/cid/cix343

2. Rodriguez C ed., Hermelinda B ed. *VIH/SIDA y salud publica: Manual para personal de salud. *2nd ed. Mexico. Centro Nacional para la Prevencion y el Control del VIH/SIDA; 2009

## P028

Abstract Withdrawn

## P029

### Cardiovascular risk assessment in transgender women in a HIV/STI clinic in a public hospital in Argentina

**J Ballivian, J Toibaro, G Viloria, S Ivalo and M Losso**

HIV Unit, Hospital JM Ramos Mejia, Buenos Aires, Argentina

**Background**

Cardiovascular disease (CVD) is one of the main causes of morbi‐mortality in general population worldwide. As lifespan of people living with HIV have improved, prevalence and incidence of CVD is expected to increase in the future. Although there are several risk functions to assess long‐term CVD‐risk, to our knowledge, there is no such data about transgender women (TW) in our country. We aim to describe 30‐year CVD‐risk of TW at an HIV/STI clinic in Argentina.

**Materials and methods**

We performed a cross‐sectional retrospective study to evaluate CVD‐risk among TW at a HIV/STI clinic in Buenos Aires, Argentina, from 2014 to 2017. TW between 20 and 60 years at first visit to the clinic were included. We ascertained demographic characteristics, traditional CVD risk factors, HIV status and use of hormone therapy (HT). Categorical variables were described using absolute and relative frequencies and compared by χ2 test or Fisher's exact test according to expected values. Continuous variables were described using medians and interquartile ranges (IQR) and compared by t‐test or Mann‐Whitney test according to normality of variables. A two‐sided p‐value of < 0.05 was considered significant. Long‐term CVD risk was assessed with Framingham 30‐year risk score (30FMS) using body mass index (BMI). Both “hard” (coronary death, myocardial infarction, stroke) and “general” (“hard” + coronary insufficiency, angina, transient ischemic attack, peripheral artery disease and heart failure) CVD risks were analysed as continuous variables.

**Results**

One hundred and eleven TW were eligible for this analysis, 37 (33.3%) were HIV‐positive and 44 (39.6%) reported use of HT. Median age was 31 years (IQR 27 to 37). The median 30‐year risk of “hard” CVD in TW receiving HT was 0.1 (IQR 0.04 to 0.135) and 0.06 (IQR 0.03 to 0.16) among TW not receiving HT. We found no statistically significant differences in the median 30‐year risk of “hard” and “general” CVD in these groups (*p*‐value = 0.3356 and *p*‐value = 0.2981 respectively). The median 30‐year risk of “hard” CVD in HIV‐positive TW was 0.05 (IQR 0.03 to 0.08) and 0.1 (IQR 0.03 to 0.17) among negative TW. We found statistically significant differences among these groups (*p*‐value = 0.0311).

**Conclusions**

Our study found lower median 30‐year CVD risk among HIV‐positive TW. Traditional risk scores do not consider the increased CVD risk of HIV population, this result could be related to this limitation of the score. Regarding use of HT we did not detect differences. However, our study provides new insights about a key population and HIV. Furthermore studies are needed, specially to estimate the complex role of HT and HIV in TW CVD risk.

**References**

1. Tunstall‐Pedoe H. Preventing Chronic Diseases. A Vital Investment: WHO Global Report. Geneva: World Health Organization, 2005. 

2. Eyawo O, Franco‐Villalobos C, Hull MW, Nohpal A, Samji H, Sereda P, Lima VD, Shoveller J, Moore D, Montaner JS, Hogg RS. Changes in mortality rates and causes of death in a population‐based cohort of persons living with and without HIV from 1996 to 2012. BMC infectious diseases. 2017 Dec;17(1):174.

3. Pencina MJ, D'agostino RB, Larson MG, Massaro JM, Vasan RS. Predicting the 30‐year risk of cardiovascular disease: the Framingham Heart Study. Circulation. 2009 Jun 23;119(24):3078‐84.

## P030

### First intervention in a gay sauna with rapid tests for HIV infection in the city of Córdoba, Argentina

**M Pedrola^1^, N Haag^1^, J Garcia^2^, E Munoz^3^, C Bonilla^4^, G Alaniz^5^ and M Laurido^4^**

^1^AIDS Healthcare Foundation, Venado Tuerto, Argentina. ^2^AIDS Healthcare Foundation, Córdoba, Argentina. ^3^Voluntarixs Córdoba, Head Office, Córdoba, Argentina. ^4^AIDS Healthcare Foundation, Buenos Aires, Argentina. ^5^AIDS Healthcare Foundation, Bariloche, Argentina

**Background**

It is well known that in Argentina men who go to gay saunas engage in unsafe sex and tend to have multiple partners during their visit, thus arising the concern of an increasing number of new HIV infections. Our aim was to stablish the HIV prevalence in a sample of men attending to a gay sauna in the city of Cordoba, Argentina.

**Material and methods**

Rapid HIV tests (Alere Determine HIV 1/2) were offered to all men who attended to a gay sauna during a specific day every two weeks, from March to June, and from September to November, 2017, accounting for a total of 15 interventions. Counselling was given to all people and for positive cases established the appropriate referral. The information was collected in an Ad Hoc database and descriptive statistics were performed.

**Results**

During the two periods assessed, an estimate of at least 450 men attending to the sauna was reached out, and 53 of them accepted to be tested for HIV. Age range: from 15 to over 49 years old. A total of 10 cases were HIV positive (18.9%), three of them had had a previous negative test. It was the first HIV test for 34 men (64.1%), seven of them were positive (20.6%). Unprotected sex (no condom use): all the 10 HIV positive cases, and 24 out of 34 negative cases. Seven HIV positive men recognized that they had sexual intercourses with women as well.

**Conclusions**

Despite the small number of men tested, the HIV prevalence was high, and more remarkable in those subjects with high risk behaviour never tested for HIV before. Hence a larger study including several venues has to be performed, and sauna‐based interventions emphasizing safer sex and frequent testing need to be implemented.

## P031

Abstract Withdrawn

## P032

Abstract Withdrawn

## P033

### Co‐occurring psychosocial problems in transgender women living with HIV enrolled in a clinical trial in Argentina

**I Aristegui^1^, P Radusky^1^, V Zalazar^1^, C Frola^1^, N Cardozo^1^, A Gun^1^, E Fojo^1^, M Romero^2^, P Cahn^1^ and O Sued^1^**

^1^Research Department, Fundación Huesped, Buenos Aires, Argentina. ^2^Secretaría General, Asociación de Travestis Transexuales y Transgéneros de Argentina, Buenos Aires, Argentina

**Background**

Co‐occurring health problems, including poor mental health, stigma and discrimination, and economic hardship are syndemic factors that cumulatively determine HIV vulnerability in transgender women (TGW). Despite universal access to public health in Argentina, and the Gender Identity Law passed in 2012, TGW continue being one of the most vulnerable communities. Estimated life expectancy is 35 years old and HIV prevalence is 34.1%. The objective of our research is to evaluate the impact of co‐occurring psycho‐social factors in the retention and adherence of TGW in HIV treatment.

**Materials and methods**

This abstract shows preliminary analysis of baseline social and psychological assessments of TGW living with HIV enrolled in a clinical trial. Frequencies and bivariate correlations were calculated with SPSS 24.

**Results**

The sample consisted of 35 TGW (median age 29; IQR: 26 to 36), 49.9% has advanced HIV infection (43% CD4<350, 20% CD4<200). 68.6% are currently involved in survival sex work and 57% have used drugs in the last year. 60% have an incomplete secondary education, and a third (34%) live in community housing rooms. The higher the Gender Identity Stigmatization (GIS), the higher the suicidal ideation (r = 0.57), depression (r = 0.55), and anxiety (r = 0.53) and the lower the quality of life (r = ‐34). GIS is also associated with maladaptive personality traits (DSM‐5): more negative affectivity (r = 0.43), psychoticism (r = 0.39) and detachment (r = 0.36). Approximately half of the sample (48.6%) experience clinical levels of negative affectivity (negative emotions and poor self‐concept).

**Conclusions**

Results of this baseline analysis show that half of TGW initiate treatment with advanced HIV infection and report co‐occurrences between GIS and negative psycho‐social outcomes. Since these factors might negatively impact in retention and adherence of HIV treatment, it is important to consider multi‐component intervention assessing TGW psycho‐social needs as standard of care in clinical settings.

## P034

Abstract Withdrawn

## Models of Care/Scale‐Up of Treatment

## P035

### The continuum of care among very immunosuppressed (less than 100 CD4+ cells) patients in an outpatient clinic in Mexico City: Gaps in diagnosis, linkage and retention in care

**A Piñeirúa‐Menendez^1^, M Del Hoyo^1^, F Osorno González de León^1^, F Badial Hernández^2^and R Niño‐Vargas^3^**

^1^HIV Clinic, Clínica Especializada Condesa Iztapalapa, Mexico City, Mexico. ^2^Director, Clínica Especializada Condesa, Mexico City, Mexico. ^3^IT, Clínica Especializada Condesa, Mexico City, Mexico

**Background**

Late diagnosis and late presentation are still prevalent in Latin America region. These diagnosis scenarios have negative impact both in the individual level as well as from the public health perspective.

**Materials and methods**

We included all patients who were seen for first time in our clinic with CD4+ cell counts under 100 cells/mm^3^; and who were either recently diagnosed, naïve to antiretroviral therapy (ART) or without ART for at least three months or more. Patients were classified as late testers (LT) when their first HIV positive test was less than 6 months away from the first medical visit, late presenters (LP) as those who had an HIV positive test 6 months or more time apart from the first medical visit, and chronic intermittent users (CIU) as those who were previously linked to HIV care and on ART at least for one month. We then built an HIV cascade to identify the profile of patients in each diagnosis scenario and compared characteristics among groups.

**Results**

In the last two years, out of the 2000 patients currently on care at our clinic, 164 (8.2%) patients had complete data and met the inclusion criteria. Of those, 117 (71.3%) were classified as LT, 24 (14.6%) as LP and 23 (14.8%) as CIU. Mean age of participants was 36.6 years. Although women represent only 15.2% of the sample, the majority of them were classified as late testers (88%). There was no association between sexual preference, educational level and drug use with the different outcomes. Previous access to social security was strongly associated to chronic intermittent users (*p* 0.0017). By now, 7.9% of the patients are LTFU (defined as more than three months without pill collection or >12 months without medical visits), and 3.6% died. Deaths were due to AIDS defining conditions in all cases (Table 1.).

**Conclusions**

In our sample, late testing represents 70% of all patients with less than 100 CD4+ cell count at first medical visit. However, 1/3 of our sample is comprised of patients with previous diagnosis, who were either never linked to care or not retained. Lack of continuity in care between different healthcare systems seems to be an important factor for ARV/ HIV care discontinuation. Increasing diagnosis strategies might be the first step to improving this continuum; however, more studies to better understand the reasons that explain these different scenarios are needed.

**Table:** Abstract P035–Table 1. Distribution of patients according to the different categories of presentation

N (%)	LT^a^ (n = 117)	LP^b^ (n = 24)	CIU^c^ (n = 23)	*p*
**Age**, mean	36.5	36.5	36.6	0.9
Gender				0.32
Male	93 (79.4)	23 (95.8)	20 (86.9)	
Women	22 (19.6)	0	3 (13)	
TGW	2 (1.7)	1 (4.1)	0	
Sexual preference				
MSM	58 (49.6)	18 (75)	10 (43.4)	0.17
Bisexual	16 (13.6)	2 (8.3)	2 (8.6)	
Heterosexual	41 (35)	4 (16.6)	10 (43.4)	
Heterosexual females	22	0	3	
Heterosexual males	20	4	7	
No info	2 (1.7)	‐	1 (4.3)	
Educational level				
Elementary school or less	21 (17.9)	3 (12.5)	3 (13)	0.30
Middle school/High school	62 (70.9)	12 (50)	17 (73.9)	
College/higher	33 (28.2)	9 (37.5)	3 (13)	
Missing	1 (1.7)	‐	0	
**History of imprisonment**	1 (0.8)	1 (4.1)	0	
**Previous access to social security**	11 (9.4)	4 (16.6)	10 (43.4)	.00017
**Drug use**	26 (22.2)	6 (25)	5 (21.7)	0.95
IV drug use	0	0	0	
**Mean CD4** + **at first visit**	49	48	41	
ARV therapy started				
TDF + FTC + EFV	90 (76.9)	21 (87.5)	8 (34.7)	
KXA + EFV	2 (1.7)	0	1 (4.3)	
TDF + FTC + RAL	7 (5.9)	1 (4.1)	0	
TDF + FTC + DTG	6 (5.1)	0	1 (4.3)	
ABD + 3TC + DTG	2 (1.7)	0	0	
PI based regimen	10 (8.5)	2 (8.3)	13 (56.5)	
Current Status				
Alive, in care, virologically supressed	79 (67.5)	18 (75)	13 (56.5)	
Alive, in care, virological failure	2 (1.7)	1 (4.1)	3 (13)	
Alive, in care, low level viremia	10 (8.5)	1 (4.1)	3 (13)	
Alive, in care for < 6 months	8 (6.8)	2 (8.3)	0	
In care at other facility	4 (3.4)	0	1 (4.3)	
LTFU	10 (8.5)	1 (4.1)	2 (8.6)	
Deaths	4 (3.4)	1 (4.1)	1 (4.3)	

a LT, late testers.

b LP: late presenters.

c CIU, Chronic intermittent users.

## P036

### Prolonged hospital stays and associated factors in patients receiving care in a HIV/AIDS clinic in Mexico City

**R Lara‐Medrano, A Camiro‐Zúñiga, Y Caro‐Vega, B Crabtree‐Ramírez, J Sierra‐Madero and P Belaunzarán‐Zamudio**

Infectology Department, Instituto Nacional de Ciencias Médicas y Nutrición, Salvador Zubirán, Mexico City, Mexico

**Background**

Assessing hospitalizations and length of stay among HIV‐infected patients provides information on morbidity and healthcare utilization, which are essential to evaluate healthcare provision and project costs. We sought to assess hospitalization rates and length of stay, and risk factors associated with prolonged hospitalization in a cohort of HIV‐infected patients from Mexico City.

**Materials and methods**

We assessed hospital admissions of patients with HIV from January 2000 to November 2017 at a tertiary care hospital. We estimated hospitalization incidence and classified hospitalizations in two groups according to length of stay in prolonged (>21 days) or regular stay (<21 days). We compared demographic, clinical and laboratory characteristics at hospitalization using logistic regression models to identify the risk of prolonged stay (PSH) as a function of sex, age, updated CD4 counts and viral suppression (HIV RNA<400 copies/μl), and use of ART. We compared the distribution of reason for hospitalization between groups using *X2*.

**Results**

There were 3421 patients contributing to 20,397 cumulative years of follow‐up. There were 2581 hospitalizations accounting for an incidence of 12.6 hospitalizations per/100patients‐year. There were 295 (11.42%) PSH. Patients with PSH had a higher proportion of subjects with CD4 <350 cells/ml (91.6% vs. 83.4%, *p* < 0.001), fewer patients receiving ART (50.8% vs. 59.9%, *p* = 0.003), and were more frequently hospitalized due to AIDS‐related events (80.0% vs. 69.3%, *p*=< 0.001) in comparison with regular stay. In patients with regular stay, non‐AIDS events were more frequent (6.1% vs. 11.4%, *p* = 0.006) (Table 1).

**Table:** Abstract P036–Table 1. Characteristics of hospitalized patients analysed across periods of 4 years. Reported values correspond to frequency (percentage), unless otherwise specified

Category (n = 2581)	2000 to 2004 (n = 630)	2005 to 2008 (n = 476)	2009 to 2012 (n = 771)	2013 to 2017 (n = 700)
Male sex	523 (83.02%)	424 (89.08%)	656 (85.08%)	611 (87.29%)
Age (years) – Median (IQR)	36.5 (30.3 to 44.6)	37.8 (29.5 to 46.2)	38.0 (30.8 to 46.5)	37.8 (31.3 to 49.2)
Socio‐economical level				
Low	455 (80.82)	355 (81.99)	564 (83.43)	528 (85.99)
Middle	74 (13.14)	63 (14.55)	89 (13.17)	69 (11.24)
High	34 (6.04)	15 (3.46)	23 (3.4)	17 (2.77)
Transmission route				
Hetrosexual	216 (38.64)	138 (32.55)	198 (30.51)	165 (27.41)
MSM	325 (58.13)	280 (66.04)	434 (66.87)	414 (68.77)
UDIV	0	0	1 (0.15)	1 (0.17)
Other	18 (3.22)	6 (1.42)	16 (2.47)	22 (3.65)
CD4 count (cells/ml)				
>500	42 (8.03)	25 (5.49)	38 (5.26)	70 (10.48)
350 to 500	33 (6.31)	26 (5.71)	53 (7.33)	79 (11.83)
<350	448 (85.66)	404 (88.79)	87 (87.41)	519 (77.69)
CV log (copies/ml) – Mean (SD)	3.46 +‐ 1.55	3.47 +‐ 1.51	3.55 +‐ 1.83	3.24 +‐ 1.83
<=400	208 (33.02)	172 (36.13)	332 (43.06)	360 (51.43)
>400	422 (66.98)	204 (63.87)	439 (56.94)	340 (48.57)
On ART at hospitalization	358 (56.83)	241 (50.63)	441 (57.20)	478 (68.29)
Time on ART (days) – Median (IQR)	0 (0 to 638)	0 (0 to 199)	0 (0 to 279)	116 (0 to 1312)
AIDS‐related cause for hospitalization	427 (67.78%)	351 (73.74)	555 (71.98)	487 (69.57)
Prolonged hospitalizations	56 (8.89)	65 (13.66)	97 (12.58)	76 (10.86)
First time hospitalizations	362 (57.46)	246 (51.68)	370 (47.99)	343 (49.0)
Early readmissions	82 (13.02%)	80 (16.81)	173 (22.44)	140 (20.0)
In‐hospital death	3 (0.48)	10 (2.1)	7 (0.91)	9 (1.29)

The proportion of hospitalizations due to AIDS‐related events (70%), and of PHS (11%) did not change over time (see Table 2).

**Table:** Abstract P036–Table 2. Proportion of hospitalizations due to AIDS‐related events and of PHS

Category	Regular stay (n = 2285)	Prolonged length of stay (n = 295)	*p* value
Male sex	1967 (86.05%)	250 (84.75%)	0.773
Age (years) ‐ Median (IQR)	37.6 (30.4 to 47.1)	36.6 (31.2 to 44.1)	0.253
Low socio‐economical status	1691 (83.1%)	212 (83.79%)	0.679
MSM (HIV transmission route)	1286 (64.62%)	169 (68.7%)	0.226
Patients with CD4 count <350 cells/ul	1748 (83.44%)	253 (91.67%)	0.0004
Patients with viral load <400 cells/ml	987 (43.18%)	83 (28.14%)	<0.001
On ART at hospitalization	1371 (59.97%)	150 (50.85%)	0.003
Time on ART (days) ‐ Median (IQR)	17 (0 to 690)	0 (0 to 149)	0.099
AIDS‐related cause for hospitalization	1586 (69.38%)	326 (80%)	<0.001
First time hospitalizations	1140 (49.87%)	185 (62.71%)	<0.001
Early readmission	431 (18.85%)	44 (14.92%)	0.100

Lower CD4 counts at hospitalization (OR=0.99, *p* < 0.006 per unit of CD4 increase, CI 95% = 0.996 to 0.999), and detectable HIV RNA (OR = 1.71, *p* = 0.002, CI95% = 1.209 to 2.432) were independently associated with PHS (Figure 1).

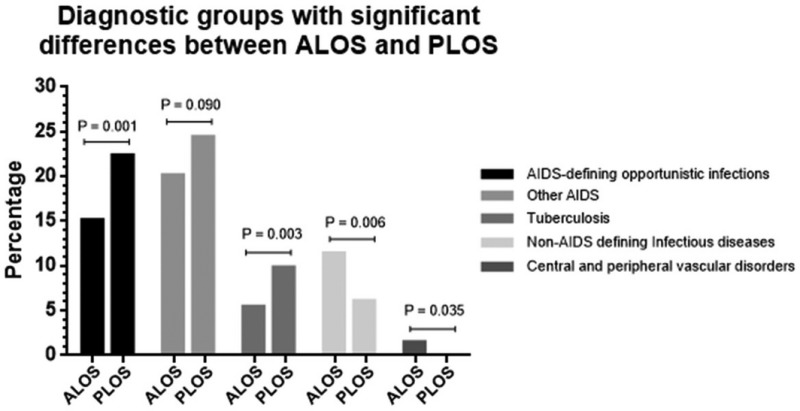

**Abstract P036–Figure 1. Diagnostic groups with significant differences between ALOS and PLOS.**

**Conclusions**

We observed an incidence of hospitalizations similar to previous reports in Latin America. We also observed that 1 in 10 hospitalizations lasted more than 21 days. We identified that AIDS‐related events were the most frequent cause of hospitalization and this did not change over time. Low CD4 and detectable HIV RNA were the main risk factors associated to prolonged hospital stay. Hospitalization rates associated to advanced HIV disease continue to be high in our setting and this seems to have an impact in length of hospitalization.

## P037

### Vaccine coverage in adults living with HIV and receiving care in a tertiary care centre in Mexico City, 2005–2016

**M Mendez‐Roldan, A Olmedo‐Reneaum, J Cano‐Torres, A Camiro‐Zuñiga, Y Caro‐Vega, A Galindo‐Fraga, M Huertas‐Jimenez, J Sierra‐Madero and P Belaunzaran‐Zamudio**

Infectious Diseases, Instituto Nacional de Ciencias Médicas y Nutrición Salvador Zubirán, Mexico City, Mexico

**Background**

Vaccines are essential preventive interventions for people living with HIV (PLWHIV). Vaccine coverage among adults in Mexico is sub‐optimal, but no data about adult PLWHIV is available. We investigated vaccine coverage in PLWHIV receiving care at our clinic during 2005 to 2016.

**Materials and methods**

We conducted an observational, cross sectional analysis on cohort data of patients registered for care for HIV at the HIV/AIDS Clinic of the Instituto Nacional de Ciencias Medicas y Nutrición Salvador Zubirán in Mexico City, during 2005 to 2016. The clinic located in a tertiary care, referral hospital. We collected data on pneumococcal, tetanus, Hepatitis B, measles and influenza vaccines. We used two measurements of vaccine coverage: self‐report of life‐long vaccine application in a sample of patients actively receiving care during 2017, and vaccine application registries from 2005 to 2016 from the Adult Immunization Clinic (UIA). We also explored reasons for lack of vaccine application and attitudes toward vaccines.

**Results**

We included data of 2464 adults. Of these 394 (16%) received influenza vaccine in the season previous to their last visit; 856 (34.7%) received pneumococcal vaccine; 869 (35.2%) HBV, and 264 (10.7%) Tetanus. Of 693 interviewed patients between March and June 2017, 461 (68%) self‐reported receiving the influenza vaccine in the previous season, 577 (85%) tetanus in their life‐time, 544(80%) pneumococcal, and 463 (68.3%) measles and 282 (42%) received all of them. The most frequently reason for not receiving influenza vaccine was “not wanting to get the vaccine” (25%) and having forgotten to apply it (25%). Overall, attitudes towards vaccines were positive: 90% considered vaccines important for health, 91% regarded vaccines safe or very safe, 85% regarded information about vaccines provided by their physician as reliable or very reliable; and 92% perceived vaccines as a means to protect other members in their community. We identified fear of adverse events (92% of respondents were worried about these) and cost (38% answered cost as an important or very important reason for not applying the prescribed vaccines) as potential barriers for vaccine application.

**Conclusions**

Vaccine coverage among adults receiving care for HIV in our clinic is suboptimal for influenza, pneumococcal and HBV. While most patients have positive attitudes toward vaccines, forgetting or plainly refusing vaccine applications were the most frequent reasons for not getting immunized. Cost and fear of adverse events are potential barriers for vaccination. Interventions to increase vaccine uptake in our clinic should facilitate access and improve education about adverse events.

## P038

### HIV clinical monitoring in the southern region of Brazil

**G Mendes Pereira, R Abrahão Ribeiro and F Alvarenga Pinto**

Departamento de Vigilância, Prevenção e Controle, Ministry of Health, Brasília, Brazil

**Background**

In Brazil, clinical cascade monitoring was implemented to evaluate HIV policies related to care and treatment. The cascade contributes to assess also the 90‐90‐90 targets (90% of people living with HIV/AIDS (PLWHA) diagnosed; 90% of diagnosed people on antiretroviral therapy (ART); and 90% of people on ART with suppressed viral load (VL)) adopted in the country. In 2013, the Ministry of Health recommended dispensing ART for all diagnosed HIV+ individuals in order to reach fully suppressed VL and epidemic reduction. However, important hotspots can still be found, mainly in the southern region of the country. This study evaluates the cascade in order to identify the main gaps negatively affecting viral suppression among PLWHA.

**Materials and methods**

Individual case data were obtained from crossing the two official databases related to ART dispensation and laboratory results of CD4, CD8 and VL counts, conducted by the public health system. The data for PLWHA who had at least one VL or CD4 counts or ART dispensation in 2016 were analysed. The cascade phases were estimated: 1. Linked patients (those who had at least one VL or CD4 counts, or ART dispensation); 2. Retention in care (defined as those who had at least two VL or CD4 counts, or had an ART dispensation in the last 100 days of 2016); 3. On ART (defined as having at least one ART dispensation in the last 100 days of 2016); and 4. VL suppression (VL <1000 copies/ml).

**Results**

It is estimated that there are approximately 110,000 people living with HIV in the state of Rio Grande do Sul. Of these, only 73,000 (66.4%) have been diagnosed; out of this diagnosed population, 52,000 (71.9%) are on antiretroviral therapy; and 47,000 (90.1%) of these already have suppressed viral loads. In order to achieve the 90‐90‐90 goals, Rio Grande do Sul needs to diagnose over 23.6% of PLHIV and ensure that over 18.1% of the diagnosed patients start antiretroviral therapy.

**Conclusions**

Findings show that the low number of diagnosed cases is a frailty in the state of Rio Grande do Sul, and indicate that HIV testing is not being widely offered to the population. This means missing an important opportunity to obtain the early diagnosis of HIV+ people in the state. The levels of ART coverage are also low, considering that only 60% of estimated cases have been diagnosed and, out of these, only 71% are on ARV.

## P039

### Advances in the management model for the provision of HIV genotyping services in Brazil

**I Kohiyama, J Alonso, R Pinho, M Villares and A Pires**

Department of STIs, HIV/AIDS and Viral Hepatitis, Ministry of Health, Brasília, Brazil

**Background**

Up to 2015 Brazil's Ministry of Health (MoH) purchased genotyping tests and distributed them through a national laboratory network (Renageno). This involved the costs of logistical arrangements for kits and samples, laboratory equipment maintenance and continuous human resources training in the laboratory network. However, since 2016 testing has been contracted out to a private‐sector laboratory service which includes centralized testing, samples collection in 708 collection points throughout the country and the online release of test results. This study compares the features of these two schemes for acquiring HIV genotyping tests.

**Materials and methods**

Data were collected from the Genotyping Control System (Sisgeno) database from 2015 to 2017 and a comparative analysis of the variables performed.

**Results**

Under the old system, when the MoH was buying and distributing the kits, public laboratory professionals were responsible for evaluating the test requests to check if they complied with the criteria established in the relevant clinical protocol. This procedure, and its outcomes, lacked necessary rigor and generated unnecessary costs. However, after the outsourced service was hired, the private laboratory rejected all the non‐compliant requests, which represented 17% of total requests and generating an approximate saving of US$300,000 to the public purse in 2017 alone. Moreover, the average time of results‐release in the old input purchasing system (49 days in 2015) fell to 11 days in 2017, thus optimizing patients´ clinical management. Another important advance related to antiretroviral resistance monitoring was the increased number of results released: from 8080 in 2015 to 12,265 in 2017. Finally, the unit value of tests under the new scheme is now 40% less than for those purchased previously by the MoH.

**Conclusions**

The study showed that outsourcing genotyping testing offers a number of advantages such as standardized testing, a single laboratory for the MoH to ensure quality control, lower costs overall (e.g. price of reagents, logistics costs), reduced delivery time of test results, and an end to unnecessary testing. The outsourced service approach has also boosted access to testing, thereby enabling patients from all Brazil´s regions to receive equal treatment ‐ an important step forward in Brazil´s efforts to achieve the 90‐90‐90 goal.

## P040

### Reduction of errors during the pre‐analytical process for the collection of HIV samples in Mexican centres: a pilot study of a new software strategy implementation

**R Rodriguez‐Diaz, A Ramos‐Hinojosa, A Orta‐Resendiz, L Angulo‐Medina, J Gomez‐Cuellar, J Sierra‐Madero and L Soto‐Ramirez**

Infectious Diseases, Instituton Nacional de Ciencias Medicas y Nutricion Salvador Zubiran, Mexico City, Mexico

**Background**

For more than thirty years our attempts have been focused in access to HIV treatment, diagnosis, prevention, universal care and others. Laboratory testing includes a highly complex process commonly called Total Testing Process (TTP), in which errors remain a big problem for healthcare services quality standards and patient safety. The majority of errors in TTP (68 to 71%) still remain within pre‐analytical phase of the process. The purpose of this study was to develop and test a new software strategy for the prevention of errors in pre‐analytical phase during the collection of samples.

**Materials and methods**

Based on a previous error monitoring phase during the period between March and July 2017, we identified the errors in the identification of blood samples (for CD4 + count and viral load testing) from four centres in Mexico (at these 4 cities, Queretaro, Reynosa, Tampico and Tlaxcala) that belong to the national HIV programme; then, we developed a brand new software with the design of a comprehensive strategy to assign and automate sample identification (including: training, hardware, fast internet access and label printers if needed.) After implementation, we measured and monitor type and quantity of errors within these centres during August to December 2017. After this period, we analysed the collected data and evaluated the proportion of errors.

**Results**

The previous monitoring phase mean error variation detected during the identification of samples was 10.23% (range 2.3 to 21.1%, n = 2944 samples); after implementation the strategy on participants centres the rate of error decrees to 7.89%, n = 3086 patients. During this process we identified two error types; in the label tubes ID´s and in the laboratory test request forms (4.35% and 3.54% respectively). When we excluded the data from two centres who decided not to use the software, the proportion of error felt down to 4.77%. The highest proportion of errors that we found was during the printing of labels phase.

**Conclusions**

This software system implementation strategy showed a 2.41% reduction of error among all centres. One of the centres Tampico, with the highest proportion of error (21.1%) showed a reduction of 13.59%. Even the centre in Tlaxcala, that had the lowest proportion of error (2.32%) before strategy implementation, made a reduction in its proportion error of 0.38%. This software strategy will be extended to other collecting‐samples centres to improve the quality of HIV healthcare services.

## P041

### The ‘no‐show’ patients in HIV clinical care

**M Sandoval, M Kundro and M Losso**

HIV Unit, Hospital General de Agudos JM Ramos Mejía, Buenos Aires, Argentina

**Background**

In the last years, advances in the diagnosis and treatment of people living with HIV have significantly improved survival. However, many patients do not completely benefit due to poor retention in medical care and treatment adherence. Missed clinic visits constitute a healthcare related issue, as they have been associated with worse clinical outcomes among HIV‐infected patients. We examined the proportion and factors associated with missed HIV medical visits in a large public hospital in Buenos Aires.

**Materials and methods**

We performed a cross‐sectional analysis including all subjects who took an HIV‐care appointment during a five‐month period. “No‐show” individuals were defined as those subjects with two or more scheduled HIV care visit that were not cancelled either by the patient or by the clinic for which the patient did not arrive. We collected patient‐level data on demographic characteristics, immunological and virological status, and calculated the proportion of missed visits.

**Results**

A total of 249 patients (17.2%) of 1.449 subjects who scheduled an appointment during the study period missed two or more clinic visits. Overall, median age was 40 years (IQR 32 to 48), 37.8% were female and 10% transgender women. As much as 23.5% of “no‐show” HIV‐patients interrupted antiretroviral therapy for at least one month in the last year and 26.9% failed to have an undetectable viral load, compared with 12% and 18.5% respectively of patients who did not miss clinic visits. Use of cocaine or marijuana, being transgender women and having a psychiatric disorder were associated with “no‐show” visits. In contrast, patients ≥ 50 years, men who have sex with men and having chronic comorbidities (except for psychiatric disorder or disability) were less likely associated with no‐show visits. Furthermore, having a formal employment, place of residence (Buenos Aires City vs. suburbs) or level of education were not associated with missed visits in this cohort.

**Conclusions**

Missed HIV visits were common in our cohort and were associated with a higher proportion of patients with detectable viral load and treatment interruptions. Additional knowledge is needed to evaluate barriers to appointment compliance and to establish strategies focused on optimal access to care.

## P042

### Contribution of SISCEL/SICLOM systems for PLHA monitoring: CTA/environmental dermatology experience/SES/RS in Brazil

**D Machado Alves, N Both, F Torres de Carvalho, E Alnoch, L Castoldi and G Canterle**

Ambulatório de Dermatologia Sanitária, Secretaria Estadual da Saúde, Porto Alegre, Brazil

**Background**

The HIV/AIDS epidemic in the south of Brazil is of concern, reinforcing the importance of actions among people living with HIV/AIDS (PLHA) at all stages of care (diagnosis, linkage, retention in health service, use of ART and viral load suppression) [1]. Since 2014, the CTA/ADS has been working with PLHA monitoring, as a tool that seeks effectiveness and resolution in linking, backing and networking [2].

**Materials and methods**

In the two initial years, the monitoring took place by telephone (with prior signature of consent), both of the persons linked to the SAE/ADS itself, as well as those referred to Basic Care (AB) and other specialized services. In 2016, the research in the SICLOM/SISCEL systems was incorporated into the monitoring. Currently, monthly identification of positive users, notification of cases, registration in search worksheet (personal data, date of testing, SISCEL/SICLOM inclusion data) is performed. The telephone contact occurs if the user is not inserted into any system within 6 months.

**Results**

The success rate of monitoring was not high. In 2014/2015, it was possible to monitor almost exclusively patients inserted in the SAE/ADS itself. People sent to the network were hard to find. Contact with management also did not generate feedback. The inclusion of information systems completely changed the monitoring situation. In 2016, 191 were diagnosed, 61 of which were inserted into SAE / ADS itself and 26 were already undergoing treatment. Thus, 104 patients referred to AB, other SAEs or hospitals were monitored. Of these, 50% were inserted into SISCEL and/or SICLOM within one‐month post‐diagnosis; 20% were inserted in up to 2 months; 10% within 3 months; 5% within 6 months; and 2% in more than 6 months. In total, 13% are not in any system, without telephone success. Two patients reported that they were being treated, without proving in any information system.

**Conclusion**

The success of monitoring with the inclusion of search in information systems was evident. It is known that CTA teams generally do not have access to these systems, but the experience of CTA/ADS is indicative of this being a good strategy for continued care efficiency. It draws attention to the short time between diagnosis and insertion in the network, which is positive for patients and motivator for the team, who feels more confident in the bonding effort.

**References**

1. Brasil. 2010. Diretrizes para organização e funcionamento dos CTA do Brasil. Brasília. DF

2. Brasil. 2017. HIV/AIDS na Atenção Básica – Cinco passos para a construção de linhas de cuidado para pessoas vivendo com HIV/AIDS. Brasília – DF.

## P043

### Provincial differences in the update status of antiretroviral treatments

**G Soulas, S Maulen, A Biscay, M Marovic, M Mendizabal and P Pardo**

Ministerio de Salud de la Nacion, Buenos Aires, Argentina

**Background**

The Direction of AIDS and STD performs the planning of purchases and logistics of antiretroviral drugs for public hospitals in the country. The public system covers treatment free of charge for 70% of people living with HIV in Argentina (approximately 50,000 patients). Purchases are made annually and must be planned two years in advance. This circumstance makes it necessary to foresee the consumption of each drug available throughout the country and to think prematurely of the incorporation of new treatments to be able to offer, two years after the planning, updated treatments in relation to the national and international consensus on antiretroviral therapy. From these analyses, significant differences were found in the average cost of treatments per person in the different provinces of the country, so it was decided to carry out this study in order to find the relationship between these values and the consumption of certain antiretroviral drugs. The DSyETS, offers the same vademécum to the whole country, and the Argentine society of infectology publishes annually updated antiretroviral treatment consensus, so it seems striking to find great differences in the average cost of treatments per person between different provinces of the country and we suspect that the answer may be in the different degree of updating of the treatments prescribed in these provinces. The objective of this work is then to analyse the degree of updating of antiretroviral prescriptions with respect to the recommendations present in the SADI 2016 to 2017 consensus.

**Materials and methods**

The present is an ecological, retrospective and observational study. The analysis was carried out in 6 provinces of the country, where the inclusion criteria were those that carry out medication orders on a bimonthly basis and that comply regularly with ministerial resolution 769/98. The analysis time of the study is from January 2017 to June 2017 and all the variables were calculated within that period.

**Results**

Cost per patient per month in the different provinces evaluated in dollars: Santa Cruz (USD$156.90), Formosa (USD$85.36), Mendoza (USD$124.55), Chubut (USD$98.17), Neuquén (USD$120.58), Missions (USD$62.57). Updated percentage of antiretroviral treatments (number of people with treatments recommended by the SADI on total treatments): Mendoza (82%), Santa Cruz (89%), Chubut (74%), Neuquen (82%), Formosa (67%), Missions (62%).

**Conclusions**

Based on the results of the present study, we can conclude that the provinces present differences in the degree.

## Non‐AIDS Morbidities and Mortality, and Ageing

## P044

### Low frequency of screening for anal carcinoma in a cohort of adult men receiving care for HIV in a third‐care, referral centre in Mexico City

**L Gomez‐Garcia, P Belaunzarán‐Zamudio, B Crabtree‐Ramírez, J Sierra‐Madero and Y Caro‐Vega**

Infectious Diseases, Instituto Nacional de Ciencias Médicas y Nutrición, Salvador Zubirán, Mexico City, Mexico

**Background**

The risk of anal carcinoma (AC) is 32 to 52‐times higher among HIV+ men who have sex with men (MSM) [1,2,3]. Current screening guidelines recommend anal cytology (PAP) every 1–3 years and high resolution anoscopy (HRA) after abnormal PAP findings [2,4]. There is a dearth of data on screening practices and frequency of AC in Mexican adults receiving care for HIV. We aimed to describe the frequency of AC screening–tests in a single HIV‐clinic in Mexico City and identify factors associated with being screened during 2010 to 2016. We also estimated the prevalence of anal dysplasia.

**Materials and methods**

We studied HIV+ men receiving care during at least one year between 2010 and 2016 at one HIV–Clinic in Mexico City. We describe the frequency of screening tests (anal PAP or HRA) by patient, and the prevalence of dysplasia. Lesions were classified as: 1. High grade (HG), if PAP reported atypical squamous cells but cannot exclude high‐grade (ASC‐H), high‐grade anal intraepithelial neoplasia (HGAIN), carcinoma “in situ” (Cis) or anal carcinoma (AC); 2. ASCUS, if atypical squamous cells of uncertain significance; and 3. LGAIN if low grade anal intraepithelial lesion. We retrieved socio‐demographic and clinical variables and fit logistic regression models to identify factors associated to the probability of being screened.

**Results**

We followed 1729 patients for 5.49 years (SD 1.88). Patients received 0.16 (SD 0.209) screening‐tests per year; 894 (51.70%) were screened at least once, accounting for 0.31 (SD 0.191) screening‐tests per year. The proportion of patients with a screening‐test increased from 0.97% (n = 11) in 2010 to 28.56% (n = 423) in 2016, (*p *< 0.001). The mean of CD4 count at AC screening‐test was 530 (SD 263). Being naive to ART (OR 1.44, *p* = 0.007), enrolled after 2008 (OR 1.52, *p* = 0.001), and >9 years of education (OR 1.39, *p* = 0.040) were associated with an increased probability of being screened. During follow‐up there were 510 (57.27%) patients diagnosed with any type of dysplasia, of whom 435 (85.29%) had LGAIN; 69 (13.52%) HG: 1 AC, 12 Cis, 54 HGAIN and 2 ASC‐H; 6 (1.17%) ASCUS. Three‐hundred three (59.26%) had more than one screening test.

**Conclusions**

The frequency of screening for AC is low among HIV+ MSM receiving care in our center. HPV‐associated dysplasias are frequent, but most of these are LGAIN. Interventions to improve AC screening practices are needed to avoid progression to AC.

**References**

1. Shiels, M., Pfeiffer, R., Chaturvedi, A., Kreimer, A. and Engels, E. (2012). Impact of the HIV Epidemic on the Incidence Rates of Anal Cancer in the United States. JNCI: Journal of the National Cancer Institute, 104(20), pp.1591‐1598.

2. Leeds, I. (2016). Anal cancer and intraepithelial neoplasia screening: A review. World Journal of Gastrointestinal Surgery, 8(1), p.41.

3. Mathews, W., Agmas, W., Cachay, E., Cosman, B. and Jackson, C. (2014). Natural History of Anal Dysplasia in an HIV‐Infected Clinical Care Cohort: Estimates Using Multi‐State Markov Modeling. PLoS ONE, 9(8), p.e104116.

4. EACS Guidelines version 9.0, October 2017

## P045

### Evaluation of cardiovascular risk factors in HIV patients in a public hospital

**S Martini, A Falak, A Aguilar, O Sued, P Cahn and H Perez**

Hospital Fernandez, Buenos Aires, Argentina

**Background**

Since the introduction of antiretroviral treatment, both expectation and quality of life of HIV patients have improved to the level of being similar in certain groups to that of the general population; however, different studies show an increase in cardiovascular disease in these patients, represent 8 to 22% of deaths. Although the impact of HIV and its treatment on the aging of patients with HIV is well known, as well as that cardiovascular risk factors (CVRF) are more prevalent in this population, data on these factors in HIV patients is scarce in Argentina. A cross‐sectional study was conducted in the population of HIV patients who were in follow‐up in the Public Hospital of Buenos Aires in order to assess cardiovascular risk by means of Framingham scores at 10 and 30 years in that population.

**Materials and methods**

Cross‐sectional observational study by means of a questionnaire, physical examination and laboratory data to patients over 18 years of age who are HIV positive and who continue in a public hospital of the city of Buenos Aires for one year.

**Results**

We included 272 individuals (64.2% men) with a median age of 43 years (35 to 50), being 27.2% older than 50 years, having a median‐years since the diagnosis of HIV of 10 years (4 to 16), 87% being found in ART, 81% with viral load HIV <50 copies / ml, with a median CD4 of 539 cells / ml (326 to 770). A 10‐year high cardiovascular risk was observed in 18.2% (24.1% in men and 8.24% in women), a 30‐year high cardiovascular risk in 19.6% (24.4% in men and 9.72% in women) and a risk of coronary heart disease at 10 years very high in 27.9% (25.5% in men and 32.3% in women), hypercholesterolemia 48.3%, low HDL 43.4%, TBQ 39.4%, HT 20%, hypertriglyceridemia 54.5%, sedentary lifestyle 47%, overweight 25% and metabolic syndrome 28.4%. Only 19.4% have no CVRF and 51% have 2 or more CVRF. 45% with hypercholesterolemia and 41% with DBT were diagnosed in the study.

**Conclusions**

Considerable proportion of patients over 50 years. High prevalence in both sexes of modifiable CVRF (TBQ, hypercholesterolemia, low‐HDL, hypertriglyceridemia, sedentary lifestyle, overweight, metabolic syndrome and hypovitaminosis D). High proportion of patients with 2 or more CVRF. High prevalence of patients with high cardiovascular risk at 10 and 30 years. High prevalence of patients with very high risk of coronary heart disease at 10 years. Sub‐diagnosis of cardiovascular risk factors

**References**

1. Fedele F, Bruno N, Mancone M 2011 Cardiovascular risk factors and HIV disease. AIDS Rev 13:119‐129

2. Soliman E, Sharma S, Arasteh K, Wohl D, Achhra A, Tambussi G, O'Connor J, Stein J, Duprez D, Neaton J, Phillips A 2015 Baseline cardiovascular risk in the INSIGHT Strategic Timing of AntiRetroviral Treatment (START) trial. HIV Med 16 Suppl S1:46‐54

3. Stein H 2010 Evaluating and managing cardiovascular disease risk factors in HIV‐infected patients. Top HIV Med 18:164‐168

4. Stein JH 2012 Cardiovascular risk and dyslipidemia management in HIV‐infected patients. Top Antivir Med 20:129‐133; quiz 123‐124

5. Prasad P, Kochhar A 2015 Interplay of vitamin D and metabolic syndrome: A review. Diabetes Metab Syndr

6. Grinspoon SK 2014 Cardiovascular disease in HIV: traditional and nontraditional

## P046

### In which geographic areas should actions be targeted to reduce HIV/AIDS mortality in Mexico? An analysis of this magnitude, distribution and trends by Jurisdicción Sanitaria, 1990–2015

**E Bravo‐García^1^, C Magis‐Rodríguez^2^, M Palacios‐Martínez^3^ and N Guarneros‐Soto^3^**

^1^Directorate, CENSIDA, Mexico City, Mexico. ^2^Integral Care, CENSIDA, Mexico City, Mexico. ^3^INSP, Mexico City, Mexico

**Background**

In 1997, people living with HIV who had social security in Mexico, began to receive HAART, which had significantly reduced mortality in other countries. In 2003, free and universal access to TARAA was adopted as a national public health policy. However, mortality due to HIV/AIDS did not decrease as expected, with important differences between the Mexican states. Strategies to reduce mortality should be focused on specific areas. The *Jurisdicciones Sanitarias* (JS) are the structures of the State Health Services that must coordinate the execution of the actions of prevention and control of HIV/AIDS. 

**Materials and methods**

Information on deaths due to HIV/AIDS was obtained from INEGI. For the calculation of crude and standardized rates, the official population estimates of CONAPO were used. The JoinPoint regression model was used to analyse epidemiological trends.

**Results**

The magnitude, distribution and trends of HIV/AIDS mortality in Mexico by JS were analysed. The 25 JS with higher rates of HIV/AIDS mortality were identified and their epidemiological trends were analysed. Although they have only 11% of the populations of the country, they account for 28.6% of the total deaths due to HIV/AIDS. They have a standardized mortality rate that is at least twice the national rate, and among them, seven JS have a rate three or more times higher. The highest average annual mortality rates were observed in Tonalá, Chiapas (14.4 per 100,000 inhabitants), Veracruz, Veracruz (14.3 per 100,000), Carmen, Campeche (13.7 per 100,000), Centla, Tabasco (13.5 per 100,000 inhabitants), Cosamaloapan, Veracruz (13.3 per 100,000), Coatzacoalcos, Veracruz (13.3 per 100,000 inhabitants and Cárdenas, Tabasco (11.6 per 100,000 inhabitants). These 25 JS are located mainly in coastal areas, tourist sites, migration corridors or border areas of the country (Figure 1).

The most recent trend estimated by JoinPoin regression model, shows that in 9/25 JS HIV/AIDS mortality increased, 8/25 decreased and 8/25 there was no change. (Table 1).

**Table:** Abstract P046–Table 1. Most recent trend in the 25 Jurisdicciones Sanitarias with highest HIV/AIDS mortality. Mexico, 1990 to 2015

Order	Jurisdicción Sanitaria	HIV/AIDS standardized mortality rate (2010 to 2015)	Last trend	APC	CI 95%	Statistic Test (t)	Prob > t	Most recent trend
1	Tonalá, Chis.^	14.4	1995 to 2015	4.4^	[1.8 to 7.1]	0	0	Increase
2	Veracruz, Ver.^	14.3	2010 to 2015	‐6.6^	[‐10.5 to ‐2.6]	0	0	Decrease
3	Carmen, Camp.^	13.7	1990 to 2015	5.7^	[3.6 to ‐ 7.8]	0	0	Increase
4	Centla, Tab.^	13.5	2009 to 2015	‐11.0^	[‐20.2 to ‐.08]	0	0	Decrease
5	Cosamaloapan, Ver.^	13.3	2006 to 2015	‐7.1^	[‐11.7 to ‐2.2]	0	0	Decrease
6	Coatzacoalcos, Ver.*	12.9	2003 to 2015	1.5^	[0.1 to 2.8]	0	0	Increase
7	Cárdenas, Tab.*	11.6	2009 to 2015	‐1.2	[‐9 to 7.3]	0.8	0.8	No change
8	Macuspana, Tab.*	11.1	2003 to 2015	‐1.6	[‐5.3 to 2.2]	0.4	0.4	No change
9	Cunduacán, Tab.^	11.0	1994 to 2015	5.6^	[2.2 to 9.2]	0	0	Increase
10	Centro, Tab.*	10.9	2009 to 2015	‐0.5	[‐6.1 to 5.5]	‐0.2	0.9	No change
11	Huimanguillo, Tab.^	10.6	1996 to 2015	5.9^	[2.4 to 9.6]	0	0	Increase
12	Acapulco, Gro.^	10.4	2001 to 2015	‐2.6^	[‐4 to ‐1.3]	0	0	Decrease
13	Comalcalco, Tab.^	9.9	1994 to 2015	3.3^	[1.1 to 5.6]	0	0	Increase
14	Tapachila, Chis.^	9.8	2006 to 2015	‐8.1^	[‐10.9 to ‐5.3]	0	0	Decrease
15	San Andrés Tuxtla, Ve	9.8	2004 to 2015	‐1.6	[‐4.5 to 1.5]	‐1.1	0.3	No change
16	Cancún, Q. Roo^	^9.4	1990 to 2015	2.8^	[1.5 ‐ 4.1]	0	0	Increase
17	Cuauhtémoc, CdMx^	9.3	1995 to 2015	‐5.7^	[‐7.2 to ‐4.1]	0	0	Decrease
18	Nacajuca, Tab.^	9.3	1993 to 2015	3.5^	[0.9 to 6.3]	0	0	Increase
19	Escárcega, Camp.^	9.1	1998 to 2015	5.5^	[1.5 to 9.7]	0	0	Increase
20	Tijuana, BC^	8.4	1996 to 2015	‐3.5^	[‐4.5 to ‐2.5]	0	0	Decrease
21	Poza Rica, Ver.*	8.4	2005 to 2015	‐0.5	[‐3.7 to 2.8]	0.7	0.7	No change
22	Tecomán, Col.*	8.2	1997 to 2015	0.8	[‐2.1 to 3.8]	0.6	0.6	No change
23	Puerto Vallarta, Jal.*	8.2	1993 to 2015	0.3	[‐1.5 to 2.1]	0.3	0.8	No change
24	Villaflores, Chis.^	7.8	2004 to 2015	‐7.3^	[‐13.6 to ‐0.5]	0	0	Decrease
25	Reynosa, Tamps.*	7.7	2008 to 2015	‐3.9	[‐9.9 to 2.5]	‐1.3	0.2	No change

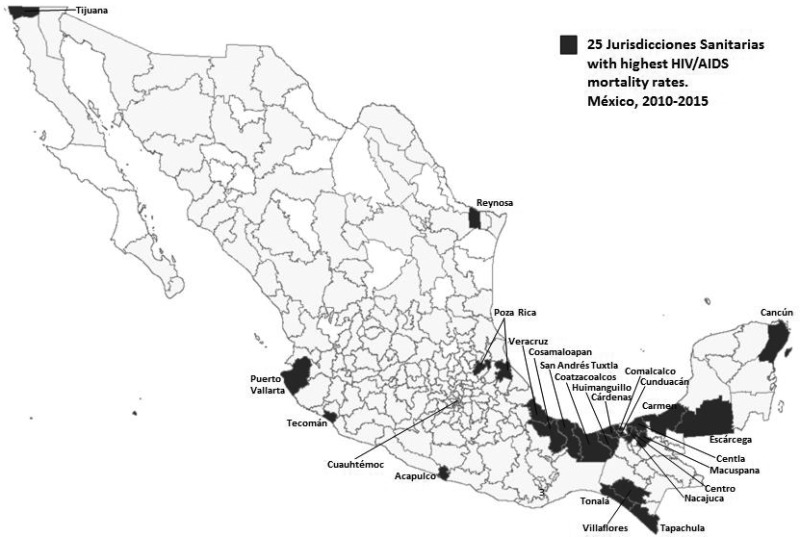

**Abstract P046–Figure 1. 25 Jurisdicciones Sanitarias with highest HIV/AIDS mortality rates. Mexico, 210‐2015.**

**Conclusions**

The 25 geographic and operational areas (JS) were identified, where actions aimed at reducing HIV/AIDS mortality in Mexico should be focused. It is the first study that analyses a health problem (HIV/AIDS mortality) in all *JS* of Mexico.

## Treatment Strategies and Outcomes

## P047

### Safety and efficacy of E/C/F/TAF in HIV‐infected adults on chronic haemodialysis

**J Eron Jr^1^, R Kalayjian^2^, AK Wurapa^3^, J Stephens^4^, C McDonald^5^, A Wilkin^6^, M McKellar^7^, J Custodio^8^, S Jiang^8^, D SenGupta^8^, L Espinoza^8^ and M Das^8^**

^1^University of North Carolina Chapel Hill School of Medicine, Chapel Hill, NC, USA. ^2^MetroHealth Medical Center, Cleveland, OH, Brazil. ^3^Infectious Disease Specialists of Atlanta, Atlanta, GA, USA. ^4^Mercer University School of Medicine, Macon, GA, USA. ^5^Tarrant County Infectious Disease Associates, Fort Worth, TX, USA. ^6^Wake Forest University Health Sciences, Winston‐Salem, NC, USA. ^7^Duke University Medical Center, Durham, NC, USA. ^8^Gilead Sciences Inc, Foster City, CA, USA

**Background**

Elvitegravir (EVG)/cobicistat (COBI)/emtricitabine (FTC)/tenofovir alafenamide (E/C/F/TAF) is approved for use in HIV‐1 infected individuals with mild to moderate chronic kidney disease (estimated glomerular filtration [eGFR] 30 to 69 ml/min). Current HIV treatment for individuals with renal failure on haemodialysis (HD) requires complex regimens with multiple pills. This is the first study to evaluate safety, efficacy and pharmacokinetics (PK) of a daily single‐tablet regimen (STR) in HIV‐infected adults with end stage renal disease (ESRD) on chronic HD.

**Materials and methods**

HIV‐1 infected, virologically suppressed adults with ESRD (eGFR <15 ml/min) on chronic HD for ≥6 months were switched to open‐label E/C/F/TAF 150/150/200/10 mg once daily for 48 weeks (W). Efficacy was assessed as the proportion of participants with HIV‐1 RNA <50 copies (c)/ml (Snapshot algorithm). Maintenance of virologic suppression (<50 c/ml), safety and patient satisfaction (Treatment Satisfaction Questionnaire) were assessed throughout the study. A PK substudy was done at or between W2 and 4. W24 data are presented here and W48 data will be available for the conference.

**Results**

We enrolled 55 participants; median age 51 years (range 23 to 64), 24% female, 82% Black, median time on HD 6 years (range 1 to 17), median CD4 count 515 cells/ml (IQR 387, 672), and 22% Hepatitis C Ab positive, and 27% history of diabetes. At W24, 87% (48/55) had HIV‐1 RNA <50 c/ml. The other seven participants discontinued due to lack of efficacy (n = 1), AE (n = 2), or other reasons not related to efficacy (n = 4). EVG, COBI and TAF PK were consistent with exposures in normal renal function. As expected, exposures of FTC and TFV (metabolite of TAF), which are renally eliminated, were higher v. historical data in normal renal function (Table). Sixteen (29%) participants had Grade (G) 3 or 4 AEs unrelated to study drug; 6 (11%) participants experienced study drug related AEs (all were G1‐2, including nausea in 4). Two participants discontinued E/C/F/TAF due to AEs (allergic pruritis, related; staphylococcal endocarditis, unrelated). The participant with endocarditis died from heart failure after entering hospice. Twenty‐four (44%) participants had G3‐4 laboratory abnormalities, all of which were present at baseline. Seventy‐nine percent of participants felt “much more satisfied” with the STR convenience compared to baseline.

**Conclusions**

Switching to E/C/F/TAF STR maintained virologic suppression at W24, was well tolerated, and more convenient for adults with ESRD on HD (Table 1).

**Table:** Abstract P047–Table 1. Plasma exposure (AUC) after E/C/F/TAF administration in adults with ESRD on chronic HD

	Adults with ESRD on chronic HD (n = 12) Mean (%CV) AUC, ng*h/ml
EVG	14,300 (55)
COBI	10,200 (59)
FTC	62,900 (48)
TAF	232 (53)
TFV	8720 (39)

## P048

### Phase 3, randomised, controlled trial of switching to fixed‐dose bictegravir/emtricitabine/tenofovir alafenamide (B/F/TAF) from boosted protease inhibitor‐based regimens in virologically suppressed adults: Week 48 results

**E Daar^1^, E DeJesus^2^, P Ruane^3^, G Crofoot^4^, C Creticos^5^, J‐M Molina, E Koenig^6^, Y Liu^7^, K Andreatta^8^, H Graham^8^, A Cheng^8^, H Martin^8^, F Silva^8^ and E Quirk^8^**

^1^Harbor‐UCLA Medical Center, Torrance, CA, USA. ^2^Orlando Immunology Center, Orlando, FL, USA. ^3^Ruane Clinical Research Group, Inc, Los Angeles, CA, USA. ^4^The Crofoot Research Center, Houston, TX, USA. ^5^Howard Brown Health Center, Chicago, IL, USA. ^6^Hopital Saint Louis, Paris, France. ^7^Inst.Domin Estudio Virologicos, Santo Domingo, Dominican Republic. ^8^Gilead Sciences Inc, Foster City, CA, USA

**Background**

Boosted protease inhibitor regimens (bPIs) are effective and often used in HIV‐infected individuals with difficulties with adherence, but they can have drug‐drug interactions and GI adverse effects. Bictegravir (B), a novel, potent integrase strand transfer inhibitor with a high barrier to resistance and low potential for drug‐drug interactions, was coformulated with the recommended nucleoside reverse transcriptase inhibitor backbone emtricitabine (FTC)/tenofovir alafenamide (F/TAF) and demonstrated high efficacy and tolerability in randomized studies in treatment‐naïve adults. This randomized Phase 3 study assesses efficacy and safety of switching to B/F/TAF from a multi‐tablet regimen containing a bPI.

**Materials and methods**

HIV‐infected adults suppressed on regimens of boosted atazanavir (ATV) or darunavir (DRV) + abacavir/lamivudine (ABC/3TC) or FTC/tenofovir disoproxil fumarate (TDF), were randomized 1:1 to continue their current bPI regimen or switch to open‐label coformulated B/F/TAF (50/200/25 mg) once daily. Primary endpoint was proportion with HIV‐1 RNA ≥50 copies/ml (c/ml) at W48 (FDA snapshot). Non‐inferiority was assessed through 95.002% confidence intervals (CI) using a margin of 4%. Secondary endpoints included proportion with HIV‐1 RNA <50 c/ml and safety measures at W48.

**Results**

Five hundred and seventy‐seven participants were randomized and treated with B/F/TAF (n = 290) or current bPI regimens (n = 287): 17% women, 26% Black, median age 48 years. Most were receiving a bPI with FTC/TDF (85%) at screening. At W48, switching to B/F/TAF was non‐inferior to continuing bPI with 1.7% in each group having HIV‐1 RNA ≥50 c/ml (difference ‐0.0%; 95.002%CI ‐2.5% to 2.5%, *p* = 1.00); the proportion with HIV‐1 RNA <50 c/ml was 92.1% in B/F/TAF vs 88.9% in bPI. No participant on B/F/TAF developed resistance to study drugs. One participant on DRV/ritonavir + ABC/3TC developed a treatment‐emergent L74V mutation. Incidence of grade 3 or 4 AEs was similar (B/F/TAF 4%, bPI regimens 6%). No renal discontinuations or tubulopathy cases occurred with B/F/TAF.

**Conclusions**

Adults switching to B/F/TAF from a boosted PI maintained high rates of virologic suppression without resistance. B/F/TAF was safe and well tolerated.

## P049

### Superior efficacy of dolutegravir (DTG) plus two nucleoside reverse transcriptase inhibitors (NRTIs) compared with lopinavir/ritonavir (LPV/RTV) plus two NRTIs in second‐line treatment: interim data from the DAWNING study

**M Aboud^1^, R Kaplan^2^, J Lombaard^3^, F Zhang^4^, J Hidalgo^5^, E Mamedova^6^, M Losso^7^, P Chetchotisakd^8^, J Sievers^9^, D Brown^10^, J Hopking^11^, M Underwood^12^, MC Nascimento^9^, M Gartland^12^ and K Smith^12^**

^1^Global Medical Dolutegravir, ViiV Healthcare, Brentford, UK. ^2^Research, Desmond Tutu HIV Foundation, Cape Town, South Africa. ^3^Josha Research, Bloemfontein, South Africa. ^4^Department of Infectious Diseases, Beijing Ditan Hospital, Beijing, China. ^5^VÍA LIBRE, Lima, Peru. ^6^Kiev AIDS Centre, Kiev, Russian Federation. ^7^Infectious Diseases, Hospital J M Ramos Mejía, Buenos Aires, Argentina. ^8^Infectious Diseases, Srinagarind Hospital, Khon Kaen University, Khon Kaen, Thailand. ^9^Clinical Development, ViiV Healthcare, Brentford, UK. ^10^Regional Medical (Dolutegravir), ViiV Healthcare, Abbotsford, Australia. ^11^GlaxoSmithKline, Statistics, Stockley Park, UK. ^12^ViiV Healthcare, Research Triangle Park, USA.

**Background**

DAWNING is a non‐inferiority study conducted to compare a protease inhibitor‐sparing regimen of DTG+2NRTIs with a current WHO‐recommended regimen of LPV/RTV+2NRTIs in HIV‐1 infected subjects failing first‐line therapy of a non‐nucleoside reverse transcriptase inhibitor (NNRTI) + 2 NRTIs (ClinicalTrials.gov: NCT02227238). An Independent Data Monitoring Committee (IDMC) performed periodic reviews of data to protect the ethical and safety interests of subjects.

**Materials and methods**

Adult subjects failing first‐line therapy, with HIV‐1 RNA ≥400 copies(c)/ml, were randomized (1:1, stratified by Baseline plasma HIV‐1 RNA and number of fully active background NRTIs) to 52 weeks of open‐label treatment with DTG or LPV/RTV combined with an investigator‐selected dual NRTI background, including at least one fully active NRTI. An IDMC review was performed, which included data from 98% (612/627 randomized) of subjects through 24 weeks on therapy.

**Results**

At week 24, 78% of subjects on DTG vs. 69% on LPV/RTV achieved HIV‐1 RNA <50 c/ml (adjusted difference 9.6%, 95% CI: 2.7% to 16.4%, *p* = 0.006 for superiority). The difference was primarily driven by lower rates of Snapshot virologic non‐response in the DTG group. The safety profile of DTG+2NRTIs was favourable compared to LPV/RTV+2NRTIs with more drug‐related adverse events (AEs) reported in the LPV/RTV group, mainly due to higher rates of gastrointestinal disorders (Table 1). Following review of week 24 data and large subsets of data from Weeks 36 and 48, the IDMC recommended discontinuation of the LPV/RTV arm due to persistent differences in rates of Snapshot virologic non‐response and protocol‐defined virologic failure (PDVF) favouring the DTG arm.

**Table:** Abstract P049–Table 1. Week 24 outcomes

Week 24 outcomes	DTG (N = 307)	LPV/RTV (N = 305)
Snapshot virologic success	240 (78%)	210 (69%)
Snapshot virologic non‐response	36 (12%)	64 (21%)
Data in window not <50 c/ml	33 (11%)	59 (19%)
Discontinued for other reason while not <50 c/ml or change in ART	3 (1%)	5 (2%)
Snapshot no virologic data	31 (10%)	31 (10%)
Discontinued due to AE or death	5 (2%)	14 (5%)
Discontinued for other reasons or missing data during window but on study	26 (8%)	17 (5%)
PDVF	5/312 (2%)	12/312 (4%)
Drug‐related AEs	45/314 (14%)	107/310 (35%)

**Conclusions**

The IDMC recommended discontinuation of the LPV/RTV arm due to superior efficacy of DTG+2NRTIs and the potential to harm subjects on LPV/RTV based on available data. Final week 24 results of this study will be presented. DAWNING provides important information to help guide second‐line treatment decisions in resource‐limited settings.

## P050

### Monitoring HIV care in Brazil: a national strategy to reduce the HIV treatment gap

**R Vianna Brizolara, A Kolling, J Silva Netto, M Camelo Madeira de Moura, F de Barros Perini and A Schwartz Benzaken**

Ministry of Health, Department of Surveillance, Prevention and Control of Sexually Transmitted Infections, HIV/AIDS and Viral Hepatitis, Brasília ‐DF, Brazil

**Background**

One of the challenge of the Brazilian's HIV treatment programme is monitor and evaluate of clinical and epidemiological aspects of HIV responses to adopt strategies to cover treatment gaps of people living with HIV (PLHIV). To move towards achieving the 90‐90‐90 targets, the country has been implementing the Clinical Monitoring System of People Living with HIV (SIMC); and has been developing efforts in partnership with municipalities and states to scale up early treatment of HIV patients, as soon as possible after they receive diagnosis in the healthcare services. SIMC is an important tool to management process of continuous care of PLHIV and its available without costs to municipalities, states and public health services in Brazil.

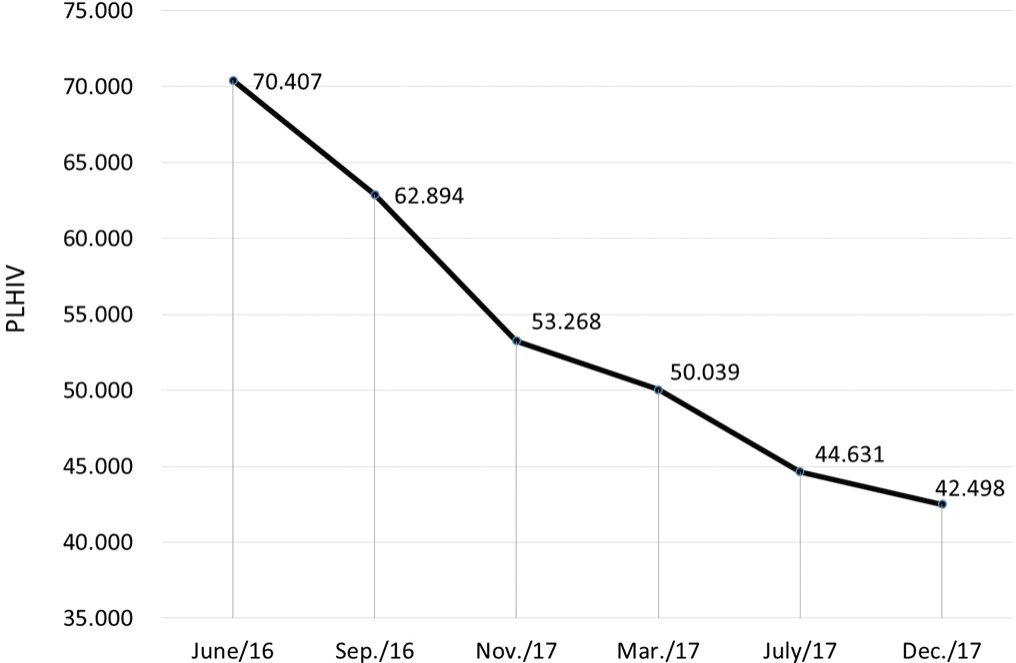

**Abstract P050–Figure 1. Treatment gap in Brazil between June 2016 and December 2017.**

**Materials and methods**

A cross‐sectional study was carried out to describe data related to PLHIV treatment. The results were obtained from SIMC through data crossing of a national laboratory system that shows individual CD4 count and HIV viral load results and a national antiretroviral delivery control system. The period analysed was January 2014 until December 2017. SIMC shows treatment GAP ‐ PLHIV that are not on ART ‐ and monthly monitors the inclusion of these patients into treatment.

**Results**

Between January 2014 and December 2017, 167,650 PLHIV who had not started antiretroviral therapy were identified in SIMC, 136,398 cases were analysed, 115,637 patients were located and started treatment over the period, and 43,603 were not assessed and did not initiated ART. Between June 2016 and December 2017, 27,909 PLHIV left treatment GAP (Figure 1). Currently, SIMC enables to monitor PLHIV without ART, lost to follow up (> 100 days without antiretroviral delivery) or still have detectable viral load despite ART.

**Conclusions**

SIMC represents an important technological innovation, essential to improve quality of healthcare for PLHIV, contributing to actions that may impact the 90‐90‐90 targets for HIV and to eliminate of the AIDS epidemic in Brazil until 2030.

## P051

### Time of initiation of treatment of people living with HIV

**J Uesono^1,2^, H Eri Shimizu^1^, XP Diáz Bermudez^1,3^, E Merchan‐Hamann^1^, F Moreira Rick^2^ and AR Pati Pas^2^**

^1^University of Brasília Graduate Program in Collective Health (UnB), Brasília, Brazil. ^2^Ministry of Health/Secretary of Health Surveillance (MS/SVS), Brasília, Brazil. ^3^Pan American Health Organization (PAHO), Brazil.

**Background**

The current HIV epidemics scenario leads to a need for a new model of care in order to offer proper assistance for people living with HIV (PLWHIV). In this context, primary healthcare (PHC) is an essential service that can provide the conditions to perform this task. This study aims to compare time for antiretroviral therapy (ART) initiation according to different types of health facilities (primary, specialised healthcare and hospital).

**Materials and methods**

We correlated socio‐demographic and clinical factors with the time between first viral load (VL) and/or CD4 count at linkage to care and time for ART initiation. Survival functions were described by Kaplan‐Meier and tested by log‐rank. The Cox model was used to assess the influence of time on the significant variables and to estimate hazard. We used R 2.7 in the statistical analysis and AIC test to determine the best model.

**Results**

A total of 6,963 PLWHIV were included. Overall, 4788 (68.8%) were male; 3378 (48.5%) aged between 25 and 39 years old; 4242 (60%) presented VL≥ 10,000 copies/ml; 2698 (38.7%) presented CD4 count≥500cels/mm^3^; 3203 (46%) were assisted in primary healthcare. Earlier ART initiation was observed among male (84 days, 95% CI = 79 to 82 compared to 108 days, 95% CI = 93 to 123 for female); those presenting VL≥ 10,000 copies/ml (60 days, 95% CI = 57 to 64); CD4 count <200 cels/mm^3^ (52 days, 95% CI = 48 to 57); and those assisted in PHC (78 days, 95% CI 95% = 74 to 84 compared to hospital:132 days, 95% CI = 96 to 178). Adjusted hazard ratio identified “gender” and “age group” as non‐significant variables, and “VL” (VL≥10,000, HR=6.3047, 95% CI = 5.3230 to 7.4674) and “CD4” (CD4 0 to 199, HR = 0.8679, 95% CI = 0.8029 to 0.9381) as the most significant clinical factors associated to earlier ART initiation.

**Conclusion**

This study showed that PHC can be considered a strategic health facility once it is able to provide earlier ART initiation, also playing a crucial rule on HIV response. Hence, PHC can scale up PLWHIV access to care and to ART initiation. Furthermore, these results also reinforce the need of comprehensive and multi‐sectorial health policies mainly for low‐middle income countries.

## P052

### Salvage antiretroviral therapy for the treatment of HIV‐1 infection in patients failing a first‐line regimen: experience from a national peer advisory committee for physicians in Mexican HIV clinics

**J Sánchez‐Domínguez, J Ramírez‐Hinojosa, A Figueroa‐Morales, H Rivera‐Villegas, S Larrea‐Schiavon, J Calva and the CORESAR Study Group**

Department of Infectious Diseases, Instituto Nacional de Ciencias Médicas y Nutrición “Salvador Zubirán”, Mexico City, Mexico

**Background**

During the last five years, several clinical trials assessing the efficacy of antiretroviral second‐line regimens (2nd‐LR) following virologic failure have been published (*SECOND‐LINE, EARNEST, SELECT,* trials). In our study, we describe the 2nd‐LR recommended to physicians in Mexican HIV clinics by a national board of skilled clinicians, measuring the rate of virologic response (RVR) in diverse salvage strategies.

**Materials and methods**

Patients with HIV‐1 who had confirmed virologic failure (plasma HIV—1 RNA [vRNA] measurement above 200 copies per ml) after 24 weeks or more of first‐line treatment (1st‐LR) and under the care of a physician who had received a recommendation of the salvage regimen by the Mexican Advisory Board for Rational Antiretroviral Usage (CORESAR for its acronym in Spanish), were included and followed up after the 2nd‐LR initiation (observational cohort study). Data were retrieved from a web‐based information system of all individuals receiving care though the Mexican Ministry of Health programme. RVR was defined as the proportion of active participants who, at the end of the follow‐up assessment, had a confirmed vRNA level below 200 copies per ml. Analysis of the RVR was done by a *per protocol* strategy in 103 patients (median time of follow: 50 months [IQR=14 to 64 months]).

**Results**

There were a total of 189 eligible patients. Physicians caring 144 (76%) patients failing a NNRTI‐containing 1st‐LR received the following 2nd‐LR recommendations: (1) ritonavir‐boosted lopinavir (LPV/r) + NtRTIs in 42 (29%) patients, RVR = 85%; (2) LPV/r  +  raltegravir (RAL) in 33 (23%) patients, RVR = 91%; (3) LPV/r  +  RAL + NtRTIs in 23 (16%) patients, RVR=90%; (4) ritonavir‐boosted darunavir (DRV/r) + NtRTIs in 18 (13%) patients, RVR = 83% and (5) other regimens in 28 (20%) patients. Physicians caring 39 (21%) patients failing a PI‐containing 1st‐LR received the following 2nd‐LR recommendations: (1) DRV/r  +  NNRTI + NtRTIs in 12 (31%) patients, RVR=82%; (2) DRV/r  +  RAL + NNRTI+/‐NtRTIs in 8 (20%) patients, RVR=100%; (3) DRV/r  +  NtRTIs in 7 (18%) patients, RVR=57%; and (4) other regimens in 12 (31%) patients.

**Conclusions**

In routine practice, high rates of durable viral control can be achieved following an evidence‐based supported salvage 2nd‐LR recommended by a structured centralized peer‐advisory committee. Comparison of RVR among different 2nd‐LR ought to be interpreted with caution as data were generated through an observational survey.

## P053

### Real‐life use of raltegravir: experience from Hospital Guillermo Almenara, Essalud, Lima, Peru

O Malpartida, G Pérez‐Lazo, J Collins and the Almenara Hospital AIDS Working Group*

Medicina Interna 1‐Infectología, Hospital Guillermo Almenara, Essalud, Lima, Peru

**Background**

Raltegravir is the only INSTI available within the Social Security in Peru. Access to INSTIs in Peru is still limited. The main indication for its use in our institution is as part of rescue regimens. Use of raltegravir initiated in 2008 in our centre. This study evaluates the patterns of use of raltegravir at our hospital.

**Materials and methods**

This is an observational, retrospective, transversal and longitudinal study. HIV‐positive patients older than 15 years‐old receiving a raltegravir‐including combination between July 2008 and May 2017 were included. To determine virological success a minimum period of 24 weeks receiving raltegravir plus at least one measurement of HIV‐viral load was required.

**Results**

Of 1547 patients in the HIV programme during the study period, we identified 155 using raltegravir (10%). Mean age at baseline (start of the raltegravir‐based regime) was 43 years, (SD 13 years). Proportion of females was 34% (53/155). The prescription of raltegravir by year was: 2008, 1% (2/155); 2010, 2% (3/155); 2012, 9% (14/155); 2014, 23% (36/155) and 2016 41% (64/155). Indications for use of raltegravir were: virological failure, 56% (88/155); adverse effects to other ART medications, 15%(23/155); comorbidities 12% (19/155); use as part of a first‐line regime 6% (10/155); GI intolerance to PIs‐6% (9/155), and pregnancy, 4% (6/155). The median time of use of raltegravir was 73 weeks. Data were available for 108 to evaluate virological response: 79 (73%) had a viral load of <37 c/ml; 12 (11%) between 37 and <200 c/ml, and 6 (5.5%), between 200 and <400 c/ml. Our proportion of virological response was 90% (97/108). There were not discontinuations of raltegravir due to adverse effects [1].

**Conclusions**

The use of raltegravir among patients at Hospital Guillermo Almenara is low; but increasing over time. In most cases, indications for use of raltegravir were consistent with the local standard. The proportion of patients responding adequately to a raltegravir‐based regime is high [2,3].

*Other members of the Almenara Hospital AIDS working group: JL Magallanes, PA Castro, RA Castillo, JA Hidalgo, JA Vega, A Irey, LR Illescas, JO Villena, LM Gutiérrez.

**References**

1. van Halsema C, Whitfield T, Lin N, Ashton K, Torkington A, Ustianowski A Five years’ real‐life experience with raltegravir in a large HIV centre. Int J STD AIDS. 2016 Apr;27(5):387‐93.

2. Steigbigel RT, et al, BENCHMRK Study Teams . Raltegravir with optimized background therapy for resistant HIV‐1 infection. N Engl J Med. 2008 Jul 24;359(4):339‐54

3. Rockstroh JK, et al.; STARTMRK Investigators. Durable efficacy and safety of raltegravir vs. efavirenz when combined with tenofovir/emtricitabine in treatment‐naive HIV‐1‐infected patients: final 5‐year results from STARTMRK. J Acquir Immune Defic Syndr. 2013 May 1;63(1):77‐85

## P054

### The importance of the health team in the search for HIV‐infected patients who abandoned treatment

**N Carvalho**

Treatment Reference Center of AIDS and Hepatitis, Town Hall of Diadema, São Paulo, Brazil

**Background**

Several studies show that adherence to treatment is a challenge for HIV patients. Access to antiretroviral therapy (ART) contributes to a nearly normal life expectancy. But many patients give up treatment. The objective of this article is to demonstrate the importance of the health team in search of the supposed patient in abandonment. The approach of each patient is the essential responsibility of the health team.

**Materials and methods**

Between April and December 2017, after checking in the medical records the absence in medical consultations for more than 6 months and the non‐withdrawal of drugs in the pharmacy by 237 patients of the Treatment Reference Center (TRCAH) of AIDS and hepatitis in the municipality of Diadema, some team members were mobilized to look for them and try to rescue them. At first, pharmacy technicians checked the national network system if the patient had withdrawn their medication somewhere in the country. The system shows the dates and where the drug was withdrawn. Second, calls were made to the patient's phone, at different times and days, if they were found, their medical appointments were scheduled. Otherwise, nursing of the health unit closest to the patient's home was accessed to find and summon him.

**Results**

Of the 237 patients, 32% changed their addresses; 19% returned to TRCAH for another reason; in 21% were in abandonment; 13% were not abandoned; 9% were being treated in private health system; 4% died; 2% were discharged because the diagnosis was false positive. After approach, 5% returned to treatment.

**Conclusions**

It was concluded that 50 of the patients were in abandonment, 12 of those were rescued. The success rate was 24%. It is considered that mobilize of the health team is a strategy combined prevention. Actively monitor patient adherence (medical consultations, exams and ART drugs), and facilitate their proximity with the team can generate trust and increase their treatment adherence. This helps achieve the target of UNAIDS 90‐90‐90, and this is also an indication quality of treatment centre.

**Reference**

1. Neide S. S. Carvalho; Maria Laura M. Matos; Daniel Silva A. B. R.; Isaura A. C. Freitas; Fernanda C. R. Da Silva; Luiz A. C. Barros; Luiz A. C. Barra; Alexandre Yamaçake, A. Treatment Reference Center of Aids and Hepatitis (TRCAH), Diadema, Brazil. 2018.

## Viral Hepatitis

## P055

### High rates of medical and psychiatric comorbidities in HIV/HCV co‐infected patients treated with sofosbuvir‐containing regimens in registrational clinical trials

**M Sulkowski^1^, C Cooper^2^, J‐M Molina^3^, S Naggie^4^, A Osinusi^5^, L Stamm^5^, B Massetto^5^, J McNally^6^, D Brainard^5^, J McHutchison^7^, D Wyles^8^, J Rockstroh^9^, D Dieterich^10^, E Bassetti^11^ and A Campos^11^**

^1^Johns Hopkins Hospital, Baltimore, MD, USA. ^2^University of Ottawa, Ottawa, Canada. ^3^Université Paris‐Sorbonne, Hopital Saint‐Louis, Paris, France. ^4^Duke University Medical Center, Durham, NC, USA. ^5^Clinical Research – Liver Disease, Gilead Sciences Inc, Foster City, CA, USA. ^6^Clinical Research – Inflammation/Respiratory, Gilead Sciences Inc, Foster City, CA, USA. ^7^Clinical Executive – Liver Disease, Gilead Sciences Inc, Foster City, CA, USA. ^8^UC San Diego School of Medicine, La Jolla, CA, USA. ^9^Universität Bonn, Bonn, Germany. ^10^Icahn School of Medicine at Mount Sinai, New York, NY, USA. ^11^PH&MA, Gilead Sciences, São Paulo, Brazil

**Background**

HIV/HCV co‐infected patients have a high prevalence of medical and psychiatric diseases which may limit access to HCV therapy. Recent trials of DAA therapy in HIV/HCV co‐infected patients have demonstrated high SVR rates similar to that observed in HCV monoinfection; however the generalizability of these results have been questioned due to the clinical trial setting and the exclusion of certain antiretrovirals. The goal of this analysis was to describe the prevalence of medical and psychiatric disease and DDI interactions in HIV/HCV co‐infected subjects enrolled in Gilead trials.

**Materials and methods**

An integrated analysis of data from HIV/HCV co‐infected patients enrolled in 4 phase 3 registrational trials (PHOTON‐1 and ‐2 [sofosbuvir [SOF] + ribavirin [RBV] for 12 or 24 weeks], ION‐4 [ledipasvir (LDV)/SOF ± RBV for 12 weeks] and ASTRAL‐5 [SOF/velpatasvir (VEL) for 12 weeks] was performed. Patients with severe medical or psychiatric conditions were excluded from these trials. An assessment of demographics, medical history, concomitant medications, safety and efficacy will be presented.

**Results**

A total of 940 patients were included in this analysis; a majority (71%) were enrolled in the United States. Across all studies, there was a high prevalence of hypertension (28%), gastro‐oesophageal reflux disease (GERD) 16% and hyperlipidaemia (14%). A high proportion had psychiatric comorbidities including depression (37%) and anxiety (20%). Prior or ongoing substance abuse was reported in 17% of patients. These rates were similar to that described in a recent analysis of over 18,000 HIV/HCV co‐infected patients in the US (Meyer N et al, ICAAC 2015). In the PHOTON‐2 study which was conducted in Europe, a smaller proportion reported both medical and psychiatric comorbidities. Overall SVR rates ranged from 79% to 96% based on the treatment regimen, similar to that observed in HCV monoinfected patients.

**Conclusions**

The use of interferon‐free DAA regimens has resulted in the ability to successfully treat HIV/HCV co‐infected patients with complex comorbidities in clinical trials which can be generalizable to real world practice.

## P056

### SOF/VEL for 12 weeks results in high SVR12 rates in subjects with negative predictors of response to treatment: an integrated analysis of efficacy from the Astral‐1, Astral‐2 and Astral‐3 studies

**K Agarval^1^, K Patel^2^, D Samuel^3^, M Bourliere^4^, X Liu^5^, Z Younes^6^, L Liu^7^, J McNally^8^, A Osinusi^8^, D Brainard^8^, S Subramanian^9^, N Afdhal^10^, N Cheinquer^11^ and I Dutra^12^**

^1^King's College Hospital, London, UK. ^2^University Health Network Liver Clinic, Toronto, Canada. ^3^Hospital Universitaire Paul Brousse, Villejuif, France. ^4^Hospital Saint Joseph, Marseille, France. ^5^Biology, Gilead Sciences Inc, Foster City, CA, USA. ^6^GastroOne, Germantown, USA. ^7^Biostatistics Inflammation/Respiratory, Gilead Sciences Inc, Foster City, CA, USA. ^8^Clinical Research, Gilead Sciences Inc, Foster City, CA, USA. ^9^Information Management, Gilead Sciences Inc, Foster City, CA, USA. ^10^Beth Israel Deaconess Medical Center, Boston, MA, USA. ^11^Medical Affairs HCV, Gilead Sciences Inc, Foster City, CA, USA. ^12^PH&MA, Gilead Sciences, São Paulo, Brazil

**Background**

The once‐daily fixed‐dose combination tablet of sofosbuvir/velpatasvir (SOF/VEL) was evaluated for the treatment of genotype 1–6HCV infection in three phase 3 studies in patients with and without compensated cirrhosis (ASTRAL‐1, ASTRAL‐2, ASTRAL‐3). Overall SVR12 rates were >95% across all HCV genotypes. This post‐hoc analysis assesses efficacy in patients with traditional negative predictors of response.

**Materials and methods**

This was a retrospective analysis of data from 1035 patients treated with SOF/VEL in the Phase 3 ASTRAL‐1, ASTRAL‐2 and ASTRAL‐3 studies. Presence of cirrhosis was determined by histology, blood tests or transient elastography. Viral load and other clinical and laboratory assessments were determined prior to treatment with SOF/VEL. Prior treatment records were source verified and race was self‐reported by the patient to the investigator.

**Results**

Overall, 21% of patients had cirrhosis, 74% had HCV RNA 800,000 IU/ml, 28% had prior treatment failure, 12% were ≥65 years old and 6% were black. Table 1 provides SVR12 rates by HCV genotype overall and for each patient subgroup. The overall SVR12 rate was 98% and was ≥96% among all subgroups. In general, SVR12 rates were lower in patients with genotype 3 HCV infection compared with other HCV genotypes but were ≥90% across all subgroups.

**Conclusions**

The ASTRAL‐1, ASTRAL‐2 and ASTRAL‐3 studies enrolled a diverse patient population that included a significant number of patients with historically negative predictors of response. There was little effect of these factors on the efficacy of treatment with SOF/VEL for 12 weeks in subjects with genotype 1.

## P057

### Safety and efficacy of treatment with once‐daily ledipasvir/sofosbuvir (90/400 mg) for 12 weeks in genotype 1 HCV‐infected patients with severe renal impairment

**L Eric^1^, C Landis^2^, B Maliakkal^3^, M Bonacini^4^, G Ortiz‐Lasanta^5^, J Zhang^6^, E Mogalian^7^, S De‐Oertel^8^, A Osinusi^8^, D Brainard^8^, J McHutchison^9^, S Flamm^10^, S Gordon^11^, E Gane^12^ and R Chirino^13^**

^1^UT Health San Antonio, Texas Liver Institute, San Antonio Texas, USA. ^2^UW Medical Center, Seattle, WA, USA. ^3^The University of Tennessee, Memphis, TN, USA. ^4^Mission Gastroenterology & Hepatology, University of California, San Francisco, CA, USA. ^5^Fundación de Investigación, San Juan, Puerto Rico. ^6^Biostatistics, Gilead Sciences Inc, Foster City, CA, USA. ^7^Clinical Pharmacology, Gilead Sciences Inc, Foster City, CA, USA. ^8^Clinical Research, Gilead Sciences Inc, Foster City, CA, USA. ^9^Gilead Sciences Inc, Foster City, CA, USA. ^10^Gastroenterology, Northwestern University, Chicago, IL, USA. ^11^Gastroenterology, Henry Ford Health System, Detroit, MI; USA. ^12^Hepatology, Auckland Clinical Studies Ltd, Auckland, New Zealand. ^13^PH&MA, Gilead Sciences, Mexico City, Mexico

**Background**

Despite higher concentrations of the primary circulating sofosbuvir (SOF) metabolite, GS‐331007, in patients with severe renal impairment, retrospective case series and claims database analyses have suggested substantial use of ledipasvir (LDV)/SOF in this population with no untoward effects described. The current study evaluated the safety, efficacy, and pharmacokinetics (PK) of LDV/SOF (90/400 mg) once‐daily for 12 weeks in patients with genotype (GT) 1 HCV‐infection and severe renal impairment.

**Materials and methods**

Treatment naïve or experienced patients with or without compensated cirrhosis and creatinine clearance (CLcr) ≤ 30 ml/min (Cockcroft‐Gault equation), not on dialysis, received open‐label treatment with LDV/SOF once daily for 12 weeks. Virologic response, pharmacokinetics (PK), and safety, including echocardiograms, were assessed.

**Results**

Of the 18 patients enrolled and treated, the majority were male (67%), 10 (56%) were African‐American, 8 (44%) had BMI ≥30 kg/m^2^ and mean (range) CLcr at baseline was 24.9 (9.0 to 39.6) ml/min. In terms of liver disease characteristics, all 18 had GT1 HCV infection (14 GT1a and 4 GT1b), 14 (78%) were treatment naïve, and 2 (11%) had cirrhosis. All patients completed 12 weeks of LDV/SOF treatment. There were no early discontinuations or any on‐treatment virologic failures. The SVR12 rate is 100% (18/18). Plasma concentrations of the terminal SOF metabolite GS‐3310007 were approximately sixfold higher than in the LDV/SOF Phase 3 trials. SOF and LDV concentrations were similar to those observed in patients with normal, mild or moderate RI. The most common adverse events (AEs) were fatigue (22%), headache (22%) and hyperkalemia (22%). Six serious AEs were reported among 4 patients (22%), including 2 renal events; no SAEs were considered related to study drugs. There were no treatment‐related cardiac AEs, including bradycardia, and no meaningful changes in QTc intervals or other parameters. 

**Conclusions**

Treatment with LDV/SOF (90/400 mg) for 12 weeks in genotype 1 patients with and without cirrhosis and severe renal impairment resulted in 100% SVR4 rate. The regimen was safe and well‐tolerated with no treatment discontinuations and no treatment‐related SAEs.

## P058

### Genetic diversity of the hepatitis B virus strains in Cuba: absence of West‐African genotypes despite the transatlantic slave trade

**L Rodriguez Lay^1^, M Bello Corredor^1^, M Montalvo Villalba^1^, S Sariego Frometa^1^, M Sanchez Wong^1^, L Valdes Alonso^2^, M Samada^3^, A Sausy^4^, J Hübschen^4^ and C Muller^4^**

^1^Virology, Institute of Tropical Medicine Pedro Kouri (IPK), Havana, Cuba. ^2^Hospital Center, Institute of Tropical Medicine Pedro Kouri (IPK), Havana, Cuba. ^3^Gastroenterology, Centro de Investigaciones Médico – Quirúrgicas (CIMEQ), Havana, Cuba. ^4^Infection and Immunity, Luxembourg Institute of Health, Luxembourg

**Background**

Cuba is an HBsAg low‐prevalence country with a high coverage of anti‐hepatitis B vaccine. Its population is essentially the result of the population mix of Spanish descendants and former African slaves. Information about genetic characteristics of Hepatitis B Virus (HBV) strains circulating in the country is scarce. The aim of this work was to investigate the genetic diversity of the HBV circulating in Cuba and its relationship with worldwide strains.

**Materials and methods**

The HBV genotypes/subgenotypes, serotypes, mixed infections and S gene mutations of 172 Cuban HBsAg and HBV‐DNA positive patients were determined by direct sequencing and phylogenetic analysis.

**Results**

Phylogenetic analysis of HBV S gene sequences showed a predominance of genotype A (92.4%), subgenotype A2 (84.9%) and A1 (7.6%). Genotype D (7.0%) and subgenotype C1 (0.6%) were also detected but typical (sub)genotypes of contemporary West‐Africa (E, A3) were conspicuously absent. All genotype A, D and C strains exhibited sequence characteristics of the adw2, ayw2 and adrq serotypes respectively. Thirty‐three (19.1%) patients showed single, double or multiple point mutations inside the Major Hydrophilic domain associated with vaccine escape and eighteen (10.5%) patients had mutations in the T‐cell epitope. One patient had an HBV A1/A2 mixed infection.

**Conclusions**

This genetic study of Cuban HBV viruses revealed only strains that were interspersed with strains from particularly Europe, America and Asia. The absence of genotype E supports previous hypotheses about an only recent introduction of this genotype into the general population in Africa. The presence of well‐known vaccine escape (3.5%) and viral resistance mutants (2.9%) warrants strain surveillance to guide vaccination and treatment strategies.

## P059

### Analysis of risk factors associated with hepatitis B infection in Paraná correctional institutions in Brazil

**L Ferreto^1^, F Follador^1^, A Vieira^1^, K Bento Casaril^1^, H Coelho^2^, R Torres^3^, J Frois^4^, G Amaral^5^ and R Yamada^1^**

^1^Center for Health Sciences, State University of West Paraná, Francisco Beltrão, Brazil. ^2^Clinical, Toxicological and Bromatological, University of São Paulo, Francisco Beltrão, Brazil. ^3^Penitentiary Department, State Department of Public Security and Penitentiary Administration of Paraná, Curitiba, Brazil. ^4^Penitentiary Department, State Department of Public Security and Penitentiary Administration of Paraná, Londrina, Brazil. ^5^Epidemiology, State Department of Health, Maringá, Brazil

**Introduction**

The incarcerated population is 726,712 prisoners in Brazil [1]. Hepatitis B virus (HBV) infection in prisoners reaches one of the highest prevalences among specific population subgroups, with rates of up to 54.5%, already described in Brazil [2]. People deprived of their liberty are considered to be at high risk for sexually transmitted diseases due to favourable conditions in prison for the spread of diseases. The State of Paraná has the third largest population of prisons in Brazil, representing 7.12% of this population and has sought to implement healthcare policies for those convicted under the National Health Plan in the Penitentiary System (PNSSP), but the rate of hepatitis B infection [3]. The objective of this study was to estimate the prevalence of HBV markers and their risk factors in the male prison population of correctional institutions in Paraná, Brazil.

**Materials and methods**

Cross‐sectional epidemiologic survey for hepatitis B virus (HBV) infection held in 11 male prisons in Paraná in the period of May 2015 to December 2016. The State of Paraná presents 23 closed system male correctional facilities, with a jail population of 16.657 men incarcerated in closed system. The stages of the investigation included counselling, information about intervention, orientation about sexually transmitted infections, informed consent for the data gathering and blood sampling for the HBV test performed in a certified laboratory. Enzyme‐linked immunosorbent assay (ELISA) was used to diagnose HBV infection (HBsAg, anti‐HBs and total anti‐HBc). Data were analysed using univariate and multivariate techniques.

**Results**

The overall prevalence for HBV markers in inmates was 11.9% (IC95%: 10.9 to 12.8), 135 men infected. In univariate analysis, HBV infection was associated with age>30 years, tattooing, history of tattooing in prison, only one passage through in the prison system, body piercing, sex with drug users and previous illicit drug use, being *p*‐value <0.05 considered significant. Multivariate model, HBV infection was associated with age > 30 years (OR = 5.03; IC 95%: 3.07 to 8.25), previous injecting drug use (OR = 1.76; IC 95%: 1.14 to 2.73) and tattoo (OR = 1.58 IC 95%: 1.02 to 2.46).

**Conclusions**

The reduction of these indices depends on public policies that include vaccination, early diagnosis, harm reduction in the use of drugs and the adequate treatment of individuals with sexually transmitted infections, being offered in the same way for the incarcerated population and for the population in general.

**References**

1. Brasil.(2017). Ministério da Justiça e Segurança Pública. Levantamento Nacional de Informações Penitenciárias Atualização ‐ Junho de 2016. Departamento Penitenciário Nacional, 65 pgs. https://www.conjur.com.br/dl/infopen-levantamento.pdf

2. Silva, Andréia Alves de Sena, et al. (2017). Prevalência de hepatite B e fatores associados em internos de sistema prisional. Accta Paul. Enferm.,  30 (1), 66‐72. https://doi.org/10.1590/1982-0194201700010.

3. Brasil. (2004). Ministério da Saúde. Plano Nacional de Saúde no Sistema Penitenciário. Brasília (DF): Ministério da Saúde; 64 pgs. http://bvsms.saude.gov.br/bvs/publicacoes/cartilha_pnssp.pdf

## P060

### Anti‐HCV seroprevalence and risk factors of HCV infection in penitentiaries in Paraná, Brazil.

**L Ferreto^1^, F Follador^1^, A Vieira^1^, H Coelho^2^, R Yamada^1^, R Torres^3^, J Frois^4^ and G Amaral^5^**

^1^Center for Health Sciences, State University of West Paraná, Francisco Beltrão, Brazil. ^2^Clinical, Toxicological and Bromatological, University of São Paulo, Ribeirão Preto, Brazil. ^3^Penitentiary Department, Security and Penitentiary Administration of Paraná, Curitiba, Brazil. ^4^Penitentiary Department, Security and Penitentiary Administration of Paraná, Londrina, Brazil. ^5^Epidemiology, State Department of Health, Maringá, Brazil

**Background**

The penitentiary system in Brazil presents serious problems of overpopulation [1]. According to data from the Ministry of Justice in 2016 there were 726,712 inmates. In that same year, in Paraná, the number was 51,700 prisoners, of these around 19,700 in the closed system of imprisonment [2]. The severity of these data are accentuated by the fact that the incarcerated population is considered a high‐risk group for sexually transmitted diseases because of the favourable conditions found in prison for the spread of diseases [3]. The objective of this study was to estimate the prevalence of HCV markers and their risk factors in the male prison population of correctional institutions in Paraná, Brazil.

**Materials and methods**

Cross‐sectional epidemiologic survey for anti‐HCV infection held in 11 male prisons in Paraná in the period of May 2015 to December 2016. The stages of the investigation included counselling, information about intervention, orientation about sexually transmitted infections, informed consent for the data gathering and blood sampling for the anti‐HCV test performed in a certified laboratory. Reactive cases of anti‐HCV were considered as hepatitis C. Odds ratio and logistic regression were used for data analysis and P‐value.

**Results**

1.192 men were addressed total. 1.133 (95%) were subjected to a diagnosis for the anti‐HCV test. The estimated predominance of the infection by HCV from this evaluation onwards was of 2.7% (interval of 95% [CI]: 1.9% to 3.8%), 30 men infected. The integrated analysis identified HIV infection and hepatitis C in two men (estimate predominance of 0.18% (95% CI: 0.0% to 0.42%)). The independent effects of the associated factors for HCV were age over 30 years (OR: 4.03 [1.61 to 10.07]), frequency in the prison system (OR: 2.58 [1.02 to 6.52]) and the use of injectable drugs (OR: 7.32 [3.36 to 15.92]).

**Conclusions**

The prevalence of hepatitis c in the prison population is higher than in the free population; reducing the spread of HCV infection in prisons may occur through investments for anti‐HCV screening, early diagnosis that contributes to a better prognosis of the disease and with reflections on the quality of life. Education and health is a practice that should receive investments, since targeting of infected individuals reduces the risk of HCV transmission between prisoners and in the community.

**References**

1. Brasil. (2017). Ministério da Justiça e Segurança Pública. Levantamento Nacional de Informações Penitenciárias Atualização ‐ Junho de 2016. Departamento Penitenciário Nacional, 65 pgs. https://www.conjur.com.br/dl/infopen-levantamento.pdf

2. Brasil. (2004). Ministério da Saúde. Plano Nacional de Saúde no Sistema Penitenciário. Brasília (DF): Ministério da Saúde; 64 pgs. http://bvsms.saude.gov.br/bvs/publicacoes/cartilha_pnssp.pdf

3. Dolan, K. et al. (2016). Global burden of HIV, viral hepatitis, and tuberculosis in prisoners and detainees. HIV and related infections in prisoners 1. The Lancet, 388: 1089 – 1102 pgs.

## P061

### Perceptions of hepatitis B and hepatocellular carcinoma among Blacks in South Florida

**J Gonzalez‐Diaz^1^, N Schaefer Solle^2^, P Martin^1^, E Kobetz^2^ and P Jones^1^**

^1^Department of Medicine, Division of Hepatology, Sylvester Comprehensive Cancer Center, University of Miami Miller School of Medicine, Miami, FL, USA. ^2^Department of Medicine, Sylvester Comprehensive Cancer Center, University of Miami Miller School of Medicine, Miami, FL, USA

**Introduction**

Worldwide, hepatitis B virus (HBV) infection accounts for over 50% of cases of Hepatocellular Carcinoma (HCC), a leading cause of cancer‐related mortality with significant racial disparities. In our Black patients with HCC, HBV infection was more prevalent than in Whites and Hispanics with HCC. Furthermore, only one third of Black patients with HBV received treatment prior to their HCC diagnosis. Presumably, most were not diagnosed with HBV until their HCC diagnosis. We aimed to understand perceptions of HBV, cirrhosis and HCC among Blacks born in the US or Haiti residing in our catchment area.

**Materials and methods**

Through Sylvester Comprehensive Cancer Center's Behavioral Community Shared Resource Core's established partnerships with key stakeholders, participants were recruited via word of mouth, email listservs and social media. Individuals with HBV, hepatitis C, cirrhosis or HCC were excluded. There were four groups with 10 participants each: Haitian Women (HW), US‐born Black Women (UW), Haitian Men (HM) and US‐born Black Men (UM). Focus groups were conducted in English or Haitian Creole and transcripts were analysed by the BCSR to identify recurrent themes using the principles of framework and grounded theory analysis. Participants completed a baseline questionnaire, which included the Short Assessment of Health Literacy (SAHL). Using univariate, bivariate and Pearson's chi‐squared analyses, we describe participant characteristics by group.

**Results**

In total, 40 individuals participated in the study with a median age of 53.5 years. The median SAHL score was 13, and was lowest in HW and HM compared to UW and UM, *p* 0.05; scores ≤14 indicate low health literacy (Table 1). As well, 52.6% of participants reported that they had heard of HBV. Figure 1, also depicts the stigma or embarrassment of having hepatitis and confusion over the modes of hepatitis transmission (Figure 2) that arose during the interview. Although participants did not have insight into HCC, they did perceive that the cause of observed disparities is due to limited healthcare access compared to Whites.

**Table:** Abstract P061‐Table 1. Participant demographics

	Overall (n = 40)	Haitian Women (n = 10)	US‐Born Women (n = 10)	Haitian Men (n = 10)	US‐Born Men (n = 10)	*p*‐value
Age, years	53.5 (45.5 to 58.5)	44 (37 to 48)	58.5 (55 to 60)	54 (51 to 59)	53 (50 to 56)	0.03
US‐born, n(%)	20 (51.3)	1 (10)	9 (100)	1 (10)	9 (90)	<0.001
Marital Status						0.11
*Married*	14 (35)	6 (60)	0	6 (60)	2 (20)	
*Divorced*	6 (15)	0	3 (30)	0	3 (30)	
*Widowed*	2 (5)	0	1 (10)	1 (10)	0	
*Separated*	9 (22.5)	2 (20)	2 (20)	2 (20)	3 (30)	
*Never Married*	8 (20)	2 (20)	4 (40)	1 (10)	1 (10)	
*Unmarried Partner*	1 (2.5)	0	0	0	1 (10)	
Employment Status						0.54
*Employed for wage*	13 (33.30)	5 (55.6)	3 (30)	3 (30)	2 (20)	
*Self‐employed*	8 (20.5)	1 (11.1)	1 (10)	2 (20)	4 (40)	
*Out of work >1 year*	7 (18)	1 (11.1)	1 (10)	3 (30)	2 (20)	
*Out of work <1 year*	2 (5.1)	1 (11.1)	0	0	1 (10)	
*Student*	5 (12.8)	1 (11.1)	3 (30)	1 (10)	0	
*Unable to work*	4 (10.3)	0	2 (20)	1 (10)	0	
Highest Education Level						0.05
*Grades 1‐8*	5 (12.8)	2 (20)	0	3 (33.3)	0	
*Grades 9‐11*	6 (15.4)	0	4 (40)	1 (11.1)	1 (10)	
*Grade 12/GED*	16 (41)	3 (30)	5 (50)	2 (22.2)	6 (60)	
*College (1 to 3 years)*	7 (18)	4 (40)	1 (10)	0	2 (20)	
*College Graduate*	3 (7.7)	1 (10)	0	2 (22.2)	0	
*Grad/Adv Degree*	2 (5.1)	0	0	1 (11.1)	1 (10)	
Median SAHL Score	13 (12 to 15)	12.5 (11 to 13)	15.5 (12 to 16)	12.5 (11 to 13)	14 (14 to 17)	0.05
Seen Hepatologist, n(%)	2 (5)	0	0	2 (20)	0	0.11
Alcoholic Drinks/Day, n(%)						0.09
*Never*	17 (44.7)	9 (90)	3 (30)	2 (25)	3 (30)	
*<1/day*	11 (29)	1 (10)	2 (20)	3 (37.5)	5 (50)	
*1/day*	3 (7.9)	0	2 (20)	1 (12.5)	0	
*2/day*	4 (10.5)	0	2 (20)	0	2 (20)	
*3 to 5/day*	0	0	0	0	0	
*>5/day*	1 (2.6)	0	0	1 (12.5)	0	
*Prefer not to comment*	2 (5.3)	0	1 (10)	1 (12.5)	0	
Heard of HBV‐ prior to study, n(%)						<0.01
*Yes*	20 (52.6)	2 (20)	10 (100)	3 (33.3)	5 (55.6)	
*No*	17 (44.7)	8 (80)	0	5 (55.6)	4 (44.4)	
*Don't Know*	1 (2.6)	0	0	1 (11.1)	0

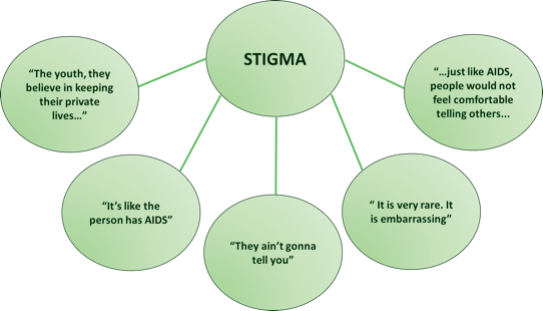

**Abstract P061–Figure 1. Stigma of HBV among study sample (n = 40).**

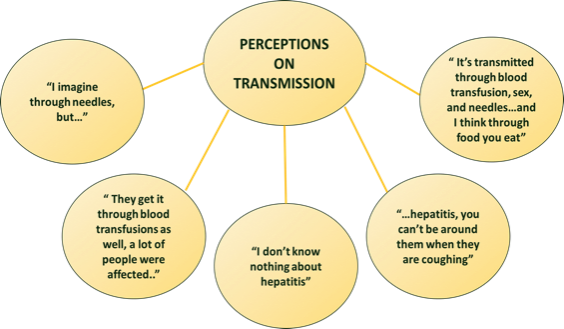

**Abstract P061–Figure 2. Perceptions on transmission among study sample (n = 40).**

**Conclusions**

Blacks in our catchment area have limited knowledge of HBV and its link to HCC. Also, there are misconceptions about HBV transmission. Stigma against those with HBV, limited access to healthcare and suboptimal health literacy may limit detection of HBV in persons at risk, leading to increased HCC incidence. Future research will test interventions targeted at increasing detection of HBV in these vulnerable populations.

## P062

### Hepatitis B: detection rates and vaccination coverage in Brazil in 2016

**F Alvarenga Pinto, M Santos, R Almeida Coelho, S Monzani Vivaldini, G Mendes Pereira and A Schwartz Benzaken**

Department of Surveillance, Prevention and Control, Ministry of Health of Brazil, Brasília, Brazil

**Background**

Approximately 257 million people are living with hepatitis B virus (HBV) infection. In Brazil, since 1999, there have been detected 212,031 cases, with detection rates varying among the five regions in the country. Hepatitis B vaccine – the main form of HBV control – was implemented in 1998 in the whole country, gradually expanded to cover age's groups, and became universal in 2016. We aim to investigate the relationship between vaccination coverage and detection rates.

**Materials and methods**

Cross‐sectional study of hepatitis B cases registered in Notifiable Diseases Information System in 2016 in Brazil. We calculated detection rates based on demographic data from the Ministry of Health Department of Informatics. Cumulative vaccination coverage data were obtained from the National Immunization Program. We applied Spearman rank correlation to evaluate the relationship between vaccination coverage and detection rates.

**Results**

In 2016, 14,199 cases of hepatitis B were detected, with a detection rate of 6.9 (cases per 100,000 population); the cumulative vaccination coverage for the general population was 56.7%. In the group‐aged ≤4 years, and 5 to 14 years, coverage was over 82% and detection rate below 1.8 in all regions. Among youngsters aged 15 to 24 years, the North region had the highest detection rate (6.5), although it also presented the highest vaccination coverage. Among people aged 25 to 29 years, again the North region had the highest vaccination coverage (50.6%). However, the detection rate was the second largest (18.2) in Brazil. Among adults aged ≤ 50 years, coverage was below 26%, and the North region presented the highest detection rate (15.9), followed by the Southeast (14.5) (Table 1). With the exception of the North region, the correlations between vaccination coverage and detection rates were negative, with *p*‐values < 0.05.

**Table:** Abstract P062‐Table 1. Cumulative vaccination coverage and hepatitis B detection rates per 100,000 population, per age group, per region. Brazil, 2016. Source: Ministry of Health of Brazil

Age Group	Vaccination coverage ‐NORTH	Detection rate ‐ NORTH	Vaccination coverage ‐ NORTHEAST	Detection rate ‐ NORTHEAST	Vaccination coverage ‐ SOUTHEAST	Detection rate ‐ SOUTHEAST	Vaccination coverage ‐ SOUTH	Detection rate ‐ SOUTH	Vaccination coverage ‐ CENTER WEST	Detection rate ‐ CENTER WEST
0 to 4	82.3	1.2	88.6	0.3	94.9	0.4	93.8	1.7	93.7	1.2
5 to 14	99.3	0.8	95.2	0.1	91.4	0.1	87.7	0.5	91.3	0.1
15 to 24	105.1	6.5	83.7	1.5	81.6	2.6	86.3	5.4	78.9	4.3
25 to 49	50.6	18.2	30.5	4.0	34.4	8.4	41.9	24.8	37.3	13.7
50+	23.5	15.9	15.0	3.1	15.7	6.9	14.5	23.1	25.4	12.4

**Conclusions**

Findings indicate that low detection rates among younger people is associated to high vaccination coverage while, especially among adults and the elderly, low vaccination coverage is related to high detection rates. However, there are differences among regions, and the North region of Brazil does not follow this trend. Estimated vaccination coverage is based on information related to administered doses, which is subject to mistakes in the registries. In addition, high detection rates may be due to the late diagnosis of this silent disease. It is necessary to keep the Viral Hepatitis Fighting Plan in Brazil's North region and to strengthen prevention measures in order to increase vaccination coverage mainly among adults and the elderly.

## P063

### Android applications for viral hepatitis diagnosis, research and training

**E Guadis Salazar^1,2^, L Rodriguez Lay^2^, M Montalvo Villalba^2^, M Bello Corredor^2^, D Lopez Hernandez^2^, B Marrero Sanchez^2^ and W Sanchez Puente^2^**

^1^Cuban Society of Microbiology and Parasitology, Havana, Cuba. ^2^Virology, Institute of Tropical Medicine Pedro Kouri (IPK), Havana, Cuba

**Background**

Informatics and Communications Technology (ICTs) are valuable tools for information exchange. ICTs can contribute to the technological and scientific development, teaching, and learning and in general to all aspect of modern society. Among ICTs, cell phone technology has the necessary qualities that allow its use in laboratories and in the clinical practice to facilitate the daily work. The aim of this work was to design and develop simple, didactic, dynamic, interactive and flexible Android applications for viral hepatitis diagnosis, research and teaching.

**Materials and methods**

For the development of the applications a flexible methodology with the aim to adapt to new platform changes was use. The Android Studio Programming tool was used for the design and development of the applications. Applications can be executed in devices using Android 4.0 platform or later.

**Results**

It was successfully designed and developed three applications: LabCalc which allows calculation of Polymerase Chain Reaction (PCR) Mixes, reagents dilutions (primers) and evaluation of diagnosis assays; HepText which includes relevant information in regard to the aetiology, diagnosis, clinical and epidemiological aspects of viral hepatitis; and HepDiag which facilitate and aid in the diagnosis of viral hepatitis and include a calculator for prognostic models in liver disease (Child‐Pugh and Meld/Peld) as well as serum models for diagnosing liver fibrosis (APRI and Fib‐4).

**Conclusions**

The applications LabCalc, HepText and HepDiag were successfully designed and developed. These applications facilitate reagents calculation, the assessment of the analytical performance of in house diagnostics assays standardized in the laboratory, improves the teaching and learning of viral hepatitis and help physicians in the better diagnosis, prognosis and treatment of viral hepatitis.

## P064

### Emergence of the transmitted resistance of HIV‐1 to antiretroviral drugs in Cuban patients during the period 2009–2016

**L Machado Zaldivar^1^, H Díaz Torres^2^, M Blanco de Armas^3^, M Dubed Echevarria^1^, N Ruiz^1^, L Martínez^2^, E Silva Cabrera^4^, L López Rizo^1^, D Romay Franchi^1^, N Valdés de Calzadilla^5^, C Nibot^5^, C Rivero Martínez^1^, J Joanes Fiol^6^, I Cancio^6^ and M Rodríguez Acosta^1^**

^1^Molecular Biology, AIDS Research Laboratory, San José de las Lajas, Mayabeque, Cuba. ^2^Clinical, Hermanos Ameijeiras Hospital, Havana, Cuba. ^3^Molecular Biology, AIDS Research Laboratory, Havana, Cuba. ^4^Commercial, Immunoassays Center, Havana, Cuba. ^5^Diagnostic, AIDS Research Laboratory, San José de las Lajas, Mayabeque, Cuba. ^6^National STI/HIV/AIDS Program, Ministry of Public Health, Havana, Cuba

**Background**

The emergence of antiretroviral‐resistant HIV‐1 variants compromises the first‐line treatment regimens and the achievement of worldwide 90‐90‐90 UNAIDS target. Since 2009, the surveillance of HIV‐1 transmitted resistance to antiretroviral drugs in newly diagnosed patients was introduced in Cuba. The aim of the present study is to evaluate the behaviour of transmitted resistance of HIV‐1 in Cuban patients of recent diagnosis and without antiretroviral treatment.

**Materials and methods**

A cross‐sectional descriptive and retrospective study was carried out that included 469 samples of HIV‐1 Cuban patients diagnosed in the 2009 to 2016 period. The viral subtype was determined by phylogenetic analysis. Transmitted drug resistance was determined using the CPR tool v6.0. Some clinical and epidemiological variables were evaluated. A possible association of viral variants with sexual preference, progression of the disease and resistance to antiretroviral drugs was determined. For statistical analysis, was the software package R and SPSS v19 were used.

**Results**

The predominant HIV‐1 genetic variants were subtype B (27%), CRF 20, 23, 24_BG (23.5%) and CRF19_cpx (20.2%). An increase of the URF in the newly diagnosed patients was described in the period 2015 to 2016 (24.5%, *p* < 0.05). The most frequent URFs presented the combinations CRF19_cpx/B, BF1, BC and CRF19_cpx/CRF18_cpx. Overall, 19% of the patients presented viruses with any mutation associated with the transmitted resistance of HIV‐1 to ARVs (10.4% to NRTI, 12.8% to NNRTI, 2.8% to PI). The most frequent mutations were K103N/S and Y181C in the family of NNRTI, and M184V/I and D67N in the family of NRTI, which decrease the susceptibility to NVP, EFV and 3TC, AZT, respectively. The resistance to the NNRTI increased in the period 2015 to 2016 with respect to the 2009 to 2012 period (16.9% vs. 11.9%). No significant differences were found between the genetic variants of HIV‐1, the mutations associated with transmitted drug resistance, and the clinical progression of the disease in the sample studied.

**Conclusions**

A high genetic diversity of HIV‐1 and emergence of resistance transmitted to ARV in untreated population was described. These results demonstrate the need to continue epidemiological surveillance and search for new therapeutic strategies that contribute to accelerated compliance of worldwide 90‐90‐90 UNAIDS target.

## Virology and Immunology

## P065

### Plasma cytokines levels in patients with disseminated Kaposi sarcoma with syphilis and infectious AIDS‐defining events.

**P Volkow^1^, L Ramon‐Luing^2^, L Chávez‐Galán^2^, P Cornejo‐Juárez^1^, D Vilar‐Compte^1^, R Ocaña‐Guzmán^2^ and B Islas‐Muñoz^1^**

^1^Infectious Diseases, Instituto Nacional de Cancerologia, Mexico City, Mexico. ^2^Immunology Laboratory, Instituto Nacional de Enfermedades Respiratorias, Mexico City, Mexico

**Background**

Kaposi sarcoma (KS) is a cytokine‐mediated angioproliferative disease. The role of several cytokines in KS co‐infected patients has not been described [1,2,3]. Cytokines levels (IL‐6, Il‐10, IL‐1beta and IFN‐gamma) were measured in plasma from patients with disseminated KS at baseline, at four and at 12 weeks of follow‐up. Two groups were compared with and without syphilis, and other group with and without infectious AIDS Defining event (IADE).

**Materials and methods**

A prospective and observational study was conducted from October 2015 to November 2017. A complete clinical evaluation and active search of co‐infections including laboratory, thorax and abdominal CT‐scan, gastrointestinal endoscopy, VDRL, HVB and HVC serology, blood marrow culture and tissue biopsy if needed was performed. All patients with syphilis were treated during the first four‐weeks; by 12th week all were on combined antiretroviral therapy as well as on treatment of co‐infection. Plasma cytokines levels were measured by ELISA assay. We compared groups using Mann‐Whitney test considering *p* < 0.05 as statistically significant.

**Results**

Twenty patients were included in this report 35% (n = 5) had syphilis, six (30%) had IADE, 3 (15%) with disseminated *Mycobacterium Avium* Complex, 2 (10%) with disseminated histoplasmosis and one with *Penicillium* lung infection. IL‐1b was lower to 2 pg/ml in all samples according to a previous report on patients with KS. The median levels of IL‐6, IL‐10 and IFN‐ gamma at baseline and at week 12, in patients with and without syphilis, without any co‐infection and with IADE are shown (Table 1).

**Conclusions**

IL‐6 in patients with KS and syphilis were significantly lower at baseline. In patients with IADE IL‐6 was significantly higher at baseline, decreasing parallel to treatment; contrary IFN‐ gamma was significantly increased suggesting that IFN‐ gamma is up‐regulated once patients start anti‐infectious therapy no related to syphilis infection. KS is a complex disease; co‐infections. KS is a complex disease that requires diverse clinical and immunological factors to appear. Co‐infections appear to show a different pattern of some of the cytokines studied may play an important role in the pathogenesis of the disease.

**Table:** Abstract P065‐Table 1. Plasma cytokine levels in patients with and without syphilis and infectious AIDS defining events at baseline, 4 weeks and after 12 weeks’ follow‐up

Plasma cytokine levels (pg/ml)	Syphilis positive (n = 7)	Syphilis negative (n = 13)	*p* value	Without Infectious AIDS defining event (n = 13)	Infectious AIDS defining event (n = 7)	*p* value
IL‐6 baseline	12.4	20.7	0.02	16.1	35.8	0.03
IL‐6 4 weeks	13.7	22.6	NS	22.4	31.4	NS*
IL‐6 12 weeks	10.1	13.9	NS	11.8	16.6	NS*
IL‐10 baseline	12.8	18.6	NS	11.3	20.6	NS*
IL‐10 4 weeks	7.2	9.5	NS	19.1	8.7	NS*
lL‐10 12 weeks	7.6	9.9	NS	8.9	17.4	NS*
IFN‐gamma baseline	8.06	8.4	NS	10.1	9.3	NS*
IFN‐gamma 4 weeks	5.6	8.27	NS	5.6	17.6	0.03
IFN‐gamma 12 weeks	5.6	5.56	NS	5.6	14.5	0.02

*NS: non‐significant

**References**

1. Gantt S, Casper C. Human herpesvirus 8‐associated neoplasms: the roles of viral replication and antiviral treatment. Curr Opin Infect Dis. 2011; 24: 295‐301. https://doi.org/10.1097/qco.0b013e3283486d04.

2. Dezube BJ. Clinical presentation and natural history of AIDS‐related Kaposi's sarcoma. Hematol Oncol Clin North Am 1996; 10:1023.

3. Uldrick TS, Wang V, O'Mahony D, Aleman K, Wyvill KM, Marshall V et al. An interleukin‐6‐related systemic inflammatory syndrome in patients co‐infected with Kaposi sarcoma‐associated herpesvirus and HIV but without Multicentric Castleman disease. Clin Infect Dis. 2010 Aug 1;51(3):350‐8.

## P066

### Co‐receptor tropism determined by genotypic assay in HIV‐1 non‐B subtypes circulating in Cuba. Implications for pathogenesis and maraviroc resistance

**V Kourí^1^, L Pérez^1^, Y Martínez^1^, Y Aleman^1^, D Diaz^1^, R Han^1^, Y Pintos^1^, M Mendez^1^, Y Soto^1^, Y Baños^1^, Y Caturla^1^, C Fonseca^2^, J Pérez^2^ and U Hengge^3^**

^1^Virology, Institute of Tropical Medicine Pedro Kourí, Havana, Cuba. ^2^Medicine, Institute of Tropical Medicine Pedro Kourí, Havana, Cuba. ^3^Dermatology, Hautzentrum, Dusseldorf, Germany.

**Background**

The V3 loop of the HIV‐1 envelope gene is involved in binding to the chemokine receptors CCR5 and CXCR4, thus determining viral tropism.

**Materials and Methods**

With the aim of genetically characterizing the C2V3 env region of HIV‐1 samples from Cuban patients, naïve to Maraviroc therapy, 115 plasmas were taken in the period 2014 to 2016 and analysed by sequencing of the C2V3 region. HIV‐1 subtype, the prediction of the co‐receptor usage, the viral mutations associated to Maraviroc resistance as well as the association of the subtype with clinical, epidemiological, virological and immunological variables were analysed.

**Results**

The subtypes detected in the C2V3 region were CRF20, 23, 24_BG (35 patients, 30.4%), Subtype B (33 patients, 28.7%), CRF19_cpx (30 patients, 26.1%), CRF18_cpx (10 patients, 8.7%) and others (7 patients, 6.1%). Overall, 60% of the viruses exhibited R5 phenotype, 14.8% were R5X4 and 25.2% were X4. Interestingly CRF19_cpx virus was associated with having phenotype X4 (46.7%, *p* = 0.0047, OR: 3.96, CI: 1.59 to 9.84), with infection in young individuals (39.1%, *p* = 0.025, OR: 3548; CI: 1136 to 11,077) and with higher values of viral load (*p*≤0.05). The comparison of the amino acid sequences of the V3 loop showed differences between the B and non‐B subtypes (*p* = 0.0001). Mutations reported to be associated with Maraviroc resistance were detected in 75.7% of the samples, in positions 11 (6.1%), 13 (49.6%), 25 (6.1%), 316 (7.0%), 323 (11.3%) and 319 (3.5%) of Gp120, particularly in the recombinant forms CRF19_cpx and CRF_BGs. HIV variants that use the CXCR4 co‐receptor were associated with more than 10 years of diagnosis, with older individuals, in the AIDS stage, with low CD4 counts and higher viral load levels (*p* < 0.05).

**Conclusions**

The results support the hypothesis previously stated that CRF19_cpx viruses could be more pathogenic and would have limitations for the use of Maraviroc. The high rate of mutations associated to MVC among non‐B Cuban subtypes should be further studied.

